# Family-based cognitive behavioral therapy versus family-based psychoeducation and relaxation training for obsessive-compulsive disorder in children and adolescents: a randomized clinical trial (TECTO)

**DOI:** 10.1007/s00787-025-02797-4

**Published:** 2025-07-31

**Authors:** Anne Katrine Pagsberg, Nicole Nadine Lønfeldt, Christine Lykke Thoustrup, Nicoline Løcke Jepsen Korsbjerg, Camilla Funch Uhre, Sofie Heidenheim Christensen, Valdemar Funch Uhre, Anna-Rosa Cecilie Mora-Jensen, Melanie Ritter, Linea Pretzmann, Helga Kristensen Ingstrup, Birgitte Borgbjerg Moltke, Gitte Sommer Harboe, Emilie Damløv Thorsen, Line Katrine Harder Clemmensen, Jane Lindschou, Janus Engstrøm, Christian Gluud, Hartwig Roman Siebner, Per Hove Thomsen, Katja Hybel, Frank Verhulst, William Baare, Pia Jeppesen, Jens Richardt Møllegaard Jepsen, Signe Vangkilde, Markus Harboe Olsen, Julie Hagstrøm, Kerstin Jessica Plessen

**Affiliations:** 1 Child and Adolescent Mental Health Center, Copenhagen University Hospital – Mental Health Services CPH, Copenhagen, Denmark; 2 Department of Clinical Medicine, Faculty of Health and Medical Sciences, University of Copenhagen, Copenhagen, Denmark; 3 Center for Clinical Neuropsychology, Children and Adolescents, Copenhagen University Hospital – Rigshospitalet, Copenhagen, Denmark; 4 Danish Research Centre for Magnetic Resonance, Centre for Functional and Diagnostic Imaging and Research, Copenhagen University Hospital – Amager and Hvidovre, Copenhagen, Denmark; 5 Psychiatry Region Zealand, Faelledvej 6, Slagelse, DK-4200 Denmark; 6 Applied Mathematics and Computer Science, Technical University of Denmark, Kgs Lyngby, Denmark; 7 Section of Statistics and Probability Theory, Department of Mathematical Sciences, Faculty of Science, University of Copenhagen, Copenhagen, Denmark; 8 Copenhagen Trial Unit, Centre for Clinical Intervention Research, The Capital Region, Copenhagen University Hospital – Rigshospitalet, Copenhagen, Denmark; 9 Department of Regional Health Research, The Faculty of Health Sciences, University of Southern Denmark, Odense, Denmark; 10 Department of Neurology, Copenhagen University Hospital – Bispebjerg and Frederiksberg, Copenhagen, Denmark; 11 Department of Child and Adolescent Psychiatry, Aarhus University Hospital, Psychiatry, Aarhus, Denmark; 12 Department of Clinical Medicine, Aarhus University, Health, Denmark; 13 Department of Child and Adolescent Psychiatry, Copenhagen University Hospital – Psychiatry Region Zealand, Smedegade 16, Roskilde, DK-4000 Denmark; 14 Center for Neuropsychiatric Schizophrenia Research and Center for Clinical Intervention and Neuropsychiatric Schizophrenia Research, Mental Health Center Glostrup, Copenhagen University Hospital – Rigshospitalet, Glostrup, Denmark; 15 Department of Psychology, Faculty of Social Sciences, University of Copenhagen, Copenhagen, Denmark; 16 Department of Neuroanaesthesiology, The Neuroscience Centre, Copenhagen University Hospital – Rigshospitalet, Copenhagen, Denmark; 17 Division of Child and Adolescent Psychiatry, Department of Psychiatry, Lausanne University Hospital (CHUV) and University of Lausanne, Lausanne, Switzerland

**Keywords:** Obsessive-compulsive disorder, Children, Adolescents, Cognitive behavioral therapy, Exposure and response prevention

## Abstract

Few randomized clinical trials (RCTs) have compared cognitive behavioral therapy (CBT) versus active control interventions for pediatric obsessive-compulsive disorder (OCD), and the range of investigated outcomes has been limited. We investigated benefits and harms of family-based CBT with exposure and response prevention (FCBT) versus family-based psychoeducation and relaxation training (FPRT) in pediatric OCD. This single-center RCT was investigator-initiated, independently funded, including participants with OCD aged 8–17 years with a Children’s Yale-Brown Obsessive-Compulsive Scale (CY-BOCS) entry score ≥ 16. We randomized participants 1:1 to 14 sessions of FCBT versus FPRT. Allocation was masked to assessors and statisticians. The primary outcome was CY-BOCS end-of-treatment-score (week-16) analyzed by intention-to-treat. Adverse events were reported by the Negative Effects Questionnaire (NEQ-20). One-hundred-and-thirty participants were randomized, 52.3% females; mean age 13.3 (SD = 2.9) years; mean CY-BOCS total score 25.8 (SD = 4.9); *n* = 64 to FCBT versus *n* = 66 to FPRT. Sixteen participants dropped out (four from FCBT, 12 from FPRT). The mean CY-BOCS total score at end-of-treatment was significantly lower for FCBT (15.9, SD = 8.7) versus FPRT (19.9, SD = 8.1), estimate − 3.89, 95%CI [–6.83, − 0.96), *p* = 0.01, effect size = 0.47, 95% CI [0.09, 0.85]. This difference was below our predefined minimal clinically important difference of four points. The average weekly NEQ frequency score showed no significant group differences. FCBT was associated with significantly larger symptom reduction than FPRT, but with a modest effect. FCBT and FPRT appeared comparably tolerable. A rigorous methodology enabled the counteraction of several biases. Limitations included missing self-reported data and inability of masking participants and treatment providers.

**Supplementary Information:**

The online version contains supplementary material available at 10.1007/s00787-025-02797-4.

## Trial registration number and date of registration

clinical trials.gov NCT03595098, 07-23-2018.

**Supplementary Information:**

The online version contains supplementary material available at 10.1007/s00787-025-02797-4.

## Introduction

Obsessive-compulsive disorder (OCD) affects up to 3% of children and adolescents and is characterized by persistent intrusive thoughts, urges, and images (obsessions) causing anxiety, and/or by repetitive behaviors (compulsions) performed to reduce or avoid distress. OCD is associated with reduced quality of life and significant social and functional impairment and is likely to persist into adulthood [1]. Therefore, early, effective, and tolerable interventions are essential to improve prognosis.

Guidelines recommend cognitive behavioral therapy (CBT) as first-line treatment for pediatric OCD, either as monotherapy or combined with serotonin reuptake inhibitors for more severely affected patients [2, 3]. Exposure and response prevention (ERP) is the proposed key therapeutic component of CBT for OCD involving gradual and repeated exposure to anxiety-provoking situations that trigger obsessions while preventing the compulsive response [4, 5]. The therapeutic aim is to facilitate tolerance and/or reduce anxiety and thereby break the cycle of OCD. In pediatric OCD, family-based CBT (FCBT) involving parents to support the therapeutic process is recommended [6, 7]. Three prior randomized clinical trials (RCTs) in pediatric OCD indicated stronger beneficial effects of 14 weeks of FCBT with ERP compared with active placebo psychotherapy, i.e., relaxation training with or without psychoeducation and with more or less family involvement [8–10].

Reviews and meta-analyses of RCTs substantiate the recommendation of FCBT with ERP for OCD [4, 11, 12], but also raise concerns about methodological rigor of the existing trials and call for trials including a broader range of outcomes. Beyond supporting the efficacy of CBT, our review and meta-analysis [4] stated that included trials were at high risk-of-bias, the certainty of the evidence was low, and investigated outcomes other than symptom severity, such as adverse events, quality of life, and functioning were rarely included. Moreover, few trials compared CBT with credible control interventions. Two recent meta-analyses found large effect sizes for CBT with ERP versus non-ERP psychological placebo for OCD in youths and adults [11], and CBT significantly more efficacious than relaxation training in pediatric OCD [12], while both meta-analyses simultaneously highlighted the need for trials with more robust methodology and rigorous reporting, including the need for adverse event measures. Hence, RCTs designed with low risk-of-bias, specifically addressing the broader treatment effects and tolerability are highly needed to improve the evidence base for effectiveness and safety in the treatment of pediatric OCD. Moreover, carefully designed trials aiming to elucidate the role of ERP by comparing FCBT with credible control interventions can guide future clinical practice and research [11].

The TECTO RCT thus used improved and rigorous methodology to investigate benefits and harms of FCBT with ERP in pediatric OCD applied to the manual used in one of the prior RCTs [8]. We compared FCBT versus family-based psychoeducation and relaxation training (FPRT) as the active control intervention. The main and intended difference between the two interventions is the absence of the ERP component in FPRT, as ERP is considered a key therapeutic component for treating OCD. Our null hypothesis assumed that both interventions had equally beneficial effects. Our alternative hypothesis stated that FCBT would be superior to FPRT.

## Method

### Trial design

The trial is an investigator-initiated, independently funded, single-center, parallel group, hospital based (Child and Adolescent Mental Health Center (CAMHC), Copenhagen University Hospital – Mental Health Services-CPH, Denmark), randomized superiority clinical trial with masked outcome assessment. We compared 16 weeks of FCBT versus FPRT for pediatric OCD. The protocol follows the SPIRIT recommendations [13] and was registered at clinicaltrials.gov before inclusion of the first participant.

The trial complied with our protocol, the Declaration of Helsinki [14], and regulatory requirements (supplement-1**)**. The Copenhagen Trial Unit (CTU, external and independent party) monitored the activities according to Good Clinical Practice [15] and handled the data management. The protocol was published prior to data analysis [16]. People with lived experiences were involved in the trial preparations, as the trial protocol was presented for the CAMHC parent panel on a meeting on September 11, 2017, prior to submission of the protocol to national authorities for approval. We received inputs focused on informed consent materials and procedures, the rationale for comparing the two interventions, assessment of adverse events, and how parents viewed participation in psychotherapy and in research assessments.

### Participants

#### Inclusion criteria

- OCD as primary diagnosis, meeting criteria for ICD-10 F42 [17] based on transference of item criteria from the semi-structured psychopathological interview, Kiddie-Schedule for Affective Disorders and Schizophrenia – Present and Lifetime Version (K-SADS-PL) for DSM-IV [18].
- Children’s Yale-Brown Obsessive-Compulsive Scale (CY-BOCS) [19] entry score ≥ 16 as used in prior studies [8, 20].
- Age 8–17 years (both inclusive).
- Signed informed consent by parents/legal caregivers.

#### Exclusion criteria

- Comorbid illness contraindicating trial participation:
  - pervasive developmental disorder (PDD) except Asperger’s syndrome (ICD-10 F84.0-84.4; F84.8-84.9).
  - schizophrenia/paranoid psychosis (ICD-10 F20-25; F28-29).
  - mania or bipolar disorder (ICD-10 F30; F31).
  - depressive psychotic disorders (ICD-10 F32.3; F33.3).
  - substance dependence syndrome (ICD-10 F1x.2).
- IQ < 70 as measured with age-appropriate versions of the Wechsler Intelligence Scales (WISC-V [21] or WAIS-IV [22]).
- Treatment with CBT, PRT, antidepressant, or antipsychotic medications within the last six months prior to trial entry.

Eligible participants were recruited from CAMHC (informed consent procedures, see supplement-1).

### Randomization and masking

The CTU centrally randomized participants 1:1 to FCBT versus FPRT using a computer-generated allocation sequence with varying block sizes concealed from the investigators and stratified by age (8–12 years and 13–17 years) and symptom severity (baseline CY-BOCS total score 16–23 (moderate) and 24–40 (severe to extreme)). To avoid potential ’treatment-by-therapist-confounding’, all therapists conducted both interventions, and the assignment of therapists was balanced due to the randomization. Masking of participants and therapists was not possible. Investigators responsible for assessing outcomes, analysing data, and drawing conclusions were masked to group assignment (details supplement-1).

### Procedures

At baseline before randomization, we performed a diagnostic evaluation and assessed baseline values of outcome measures. We assessed social and communicative impairment with the Social Responsiveness Scale (SRS) and social/environmental family characteristics with the Family Environment Scale (FES) (instrument references in supplement-1). Sex was determined according to participants’ social security number and gender identity by asking the youths themselves. Nationality was based on participant report. Ethnicity was not assessed as this is not routine practice to register in the Danish health care system (see supplement-1). Masked follow-up assessments took place at week-4, week-8, and week-16 (end-of-treatment). Assessors were trained clinicians (psychologists or MDs) or trained psychology students under supervision. Assessors were trained, certified, and supervised in all investigator-rated instruments.

Both interventions included 14 sessions of 75 min delivered over 16 weeks. Both manuals prescribed parents to join their child fully for five of the 14 sessions (no. 1, 2, 7, 11, 14). In the remaining sessions, the child was treated individually for 45 min, followed each time by parent-sessions for an additional 30 min with or without the child present. When parents were present, the content of the session was summarized, and a predefined parent theme was worked on.

The experimental intervention used the manualised ERP-based FCBT for OCD [23] from the Nordic OCD treatment study (NordLOTS) [20]. The manual is based on the work by March and Mulle (classic individual CBT) [24], and the work by Barrett et al. concerning structured sessions focused on changing family dynamics [25], which was incorporated in the protocol by Piacentini [8]. The FCBT key components are ERP practice, homework assignments, family involvement, and psychoeducation [20]. The family factors (parent themes) specifically targeted in FCBT, are testing and training ERP and reducing family accommodation (i.e., family members’ efforts to relieve the child’s distress by accommodating obsessive-compulsive behaviors such as facilitating patients’ rituals and avoidance). The active control intervention was manualized FPRT based on the relaxation manual by Cautela and Groden [26] and adapted by Piacentini [8]. The FPRT key components are relaxation training (muscle activation and relaxation, attention training, breathing exercises, mindfulness, and visualization techniques), homework assignments, family involvement, and psychoeducation. The family factors (parent themes) specifically targeted in FPRT are relaxation practice and parents facilitating PRT for the child. In the two treatments, the common family factors targeted are psychoeducation and expectations for therapy, parents' role, the externalizing of OCD, the families' beliefs and attitude towards OCD, addressing feelings of guilt and blame, motivation for treatment, working on problem solving and cohesion, how to differentiate OCD from other problems, relapse prevention, and future plans. For further therapy details, see supplement-1 and our protocol [16].

Therapists were clinical psychologists or child and adolescent psychiatrists, and provided treatment with both interventions. A certified FCBT supervisor and a specially trained FPRT supervisor provided bi-weekly supervision to therapists in separate sessions. Therapist fidelity and adherence to treatment manuals were evaluated by two external assessors (clinical psychologists specialized in psychotherapy) who performed all ratings using the NordLOTS Treatment Integrity Scale [20] for FCBT and a corresponding manual developed by the TECTO research team for FPRT [16] (supplement-1).

To be classified as a family-based intervention, at least one parent should participate in a minimum of three sessions (study defined). We defined child treatment completion as participation in 10 out of the 14 sessions within 18 weeks (study defined). During the trial intervention, concomitant treatment with other psychotherapy, antidepressants, or antipsychotics was not permitted. All other types of concomitant treatments were permitted, provided both intervention groups had equal access. We discontinued participants from the intervention in cases of intolerable adverse reactions, symptoms contraindicating further trial participation, meeting the exclusion criteria, or experiencing a significant worsening of clinical state (i.e. a CY-BOCS total score increase of ≥ 30% from baseline) [16]. In cases of discontinuation, we encouraged participants to complete the follow-up assessments in the trial.

### Outcomes

The primary outcome was OCD symptom severity at end-of-treatment (week-16) assessed with the semi-structured clinician-rated interview CY-BOCS on 10 items rated 0 to 4 points [19]. References for the further outcome instruments are listed in supplement-1. Secondary outcomes were participant-rated health-related quality-of-life (HRQoL) assessed with the Screening Instrument for Children and Adolescents KIDSCREEN-52 and reported by the KIDSCREEN-10 Index and participant-rated adverse events assessed with the Negative Effects Questionnaire (NEQ-32) and reported by the NEQ-20 (supplement-1).

Exploratory outcomes were: (1) parent-rated HRQoL (KIDSCREEN-10 index); (2) parent-rated adverse events (NEQ-20); (3) participant- and parent-rated Child Obsessive-Compulsive Impact Scale-Revised (COIS-R); (4) participant- and parent-rated Toronto Obsessive-Compulsive Rating Scale (TOCS); (5) clinician-rated Clinical Global Impression – Severity and Improvement (CGI-S and CGI-I); (6) clinician-rated Children’s Global Assessment Scale (C-GAS); (7) clinician-rated suicidality (K-SADS-PL items suicidal thoughts, plans, or attempts); (8) clinician-rated remission (defined in two ways at end-of-treatment: i) no longer meeting the diagnostic criteria for OCD (assessed by K-SADS-PL); ii) CY-BOCS total score < 11)); (9) treatment response (reduction from baseline on CY-BOCS total score at end-of-treatment ≥ 30%); (10) serious adverse events (SAEs) [15]; 11) parent-rated family accommodation by the Family Accommodation Scale (FAS-SR); and 12) parent-rated stress pertaining to the parenting role by the Parental Stress Scale (PSS).

All outcomes were measured at baseline and at end-of-treatment. Additionally, certain outcomes were also measured at week-4 and week-8: clinical state measures (CY-BOCS; KIDSCREEN-52/-10; COIS-R; CGI-I/CGI-S); family factors (PSS; FAS); and adverse events (NEQ-32/20). Therapy factors were measured at baseline only (confidence in treatment on a 7-point Likert scale) or at baseline and sessions 1, 8, and 14 (motivation for treatment on a 7-point Likert scale; alliance on the Therapeutic Alliance Scale for Children,–Revised (TASC-R)). The therapist assessed the participants’ compliance to therapy after sessions 2 through 14.

### Statistical analysis

A detailed statistical analysis plan was published before any analyses were carried out, including subgroup analyses and handling of missing data [27]. We used R version 4.2.1 (R Core Team, Vienna Austria) and Stata version 17 (StataCorp LLC, TX, USA) for statistical analyses. The statistical reports are included in supplement-2.

The sample size estimation was based on the primary outcome, CY-BOCS total score [27]. Using a power of 80%, a two-sided alpha of 5%, and expecting a standard deviation (SD) of 8 on the CY-BOCS total score based on reports in similar patient groups [8], the required sample size necessary to detect or reject a minimal clinical important difference (MCID) of at least 4 points on CY-BOCS total score was estimated to be 64 participants in each intervention group, a total of 128 (establishment of MCID, supplement-1). To counteract the observed risk of drop-outs and missing data [28] we obtained approval to recruit and randomize up to 148 participants in total if possible within the planned trial period. Power calculations for secondary outcomes were presented in the statistical analysis plan [27].

All continuous outcomes were analyzed using linear regression (except for measures with skewed distributions not fulfilling assumptions for regression model, then Wilcoxon rank sum test was used), dichotomous outcomes using logistic regression, and count data using the Wilcoxon rank sum test. Both logistic and linear regression models included stratification variables as fixed effects and whenever available also the baseline value. In the primary analysis, we included the intention-to-treat population, and the analysis was adjusted for the stratification variables used in the randomization. For the primary outcome, we performed a post hoc per protocol analysis including data only from participants who completed 10/14 treatment sessions and completed the week-16 assessment.

All effects, other than on the primary and the two secondary outcomes, were evaluated as hypothesis-generating only. The primary analysis was based on the week-16 assessment and corrected for stratification variables and baseline values – if available. The remaining assessments were used to monitor the progress – and for the primary and secondary outcomes to carry out a mixed-effects model including all assessments and time as fixed-effect covariates. The mixed-effects model was planned as a sensitivity analysis to include more participants in our analyses and investigate if the missing values influenced the results. To control for the risk of therapist confounding, we distributed therapists randomly and equally to deliver intervention in both arms and performed a sensitivity analysis using a mixed-effects model of the primary outcome (CY-BOCS total score) including therapist as a random factor.

Shutdowns during the COVID-19 pandemic in Denmark (03-16-2020 and onwards during the trial period in varying degrees) might have negatively affected the illness severity and HRQoL of participants [29]. Therefore, we conducted exploratory sensitivity analyses by comparing the effects of the interventions before and after the lockdown (interaction between the treatment variable and inclusion before or after the lockdown date). The participants who were included before the lockdown and had follow-up after the lockdown were excluded from this analysis.

Effect modification was assessed as the interaction between the treatment variable and the four therapy factors: (1) confidence in treatment; (2) motivation for treatment; (3) therapeutic alliance; and (4) compliance. The analyses were carried out as complete case analyses.

After analyses were completed, an evaluation of the Danish language version of the KIDSCREEN-10 index examining the criterion-related construct validity and psychometric properties was published concluding that the 10-item self-report index cannot be recommended for use in population-level or small sample studies [30]. We therefore decided to perform and present a post hoc analysis using the data from the full KIDSCREEN-52 version on which the KIDSCREEN-10 index was based.

## Results

**Fig. 1:**
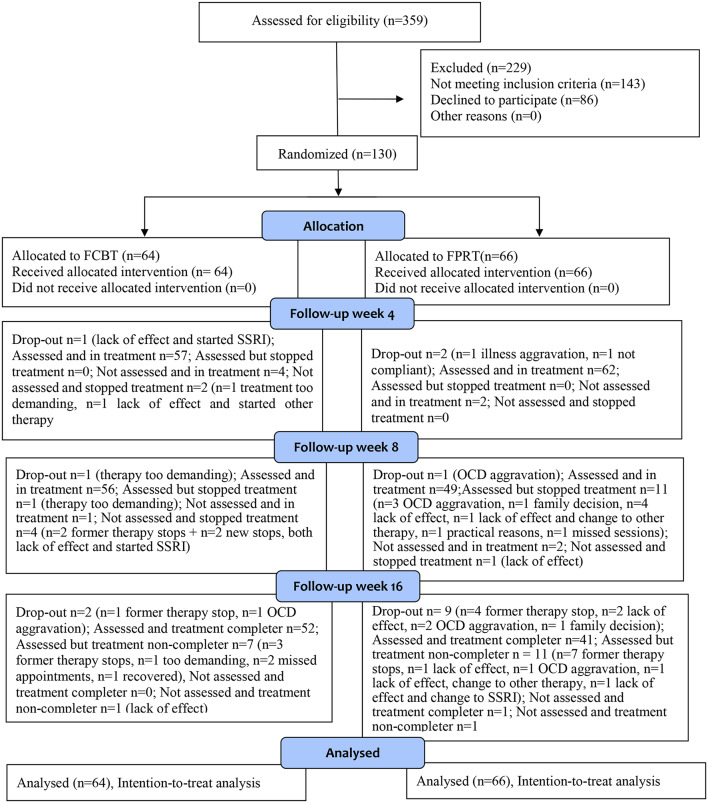
Trial profile. FCBT = family-based cognitive behavioral therapy, FPRT = family-based psychoeducation and relaxation training, SSRI = selective serotonin re-uptake inhibitors. *359 patients were assessed for eligibility of which* *n* = 143 were ineligible according to in- and exclusion criteria and *n* = *86 declined. 130 patients consented to participate*,* completed the baseline assessments*,* and were then* randomized to FCBT (*n* = 64) versus FPRT (*n* = 66). Sixteen participants (12%) *dropped out of the trial (i.e.*,* withdrew from treatments and assessments altogether)*, *n* = 4 (6%) from FCBT-ERP versus *n* = 12 (18%) from FPRT. In total *n* = *94 participants (72%)*, *n* = 52 (81%) *in FCBT versus* *n* = 42 (64%) *in FPRT*,* completed the intervention*,* and* *n* = *111 were assessed at end-of-treatment* (*n* = 59 (92%) *in FCBT versus* *n* = 52 (79%) *in FPRT). (See supplement-1 Table S4 for the number of participants included in all outcome analyses)*

Of 359 patients assessed for eligibility, 143 (40%) were ineligible according to in- and exclusion criteria, and 86 (24%) declined. The remaining 130 (36%) consented to participate within the planned trial period and were randomized to FCBT (*n* = 64) versus FPRT (*n* = 66) (Fig. 1).

Randomization of the first and last participant took place on 09-04-2018 and on 12-08-2021, respectively. Sixteen participants (12%) dropped out of the trial (i.e., withdrew from treatment and assessments altogether), four (6%) from FCBT versus 12 (18%) from FPRT. One drop-out in FPRT and one in FCBT were withdrawn from the trial due to aggravated psychopathology, while the remaining drop-outs were patient/family decisions. In total, 94 (72%) participants (52 (81%) in FCBT versus 42 (64%) in FPRT) completed the intervention according to trial definition (10 out of the 14 sessions within 18 weeks), and 111 (85%) were assessed at end-of-treatment (59 (92%) in FCBT versus 52 (79%) in FPRT). The most common reason for not completing FCBT was experiencing the treatment as too demanding (*n* = 4), while experiencing lack of effect (*n* = 11) was the most common reason for non-completion in FPRT (details on participation and non-completion Table-S1 in supplement-1). According to the trial definition, parents attended less than three sessions in two cases of FCBT and one case of FPRT, including one FCBT drop-out while the remaining 127 therapies were family-based according to the definition. Overall, parents attended a mean of 98.9% of the sessions received by the child.

Baseline characteristics of the two intervention groups of the intention-to-treat population were comparable with few exceptions (Table 1).

**Table 1:** Baseline characteristics of the intention-to-treat population (*n* = 130)

	FCBT (*n* = 64)	FPRT (*n* = 66)	Total (*n* = 130)
**Demographic characteristics**			
**Mean age**, years (SD) Range 8–17 years Strata 1 age 8–12 years, n (%) Strata 2 age 13–17 years, n (%)	13.1 (3.0) 29 (45.3) 35 (54.7)	13.4 (2.8) 31 (47.0) 35 (53.0)	13.3 (2.9) 60 (46.2) 70 (53.8)
**Female sex**, n (%)	40 (62.5)	28 (42.4)	68 (52.3)
**Male sex**, n (%)	24 (37.5)	38 (57.6)	62 (47.7)
Patient nationality* Danish, n (%) Other, n (%)	53 (82.3) 2 (3.1)	53 (80.3) 1 (1.5)	106 (81.5) 3 (2.3)
*Missing (%)*	*9 (14.1)*	*12 (18.2)*	*21 (16.2)*
Parent nationality* Danish, n (%) one Danish and one other, n (%) both other, n (%)	51 (79.7) 6 (9.4) 3 (4.7)	50 (75.8) 7 (10.6) 1 (1.5)	101 (77.7) 13 (10.0) 4 (3.1)
*Missing (%)*	*4 (6.3)*	*8 (12.1)*	*12 (9.2)*
**Parent education** ISCED 0 = Early childhood education ISCED 1 = Primary Education ISCED 2 = Lower Secondary Education ISCED 3 = Upper Secondary Education ISCED 4 = Post-sec. non-Tertiary Education ISCED 5 = Short-cycle tertiary education ISCED 6 = Bachelor’s degree or equivalent ISCED 7 = Master’s degree or equivalent ISCED 8 = Doctoral degree or equivalent	0 (0) 0 (0) 1 (1.6) 1 (1.6) 0 (0) 10 (15.6) 18 (28.1) 24 (37.5) 3 (4.7)	0 (0) 0 (0) 0 (0) 2 (3.0) 0 (0) 11 (16.7) 21 (31.8) 21 (31.8) 2 (13.6)	0 (0) 0 (0) 1 (0.8) 3 (2.3) 0 (0) 21 (16.2) 39 (30.0) 45 (34.6) 5 (3.8)
*Missing (%)*	*7 (10.9)*	*9 (13.6)*	*16 (12.3)*
**Clinical characteristics**			
**OCD symptoms**, mean total **CY-BOCS score** (SD) Scale range 0 (none) − 40 (severe) Study range: 16.0–36.0 Strata 1 CY-BOCS score 16–23 Strata 2 CY-BOCS score 24–40	25.8 (4.9) 22 (34.4) 42 (65.6)	25.8 (5.0) 22 (33.3) 44 (66.7)	25.8 (4.9) 44 (33.8) 86 (66.2)
*Missing (%)*	*0 (0)*	*0 (0)*	*0 (0)*
**Quality of life** - **patient rated**, mean **KIDSCREEN-10 T-score** (SD) High scores – better HRQoL Study range: 28.9–59.8	41.0 (6.5)	43.0 (6.1)	42.0 (6.3)
*Missing (%)*	12 (18.8)	13 (19.7)	25 (19.2)
**Quality of life** - **parent rated**, mean **KIDSCREEN-10 T-score** (SD) Higher scores – better HRQoL Study range: 27.0–56.2	39.2 (4.9)	39.1 (5.0)	39.1 (4.9)
*Missing (%)*	8 (12.5)	6 (9.1)	14 (10.8)
**OCD symptom impact** - patient rated COIS-R* score, mean (SD) Scale range 0 (none) -99 (severe) Study range: 1.0–70.0	26.5 (16.4)	29.3 (19.3)	28.0 (18.0)
*Missing (%)*	14 (29.8)	10 (20.0)	24 (24.7)
OCD symptom impact- parent rated COIS-R** score, mean (SD) Scale range 0 (none) -99 (severe) Study range: 0–88.5	29.4 (17.3)	32.2 (20.9)	30.9 (19.2)
*Missing (%)*	11 (23.4)	9 (18.0)	20 (20.6)
**Obsessive-compulsive traits - patient rated TOCS score**, median [Q1, Q3] Scale range − 63 (much less than others) - +63 (much more than others) Study range: − 56.0–53.0	17.0 [3.00, 28.0]	11.0 [5.00, 23.5]	14.0 [3.00, 25.3]
*Missing (%)*	15 (23.4)	15 (22.7)	30 (23.1)
**Obsessive-compulsive traits - parent rated TOCS score**, mean (SD) Scale range − 63 (much less than others) - +63 (much more than others) Study range: − 39.0–55.0	12.7 (18.2)	13.4 (17.2)	13.0 (17.7)
*Missing (%)*	7 (10.9)	9 (13.6)	16 (12.3)
**Global symptom severity**,** CGI-S score**, mean (SD) Scale range 1 (not at all ill) − 7 (extremely ill) Study range: 3.0–7.0	4.3 (0.9)	4.3 (0.9)	4.3 (0.9)
*Missing (%)*	*0 (0)*	*0 (0)*	*0 (0)*
**Function**, median **CGAS score** [Q1, Q3] Scale range 1 (extremely ill functioning) – 100 (extremely well-functioning) Study range: 35.0–80.0	55.0 [39.0, 80.0]	55.0 [49.0, 62.0]	55.0 [35.0, 80.0]
*Missing (%)*	*0 (0)*	*0 (0)*	*0 (0)*
**Suicidal thoughts**, K-SADS item***, n (%) Score 2 or 3 on the items suicidal ideation or acts	6 (9.4)	5 (7.6)	11 (8.5)
*Missing (%)*	*4 (6.1)*	*4 (6.3)*	*8 (6.2)*
**Social competencies**, mean **SRS total T-score** (SD) Higher scores – more severe difficulties Study range: 39.0–75.0	52.7 (8.0)	52.9 (8.1)	52.8 (8.0)
*Missing (%)*	4 (6.3)	2 (3.0)	6 (4.6)
**Intelligence**, mean **WISC-V/WAIS-IV** scores (SD) Study range: 70–132	97.8 (12.1)	99.4 (13.7)	98.6 (12.9)
*Missing (%)*	*0 (0)*	*2 (3)*	*2 (1.5)*
**Diagnostics**			
**OCD type n (%)**			
OCD primarily obsessive, ICD-10 F42.0	1 (1.6)	0 (0)	1 (0.8)
OCD primarily compulsive, ICD-10 F42.1	0 (0)	2 (3.0)	2 (1.5)
OCD mixed obsessive-compulsive, ICD-10 F42.2	63 (98.4)	64 (97.0)	127 (97.7)
OCD other/unspecified, ICD-10 F42.8/9	0 (0)	0 (0)	0 (0)
*Missing (%)*	*0 (0)*	*0 (0)*	*0 (0)*
**Comorbidity n (%)**			
Depressive disorders, ICD-10 F32	0 (0)	1 (1.5)	1 (0.8)
Anxiety disorders, ICD-10 F40-41, F93	8 (12.5)	10 (15.2)	18 (13.8)
Stress and adjustment disorders, ICD-10 F43	7 (10.9)	9 (13.6)	16 (12.3)
Eating disorders, ICD-10 F50	3 (6.3)	1 (1.5)	4 (3.1)
Sleeping disorders, ICD-10 F51	1 (1.6)	0 (0)	1 (0.8)
Personality disorders, ICD-10 F60-69	2 (3.1)	0 (0)	2 (1.5)
Specific developmental disorders, ICD-10 F81	0 (0)	1 (1.5)	1 (0.8)
Asperger’s Syndrome, ICD-10 F84.5	4 (10.9)	12 (18.2)	16 (12.3)
Other developmental disorders, unspecified, ICD-10 F88	1 (1.6)	3 (4.5)	4 (3.1)
Hyperkinetic disorders ICD-10 F90.0	7 (10.9)	8 (12.1)	15 (11.5)
Hyperkinetic conduct disorders ICD-10 F90.1	0 (0)	2 (3.0)	2 (1.5)
Conduct disorders ICD-10 F91.3	1 (1.6)	0 (0)	1 (0.8)
Attachment disorders, ICD-10 F94	0 (0)	1 (1.5)	1 (0.8)
Tic disorders, ICD-10 F95	6 (9.4)	9 (13.6)	15 (11.5)
Enuresis, ICD-10 F98.0	3 (6.3)	0 (0)	3 (2.3)
Attention deficit disorder, ICD-10 F98.8	1 (1.6)	1 (1.5)	2 (1.5)
**Number of comorbidities**,** n (%)**			
Any comorbidity	36 (56.3)	38 (57.6)	74 (56.9)
0	28 (43.8)	28 (42.4)	56 (43.1)
1	28 (43.8)	20 (30.4)	48 (36.9)
2	7 (10.9)	17 (25.8)	24 (18.5)
3	0 (0)	0 (0)	0 (0)
4	1 (1.6)	1 (1.5)	2 (1.5)
**Medicine targeting psychiatric or related symptoms n (%)**			
Melatonin	4 (6.3)	4 (6.1)	8 (6.2)
Methylphenidate	1 (1.6)	1 (1.5)	2 (1.5)
Cannabidiol	1 (1.6)	0 (0)	1 (0.8)
Valproate	1 (1.6)	0 (0)	1 (0.8)
**Family factors**			
**Parental stress**, mean **PSS score** (SD) Scale range 18 (low stress) – 90 (high stress) Study range: 19.0–65.5	36.0 (7.9)	34.4 (7.6)	35.2 (7.7)
*Missing (%)*	*12 (18.8)*	*8 (12.1)*	*20 (15.4)*
**Family accommodation parent rated FAS score**, median [Q1, Q3] Scale range 0 (none) − 76 (severe)	14.0 [0, 66.5]	16.0 [1.00, 62.00]	15.0 [0, 66.5]
*Missing (%)*	*11 (17.2)*	*8 (12.1)*	*19 (14.6)*

* We were not able to assess ethnicity and thereby not able to demonstrate diversity with respect to ethnic origin, an information that by governmental practice is not integrated in the Danish health system, but instead we used nationality which appeared mirroring the Danish background population where 85% are of Danish origin

** Self-reported from age: 11 years. Parent-reported from age: 8–17 years

*** Suicidality includes clinician-rated suicidality of K-SADS-PL suicidal thought, plans, attempts (i.e., suicidal ideation including preoccupation with thoughts of death or suicide/auditory command hallucinations; suicidal acts – judgement of the seriousness of suicidal intent; suicidal acts – judgement of medical lethality, actual medical threat to life or physical). If a patient scored 2 (subthreshold) or 3 (threshold) on any of these three items, it was defined as suicidality

*CGAS The Children’s Global Assessment Scale; CGI-S/I The Clinical Global Impression Scale - severity/improvement; COIS-R Child Obsessive Compulsive Disorder Impact Scale Revised; CY-BOCS Children’s Yale-Brown Obsessive-Compulsive Scale; FAS Family Accommodation Scale; FCBT Family-based cognitive behavioral therapy; FPRT Family-based psychoeducation relaxation therapy; ISCED International Standard Classification of Education; K-SADS-PL Kiddie-Schedule for Affective Disorders and Schizophrenia; PSS Parental Stress Scale; SRS Social Responsiveness Scale; TOCS Toronto Obsessive-Compulsive Rating Scale; WAIS-IV The Wechsler Adult Intelligence Scale; WISC-V The Wechsler Intelligence Scale for Children*.

In the total sample, mean age was 13.3 (SD = 2.9) years and 68 (52.3%) were females. No participants identified their gender as being different from their sex assigned at birth. A skewed sex distribution between groups motivated a post hoc analysis showing no significant interaction between intervention group and sex (*p* = 0.829). Almost all participants (97.7%) had mixed type OCD, and 56.9% had one to four psychiatric comorbidities. The codiagnostic distribution between groups appeared balanced. Mean intelligence quotient was close to population norms. The mean entry CY-BOCS total score was 25.8 (SD = 4.9), i.e. moderate to severe [31]. Patient/parent-rated OCD symptom impact and traits (TOCS and COIS-R) were of mild severity. Mean global functioning (C-GAS) scores indicated noticeable problems of psychological and social functioning. Mean patient/parent reported post hoc mean KIDSCREEN-52 score, was in the clinical range. Suicidal thoughts were present in 11 participants (8.5%). Global illness severity (CGI-S) was moderate to marked. Social responsiveness problems (SRS) were in the mild/moderate range [32]. Family accommodation (FAS-SR) was in the range of other clinical samples of youth in Scandinavia [33]. Parental stress (PSS) was of moderate severity. Twelve participants (3.3%) received psychotropic medication (not antidepressants or antipsychotics) at baseline for indications other than OCD. Family characteristics indicated some milder deviations from norms on measures of family environment (FES) (Table-S2 supplement-1).

**Fig. 2:**
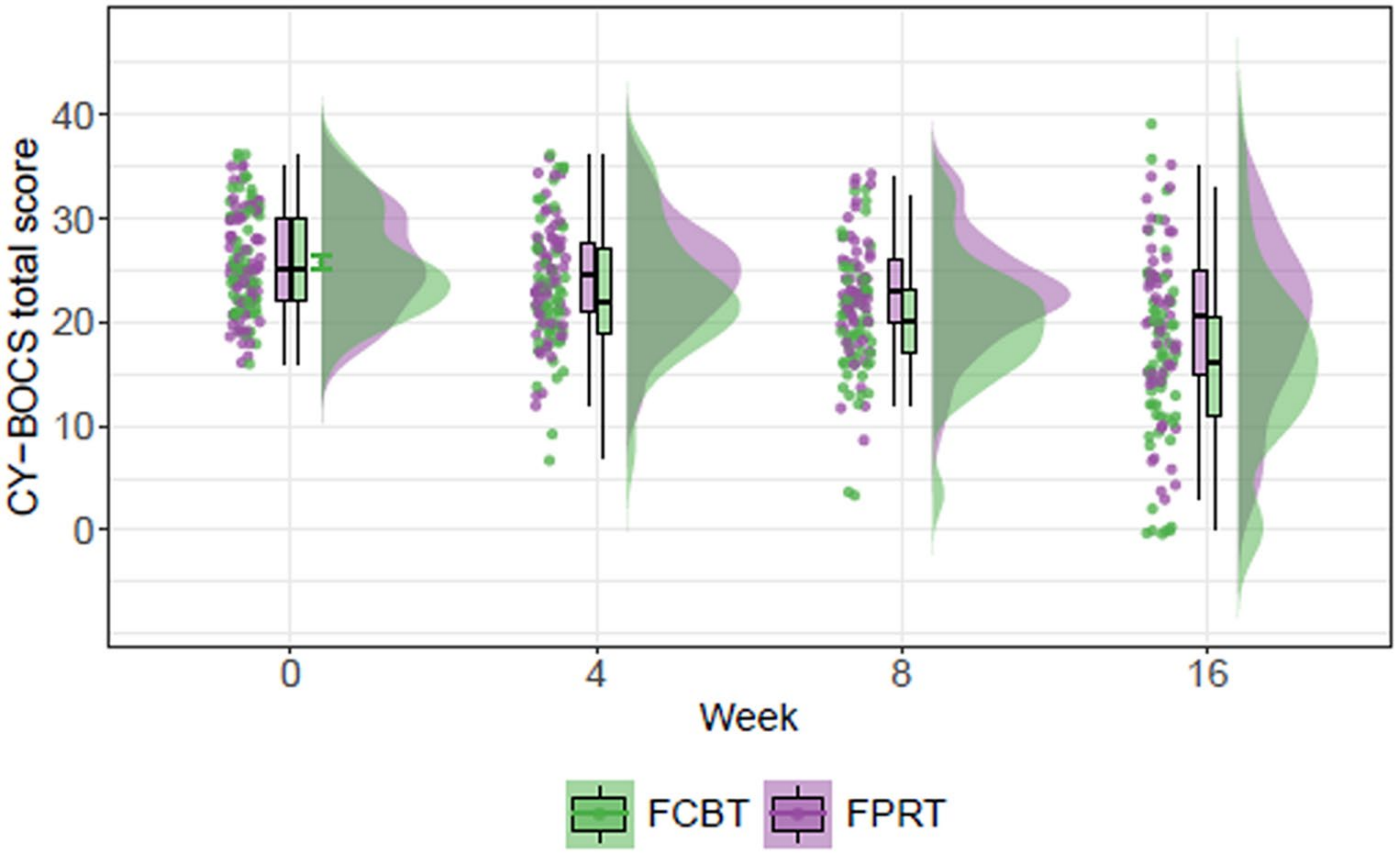
Raincloud plot of CY-BOCS total scores. CY-BOCS = Children’s Yale-Brown Obsessive-Compulsive Scale, FCBT = family-based cognitive behavioral therapy, FPRT = family-based psychoeducation and relaxation training. *Raincloud (half-density) plot of CY-BOCS total scores at week 0*,* 4*,* 8*,* and 16 showing where densities for the two intervention groups (FCBT* *n* = *64 and FPRT* *n* = *66) are clustered. The boxplot shows the five-number summary of central tendency and uncertainty (minimum*,* first quartile*,* median (vertical line)*,* third quartile*,* and maximum). The dots show the individual patient raw CY-BOCS scores*

The primary outcome measure, CY-BOCS total score at end-of-treatment, was significantly lower for FCBT (15.9 (SD = 8.68)) than for FPRT (19.9 (SD = 8.09)), mean difference − 3.89, 95% CI [–6.83, − 0.96], *p* = 0.010, effect size = 0.47, 95% CI [0.09,0.85] (Fig. 2).

The predefined mixed-effects model showed comparable patterns (mean difference − 1.74, 95% CI [–3.01, − 0.47], *p* = 0.0076). For the total sample, the mean CY-BOCS total score, decreased from 25.8 (SD = 4.9) at baseline to 17.8 (SD = 8.09) (estimate − 1.45, *p* < 0∙001) at end-of-treatment. A post hoc per protocol analysis including data restricted to participants who completed 10/14 treatment sessions and the week-16 assessment (FCBT *n* = 52 versus FPRT *n* = 41) showed a non-significant mean difference on the CY-BOCS total score 2.59, 95% CI [–5.66, − 0.49], *p* = 0.098. Furthermore, in a post hoc sensitivity analyses to exclude potential confounding associated with therapist, we excluded CY-BOCS data from five patient therapies due to participation in zero (*n* = 1) or only one session (*n* = 1) or due to two different therapists sharing the therapy provision for a patient (*n* = 3). We included therapist (*n* = 8) as a random factor and found a mean difference on CY-BOCS total score − 3.11, 95% CI [–6.00, − 0.22], *p* = 0.038.

**Fig. 3:**
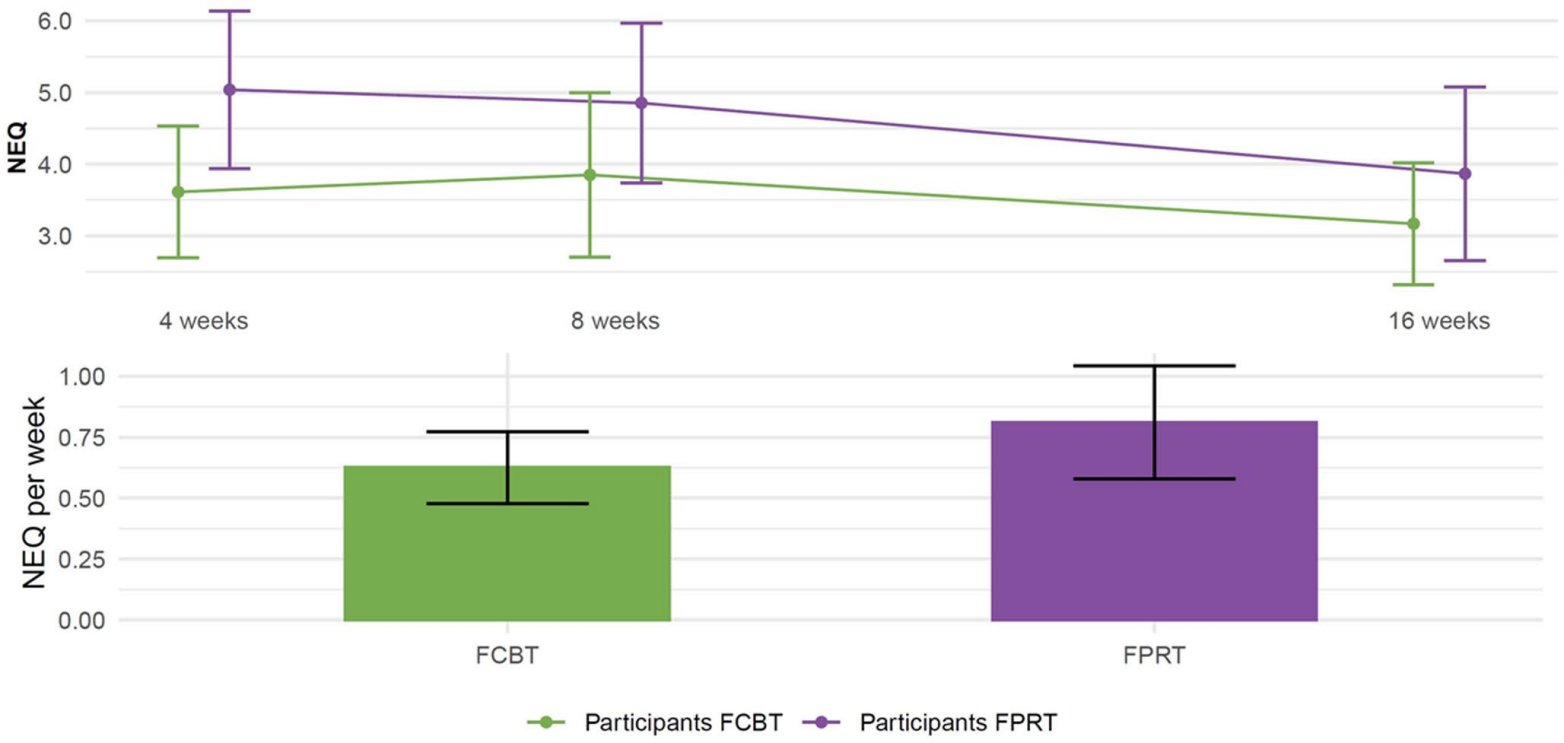
Negative effects by participant-rated Negative Effects Questionnaire (NEQ-20). FCBT = Family-based cognitive behavioral therapy; FPRT = Family-based psychoeducation relaxation therapy; NEQ-20 = Negative Effects Questionnaire

The secondary outcome, mean number of participant-reported negative effects (NEQ-20) for FCBT versus FPRT in the period week 1–4, week 5–8, and week 9–16 were similar, and the mean weekly NEQ-20 frequency score for the whole period was not significantly different for FCBT = 0.625 (SD = 0.434) versus FPRT = 0.810 (0.595), estimate − 0.20, 95% CI [–0.44,–0.05], *p* = 0.114. Exploratory parent-reported NEQ-20 showed a parallel and non-differential development over time as the participant-rated scores (Fig. 3; Table-S3 supplement-1).

The secondary outcome, the post hoc analysis of participant-rated KIDSCREEN-52 showed slightly increasing T-scores on all 10 dimensions with no differential group effects at week-16 (Table-S4, Table-S5, Figure-S1, Figure-S2 supplement-1 show KIDSCREEN-10 and KIDSCREEN-52 results).

**Fig. 4:**
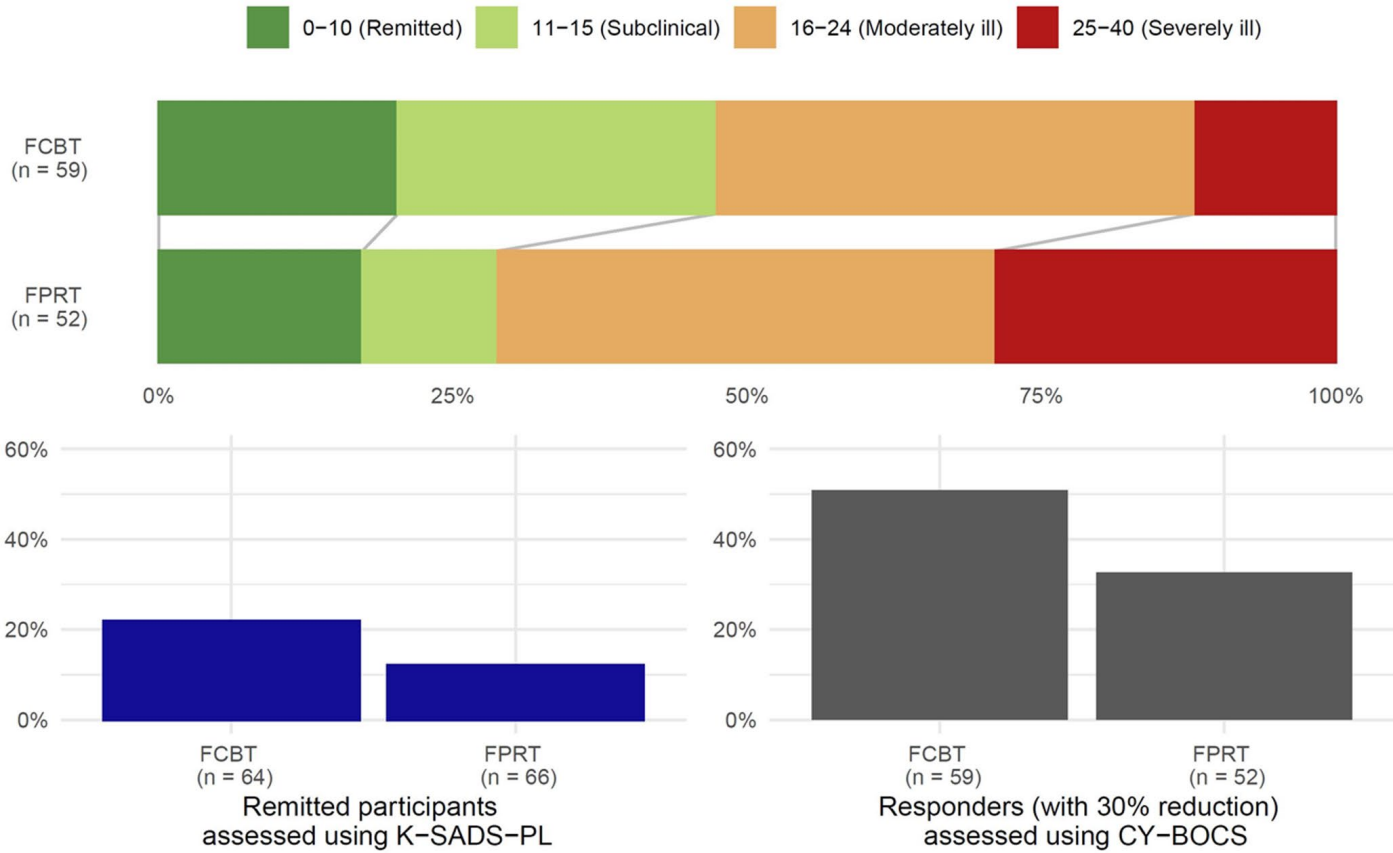
Response and remission rates. FCBT = family-based cognitive behavioral therapy, FPRT = family-based psychoeducation-relaxation training, K-SADS-PL = Kiddie-Schedule for Affective Disorders and Schizophrenia Present and Lifetime, CY-BOCS = Children’s Yale-Brown Obsessive-Compulsive Scale. *Top panel: Distribution of clinical severity categories based on CY-BOCS total score intervals*

The course of all outcomes of psychopathology, family function, and level of functioning are shown in Figure-S1 and table-S4, supplement-1. Briefly, parent-reported KIDSCREEN-52 scores increased on all 10 dimensions (except for physical well-being in the FCBT group) with no differential group effects at week-16. Participant-/parent-rated OCD symptom impact (COIS-R) and participant-/parent-rated obsessive-compulsive traits (TOCS) decreased with no significant group differences. Mean global symptom severity (CGI-S) improved (decreased) for the whole sample from 4.29 (SD = 0.86) to 3.60 (SD = 1.34) and significantly more for FCBT than FPRT, estimate − 0.54, 95% CI [–1.02, − 0.06], *p* = 0.026. The median CGI-I score at week-16 was significantly more favorable 2.0 [Q1 = 2.0, Q3 = 2.0] (much improved) for FCBT than for FPRT 3.0 [Q1 = 2.0, Q3 = 3.0] (minimally improved), W = 1711, *p* = 0.045. Global functioning (C-GAS) and parental stress (PSS) improved for the whole sample with no significant group differences. Median family accommodation (FAS-SR) improved (scores decreased) for the whole sample and significantly more with FCBT than with FPRT, W = 1126, *p* = 0.015. At week-16, five participants (7.8%) in FCBT and two (3.0%) in FPRT experienced suicidal thoughts, which was not significantly different. Two (3.1%) SAEs were registered in FCBT (one arm fracture, one aggravation of OCD) and three (4.5%) in FPRT (one somatic complication related to OCD, one aggravation of OCD, and one arm fracture) with no significant difference. Response rate (Fig. 4) was significantly higher in FCBT (*n* = 30/64 (46.9%)) versus FPRT (*n* = 17/66 (25.8%)), RR = 1.58, 95% CI [1.03, 2.71], *p* = 0.042. Remission rates (Fig. 4) were higher but not significantly in FCBT (no OCD diagnosis *n* = 14/64 (21.9%); CY-BOCS < 11 *n* = 12/64 (18.8%)) versus FPRT (no OCD diagnosis *n* = 8/66 (12.1%); CY-BOCS < 11 *n* = 9/66 (13.6%)).

*FCBT: remitted 18.8*% *(CYBOCS total score* < *11)*,* subclinical 25.0*%,* moderately ill 27.5%*,* severely ill 10.9*%. *FPRT: remitted 13.6*% *(CYBOCS total score < 11)*,* subclinical 9.1%*,* moderately ill 33.3*%,* severely ill 22.7*%. *Lower left panel: Remission rate (patient no longer fulfilling ICD-10 diagnostic criteria for OCD according to K-SADS-PL) was higher in FCBT* *n* = *14 (21.9*%) *versus FPRT* *n* = *8 (12.1*%*)*,* but not significantly. Lower right panel: Response rate (defined as at least 30*% *reduction on CY-BOCS total score from baseline to week-16) was significantly higher in FCBT*, *n* = *30 (46.9*%) *versus in FPRT* *n* = *17 (25.8*%*)*, *p* = *0.042.*

Exploratory sensitivity analysis showed that COVID-19 before/after shutdown (estimate 1.08, 95% CI [–5.21, 7.38], *p* = 0.734) was not a significant moderator of CY-BOCS total scores at week-16. Moreover, when assessing effect modification as the interaction between the treatment variable and the four therapy factors, none were significant moderators of CY-BOCS total scores at week-16, Table-S6 supplement-1. The rating of therapist fidelity to manuals (4-point Likert scale ranging from 1 (very poor competence) to 4 (very good competence)) based on video recordings of 40 therapy sessions (20 FCBT and 20 FPRT) showed an overall good to very good manual compliance (mean score range 3.40–3.65), competence (mean score range 3.50–3.55), and differentiation (mean score range 3.65–3.80), and all measures showed no significant group differences for FCBT versus FPRT in ratings (Table-S7 supplement-1).

## Discussion

Within a rigorous design, the TECTO trial investigated the benefits and harms of FCBT versus FPRT in youths with OCD aged 8–17 years. We found a small and significant advantage of FCBT over FPRT in terms of the primary outcome, i.e. OCD symptom alleviation, without FCBT posing an increased risk of negative treatment effects. We add evidence to prior results of trials by contributing with data from the largest RCT sample of youth in the age range 8–17 years comparing CBT to a credible active control intervention that by design allow for disentangling general and specific therapy effects, and specifically add evidence to ERP as an important ingredient in the treatment of OCD. This includes the focus of FCBT on reducing family accommodation thereby supporting the ERP processes, which may help maintain treatment effects [34]. The effect of FCBT and FPRT were non-differential on HRQoL. Our exploratory analyses indicated better outcomes for FCBT versus FPRT in terms of clinical global impression, response rate, and family accommodation. Finally, more participants in the FCBT arm completed treatment.

Our results align with the three prior RCTs indicating stronger beneficial effects of 14 weeks of FCBT compared with active placebo psychotherapy (family based relaxation therapy with (FPRT) or without psychoeducation (FRT) or relaxation therapy with psychoeducation and less family involvement (PRT)) in youths with OCD [8–10]. Piacentini et al. [8] investigated a smaller sample (FCBT *n* = 49; PRT *n* = 22) in the same age range as ours and also found significantly higher response rates (CGI-I = 1 or 2 (very much or much improved)) for FCBT = 57.1% (*n* = 49) compared with PRT = 27.3% (*n* = 22) and a faster decline in OCD symptom severity with FCBT. However, mean symptom reduction (CY-BOCS) and remission rates (FCBT 42.5% versus PRT 17.6%) did not show significant differential effects. Compared to Piacentini and colleagues’ results, our response rates (≥ 30% reduction on CY-BOCS total score) were lower for FCBT (46.9%) and comparable for FPRT (26.8%) but still showed significant group differences. Our effect size (based on CY-BOCS) was slightly higher (0.47) compared to Piacentini’s (0.40). The two RCTs by Freeman and colleagues included younger children aged 5–8 years. In the first trial (FCBT *n* = 22; FRT *n* = 20), the intention-to-treat-analyses showed a non-significant differential effect favoring FCBT, while complete case analysis showed a larger and significant effect on symptom alleviation for FCBT versus FRT [10]. The second and larger trial (FCBT *n* = 63; FRT *n* = 64) showed superiority of FCBT for responder status (CGI = 1 or 2: 72% versus 41%) and for CY-BOCS total score improvement, and a higher effect size (0.84) than Piacentinis and ours [9].

The response to ERP, i.e., the decline in CY-BOCS scores in FCBT shown in our trial (from 25.8 at baseline to 15.9 at week 16, i.e. – 9.9) was in the same range as the other RCTs testing manualized FCBT versus relaxation therapies, ranging from − 8.5 [10] to − 11.4 [8] and − 13.0 [9]. It was also similar when we broadened the comparison to more RCTs of CBT in pediatric OCD compared to a variety of control interventions where reductions on CY-BOCS total score ranged from 9.0 to 15.3 and a calculated weighted mean reduction was 11.6 points on total CY-BOCS score based on the data from 19 studies and 533 participants receiving individual (including family-based) in-person (including one study with added pill placebo) therapies in the meta-analysis by Cervin et al. [12].

Our trial thus expands previous findings of a better outcome for FCBT compared with FPRT in a large, unmedicated, and clinically more representative sample (including comorbid suicidality and Asperger’s syndrome). The Piacentini and Freeman RCTs excluded patients with suicidality and any PDD, and Freemans’ RCTs allowed medication with antidepressants. A recent RCT (a 12-week trial designed for studies of brain activation and connectivity associated with treatment response) allowing for antidepressants, but excluding suicidal patients found significantly larger OCD symptom decrease for adolescent patients treated with CBT-ERP (*n* = 27) versus stress management therapy (*n* = 27) [35, 36].

Our secondary outcome, participant-rated HRQoL showed no group differences, which mirrors the Freeman findings [9]. Assessing AE in trials like TECTO is rare. Piacentini did not assess AEs of treatment, whereas Freeman only assessed SAEs. As an important novel finding and despite the potentially distressing ERP component of FCBT [37] there were no group differences in the number of AE, thereby indicating comparable tolerability. In addition, the rate of SAEs was low in both groups and suicidality declined from 8.5% at baseline to 5.4% at end-of-treatment with no group differences. This is reassuring as suicidality appears common in pediatric OCD (one small study reported 7/54 (13%)) [38] and even more in adults (16–63% suicidal ideation and 25% prior suicide attempt) [39]. Attrition rates might also indicate negative effects and attrition rates of 10% for CBT with ERP in youths has been reported [40]. We found attrition higher in FPRT *n* = 24 (36%, primarily due to lack of effect) than in FCBT *n* = 12 (19%, mostly due to finding the therapy too demanding).

Our exploratory analyses are only hypothesis generating. Nevertheless, several results appeared to substantiate the advantage of FCBT over FPRT, i.e. on clinical global impression severity and improvement, family accommodation, and response rates. However, analysis of other exploratory outcomes did not indicate significant superiority of either intervention. Since the CGI-S/I measures cover aspects of functioning, our results may indicate superiority of FCBT on functional improvement. Yet, the specific measure of psychosocial functioning only showed insignificant advantages of FCBT (C-GAS) or no difference of impact (COIS-R). Piacentini found superior function improvement for FCBT compared with FPRT, but only on child-reported, not on parent-reported COIS-R. Moreover, our exploratory analyses found no significant moderators of treatment effects on CY-BOCS, including the COVID-19 shutdown and the four therapy factors (confidence in treatment, motivation, alliance, and compliance).

The strengths of the TECTO trial include our prior registrations and publications of protocol and statistical analysis plan [16, 27], the unbiased randomization procedures, the masked outcome assessment, and a balanced design with fully manualized and equally dosed, supervised, and fidelity-rated interventions. Notably, external experts showed high and equal quality of therapist fidelity with the two interventions. Moreover, we investigated a large clinically representative sample of youth with OCD compared to prior trials by including participants with suicidality and a broad spectrum of comorbidities. We also systematically assessed a broad range of outcomes including harms, which are generally not systematically monitored or reported in psychotherapy trials [4, 41], despite recent recommendations in the Reporting Randomized Trials of Social and Psychological Interventions: the CONSORT-SPI-2018 Extension [42]. In addition, TECTO is the first RCT to assess remission for pediatric OCD through a diagnostic interview at end-of-treatment, which converged with our results from conventional definitions (CY-BOCS total score < 11).

We chose the exploratory outcomes to supplement the primary and secondary outcomes with parent perspectives on HR-QOL and negative effects, OCD-specific/general function (COIS-R, C-GAS), multidimensional OCD traits (TOCS), global clinical change (CGI-S/I), suicidality (K-SADS-PL items), remission, response, SAEs, and family burden (FAS-SR and PSS). This enables a multidimensional impression of outcomes with evaluations from both professionals, patients, and parents on different aspects of treatment effects and allows for comparison to other trials reports, while respecting that these results may only generate hypotheses.

One trial limitation is the inherent lack of participant and therapist masking in face-to-face psychotherapy trials, which may challenge an unbiased interpretation of effect estimates. However, this limitation is mitigated by blinded assessments, by comparing interventions that both contain active therapeutic elements, and by our finding of similar levels of youth expectations, therapeutic alliance and compliance, motivation for treatment, and confidence in treatment. Another limitation is missing data from follow-up assessments, especially from participant-reported questionnaires. Nevertheless, missing data were more limited on outcomes assessed by investigators. Furthermore, only 81% in FCBT and 64% in FPRT completed the intervention. In our post hoc analysis restricted to treatment-completers FCBT still appeared advantageous compared with FPRT, albeit not reaching statistical significance. As the main reason for participants not completing FPRT was lack of effect, the FPRT-completers may be those who experienced the greatest benefits thereby diminishing the difference in effect between FCBT and FPRT. Moreover, although the NEQ is validated for assessing adverse events in psychotherapy trials [43], it has not yet been validated in youth. Furthermore, although the KIDSCREEN-10 is recommended among standard outcome measures for pediatric OCD [44], a recent call for caution questions the psychometric properties of the KIDSCREEN-10, at least in a Danish context [30]. Moreover, due to limited resources, only 40 therapy sessions, and solely session 8, were fidelity rated despite our original ambition of rating 15% of all FCBT and FPRT sessions, distributed evenly across the 14 treatment sessions. Finally, despite our aim to include a clinically representative sample compared with other RCTs, the fact that we excluded patients with PDD (except for Asperger’s syndrome) with which comorbidity rates with OCD are high [45] limits the generalizability of results and thereby our ability to address a gap in the literature on the efficacy of therapeutic interventions for comorbid OCD and the broader PDD spectrum.

Our trial aimed to meet the methodological demands raised in several systematic reviews [11, 12], including our own review and meta-analysis [4], which initiated some debate [46–48]. The question is whether the differential effect on the primary outcome of 3.89, which is just below our pre-specified MCID of 4.00 points on CY-BOCS total score, is large enough to support FCBT with ERP as a first-line treatment for youths with OCD. Retrospectively, it is not possible to infer what results would have emerged had there been fewer or no drop-outs or missing data. Nevertheless, our overall results buttress the efficacy and indicates tolerability of FCBT while also showing that FPRT may have a beneficial effect. However, we had no inactive comparison intervention to essentially prove the effects of any of the interventions per se.

Overall, our results indicate that FCBT is a safe and beneficial treatment for youths with OCD. Our main message is to confirm that ERP appears an effective ingredient in the psychotherapy of pediatric OCD. Importantly, clinicians should be aware that mean symptom reduction during psychotherapy for OCD is limited to around 40%, and that approximately 50% of patients do not or only partially respond to FCBT and therefore need continued and/or adjunctive treatments. Future research should further investigate long-term maintenance effects of FCBT for OCD and guide personalized treatment choices, for example by studying the underlying mechanisms of change in psychotherapy for OCD to specify treatment targets, and identify predictors, moderators, and mediators of treatment effects. Prior studies have highlighted several mechanisms (e.g. family accommodation, family conflict, parental blame, and poor family cohesion) through which family factors influence therapy effectiveness [6, 34, 49, 50]. However, the level of parental involvement needed to enhance outcomes is not entirely clear [51]. Nevertheless, for some children who are unable to participate in psychotherapy, a completely parent-based intervention like Supportive Parenting for Anxious Childhood Emotions (SPACE) [52] might prove effective in future trials. Current work in the TECTO study group aim to address potential neurobiological, neurocognitive, and neuroendocrine biomarkers as well as mediators of treatment response [53–55].

## Electronic supplementary material

Below is the link to the electronic supplementary material.

Supplementary Material 1

Supplementary Material 2

Supplementary Material 3

## Data Availability

After these results and the planned substudies have been published we will make a deidentified dataset used for the present and substudy analyses publicly available on the EU ZENODO database https://zenodo.org/communities/eu/.
