# Supplementary material for: Family-based cognitive behavioral therapy versus family-based psychoeducation and relaxation training for obsessive-compulsive disorder in children and adolescents: a randomized clinical trial (TECTO)
Source: Eur Child Adolesc Psychiatry. 2025 Jul 31;34(12):3955–71. doi: 10.1007/s00787-025-02797-4 (PMC12743107; doi:10.1007/s00787-025-02797-4)
Supplement: Supplementary file 1 — Supplementary Material 1 [file 787_2025_2797_MOESM1_ESM.pdf]

Details on compliance with regulatory requirements:

The trial was conducted in compliance with the study protocol, the Declaration of Helsinki and the applicable regulatory requirements (The Ethics Committee of Capital Region of Denmark approval number: H-18010607 (obtained by CAMHC), and The Knowledge Centre on Data Protection Compliance in The Capital Region of Denmark: VD-2018-263, I-Suite no.: 6502). We acted in accordance with the Danish personal and health data regulations, when collecting information from participants' medical records (The Danish Act on Processing of Personal Data, and Danish Health Act, Section 43, Subsection 1). Recruitment of participants started after regulatory approvals were obtained.

Details on informed consent procedures:

Patients received verbal (all ages) and written (adolescents aged at least 15 years and all parents/legal guardians) information about the trial and were informed of their rights to withdraw from the trial at any point without it affecting future treatment. Parents (or legal guardians) signed informed consent concerning participation of their child. If a participant turned 18 years during the study period, the participant confirmed his/her/their own participation by giving own informed consent. Each parent furthermore received their own participant information (verbal and written) and gave informed consent regarding parental participation. Trial participants were compensated with a DKK 250 (approximately GBP 30) gift card at baseline and end-of-treatment test days.

Details on masking procedures:

Outcome assessors, data managers, statisticians, and conclusion drawers were blinded. Due to the nature of the intervention, therapists were unblinded and families were only partially blinded, i.e., the name and the detailed content of the assigned intervention was not fully disclosed to participants and their parents. Before the follow-up assessments, unblinded trial personnel instructed the child/families to avoid giving any information concerning the therapy to the blinded outcome assessor. To further ensure blinding, the statistical analyses were conducted with the intervention groups coded as 'Intervention A' and 'Intervention B'. We wrote two abstracts while the blinding was intact: one assuming the experimental intervention group was A and the control intervention group was B, and one assuming the opposite. Afterwards, the identities of intervention groups A and B were revealed. In line with the CONSORT 2010 guidelines, the success of masking was not tested.

Details on common components of the interventions:

The key common components in FCBT and FCBT are homework assignments, family involvement, and psychoeducation. Additional common therapeutic elements in both interventions include externalising OCD, setting an agenda at each session, monitoring and ranking symptoms, providing a treatment rationale, using positive reinforcement, and building a collaborative alliance between participant and therapist. The treatments were manualised, yet also individualised regarding parent involvement and adjustment to child age. Both manuals prescribed parents to join their child fully for five of the 14 sessions. In the remaining sessions, the child was treated individually

for 45 minutes, followed by parent-sessions for an additional 30 minutes with or without the child present. When parents were present, the content of the session was summarised, and a predefined parent theme was worked on. In the individual part of the CBT session, classical CBT with ERP took place with the child (status since last session, updating symptom hierarchy, exposure training and homework for next week). In the individual part of the PRT session, a parallel set of activities were carried through (status since last session, updating symptom hierarchy, training of muscular and verbally cued relaxation techniques, and homework for next week).

Details on methods for fidelity and adherence ratings:

Treatment fidelity and adherence to the manuals was assessed using the NordLOTS Treatment Integrity Scale for FCBT and a corresponding manual developed by the TECTO research team for FPRT. Each adherence/fidelity manual included ratings of (a) adherence to the treatment manual (assessment of homework, exposure and response prevention/relaxation training, and parental involvement); (b) therapist competence (ability to explain and implement the treatment manual in session); (c) relational skills; and (d) treatment differentiation. Ratings were scored on a 4-point Likert scale ranging from 1 (“very poor competence”) to 4 (“very good competence”). Two external assessors (clinical psychologists with specialist training in psychotherapy) performed all ratings.

According to our protocol, we aimed to investigate fidelity to the manuals with approximately 15% of all FCBT sessions and FPRT sessions, distributed evenly across the 14 treatment sessions, and randomly selected. However, due to limited resources, we changed our aim to investigate three video-recorded sessions per therapist per manual to ensure representativeness, i.e., 48 therapy sessions. Session number 8 (out of 14) was chosen for rating. If not available, sessions 6 or 9 were used instead in that order of preference. We prioritized these sessions as they were representative of most sessions by not having parents present throughout the entire session and by including an exercise with either ERP (in the FCBT arm) or relaxation (in the FPRT arm).

Details on ethnicity assessments:

We were not able to assess ethnicity and thereby not able to demonstrate diversity with respect to ethnic origin, an information that by governmental practice is not integrated in the Danish health system, but instead we used nationality which appeared mirroring the Danish background population where 85% are of Danish origin.

Reference list for measurement instruments:

Social Responsiveness Scale

1. Constantino JN (2013) Social Responsiveness Scale. In: Volkmar FR (ed) Encyclopedia of Autism Spectrum Disorders. Springer, New York, NY, pp 2919–2929

The Family Environment Scale

2. Boyd CP, Gullone E, Needleman GL, Burt T (1997) The Family Environment Scale: reliability and normative data for an adolescent sample. Fam Process 36:369–373. https://doi.org/10.1111/j.1545-5300.1997.00369.x

KIDSCREEN-52

3. Ravens-Sieberer U, Gosch A, Rajmil L, et al (2005) KIDSCREEN-52 quality-of-life measure for children and adolescents. Expert Rev Pharmacoecon Outcomes Res 5:353–364. https://doi.org/10.1586/14737167.5.3.353

KIDSCREEN-10

4. Ravens-Sieberer U, Erhart M, Rajmil L, et al (2010) Reliability, construct and criterion validity of the KIDSCREEN-10 score: a short measure for children and adolescents' well-being and health-related quality of life. Qual Life Res 19:1487–1500. https://doi.org/10.1007/s11136-010-9706-5

The Negative Effects Questionnaire

5. Rozental A, Kottorp A, Boettcher J, et al (2016) Negative Effects of Psychological Treatments: An Exploratory Factor Analysis of the Negative Effects Questionnaire for Monitoring and Reporting Adverse and Unwanted Events. PloS One 11:e0157503. https://doi.org/10.1371/journal.pone.0157503

The Child Obsessive-Compulsive Impact Scale-Revised

6. Piacentini J, Peris TS, Bergman RL, et al (2007) Functional impairment in childhood OCD: development and psychometrics properties of the Child Obsessive-Compulsive Impact Scale-Revised (COIS-R). J Clin Child Adolesc Psychol Off J Soc Clin Child Adolesc Psychol Am Psychol Assoc Div 53 36:645–653. https://doi.org/10.1080/15374410701662790

The Toronto Obsessive-Compulsive Scale:

7. Park LS, Burton CL, Dupuis A, et al (2016) The Toronto Obsessive-Compulsive Scale: Psychometrics of a Dimensional Measure of Obsessive-Compulsive Traits. J Am Acad Child Adolesc Psychiatry 55:310-318.e4. https://doi.org/10.1016/j.jaac.2016.01.008

The clinical global impressions scale

8. Busner J, Targum SD (2007) The clinical global impressions scale: applying a research tool in clinical practice. Psychiatry Edgmont Pa Townsh 4:28–37

A children's global assessment scale

9. Shaffer D, Gould MS, Brasic J, et al (1983) A children's global assessment scale (CGAS). Arch Gen Psychiatry 40:1228–1231. https://doi.org/10.1001/archpsyc.1983.01790100074010

The Family Accommodation Scale

10. Pinto A, Van Noppen B, Calvocoressi L (2013) Development and preliminary psychometric evaluation of a self-rated version of the Family Accommodation Scale for Obsessive-Compulsive Disorder. J Obsessive-Compuls Relat Disord 2:457–465. https://doi.org/10.1016/j.jocrd.2012.06.001

The Parental Stress Scale

11. Berry JO, Jones WH (1995) The Parental Stress Scale: Initial Psychometric Evidence. J Soc Pers Relatsh 12:463–472. https://doi.org/10.1177/0265407595123009

Therapeutic Alliance Scales for Children--Revised

12. Creed TA, Kendall PC (2005) Therapeutic Alliance Scales for Children--Revised (TASC-R) [Database record]. APA PsycTests. https://doi.org/10.1037/t21462-000

Items of The KIDSCREEN-10 index

https://www.kidscreen.org/english/questionnaires/kidscreen-10-index/

© The KIDSCREEN Group, 2004; EC Grant Number: QLG-CT-2000- 00751

The KIDSCREEN-10 index was developed from the KIDSCREEN-27 and operationalizes general health-related quality of life in a single scale.

With only 10 items, the KIDSCREEN-10 index is the shortest version of the KIDSCREEN questionnaires and measures general health-related quality of life (HRQoL). This index can also be calculated if the KIDSCREEN-52 or KIDSCREEN-27 were used. It then provides a useful global measure of health-related quality of life in addition to the individual aspects.

Responders rate each item on a 5 point likert scale: not at all/never, slightly/seldom, moderately/quite often, very/very often, extremely/always

In the present paper of the TECTO RCT we used the KIDSCREEN-52 version but chose to report the KIDSCREEN-10 index to provide a global measure of HRQoL. Post-hoc we also analysed and reported results from the KIDSCREEN-52 for the reason explained in the main text.

For correct scoring and comparison with norm data, additional information can be found at https://www.kidscreen.org/english/analysis/

KIDSCREEN-10 Index, Health Questionnaire for Children and Young People, Child and Adolescent Version, 8 to 18 Years

Hello,

How are you? How do you feel? This is what we would like you to tell us.

Please read every question carefully. What answer comes to your mind first? Choose the box that fits your answer best and cross it.

Remember: This is not a test so there are no wrong answers. It is important that you answer all the questions and also that we can see your marks clearly. When you think of your answer please try to remember the last week.

You do not have to show your answers to anybody. Also, nobody who knows you will look at your questionnaire once you have finished it.

About Your Health
Thinking about the last week...

- *Have you felt fit and well?*
- *Have you felt full of energy?*
- *Have you felt sad?*
- *Have you felt lonely?*
- *Have you had enough time for yourself?*
- *Have you been able to do the things that you want to do in your free time?*
- *Have your parent(s) treated you fairly?*
- *Have you had fun with your friends?*
- *Have you got on well at school?*
- *Have you been able to pay attention?*

not at all/never, slightly/seldom, moderately/quite often, very/very often, extremely/always
In general, how would you say your health is? Excellent, very good, good, fair, poor

KIDSCREEN-10 Index Health Questionnaire for Children and Young People, Parent Version

Dear Parents,

How is your child? How does she/he feel? This is what we would like to know from you.

Please answer the following questions to the best of your knowledge, ensuring that the answers you give reflect the perspective of your child. Please try to remember your child's experiences over the last week...

About Your Child's Health

1. *Has your child felt fit and well?*
2. *Has your child felt full of energy?*
3. *Has your child felt sad?*
4. *Has your child felt lonely?*
5. *Has your child had enough time for him/herself?*
6. *Has your child been able to do the things that he/she wants to do in his/her free time?*
7. *Has your child felt that his/her parent(s) treated him/her fairly?*
8. *Has your child had fun with his/her friends?*
9. *Has your child got on well at school?*
10. *Has your child been able to pay attention?*

not at all/never, slightly/seldom, moderately/quite often, very/very often, extremely/always

In general, how would your child rate her/his health? Excellent, very good, good, fair, poor

Items of The Negative Effects Questionnaire Version 20 Items (NEQ-20)

www.neqscale.com

Negative Effects Questionnaire (NEQ) Version 20 Items (2017-10-25), Alexander Rozental & Per Carlbring, Stockholm University.

The self-report measure consists of three parts. First, respondents endorse specific items in case they have occurred or not during treatment, yes/no. Second, the respondents rate how negatively the negative effect was on a Likert-scale, ranging from ”Not at all” to ”Extremely”, 0-4 (”0” being minimum and ”4” being maximum). Third, the respondents attribute the negative effect to ”The treatment I received” (1) or ”Other circumstances” (0). There is currently no consensus on how to interpret scores from a self-report measure on negative effects of psychological treatments. However, summing up the frequencies and providing information on means and standard deviations can help with comparisons between different samples.

In the present paper of the TECTO RCT, the items were summed up for each participant at each time point in order to get a frequency measure of the number of negative effects the respondents have experienced. We did not evaluate the negative impact or the attribution to treatment.

Negative incidents and effects of psychological treatment incidents and effects that are viewed as both positive and negative can occur during treatment. We now want you to think about what happened during the period when you received your treatment, and that you consider if anything you experienced was negative or unwanted. Read through the following statements and mark whether you experienced any of these incidents or effects. If you answer yes, please indicate the severity of your experience (how negative the experience was for you), and whether you believe your experience was caused by the treatment you received or other circumstances that occurred during the same period as your treatment.

1. I had more problems with my sleep

2. I felt like I was under more stress

3. I experienced more anxiety

4. I felt more worried

5. I experienced more hopelessness

6. I experienced more unpleasant feelings

7. I felt that the issue I was looking for help with got worse

8. Unpleasant memories resurfaced

9. I became afraid that other people would find out about my treatment

10. I got thoughts that it would be better if I did not exist anymore and that I should take my own life

11. I started feeling ashamed in front of other people because I was having treatment

12. I stopped thinking that things could get better

13. I started thinking that the issue I was seeking help for could not be made any better

14. I think that I have developed a dependency on my treatment

15. I did not always understand my treatment

16. I did not always understand my therapist

17. I did not have confidence in my treatment

18. I felt that the treatment did not produce any results

19. I felt that my expectations for the therapist were not fulfilled

20. I felt that the treatment was not motivating

Details on establishment of minimal clinically important difference (MCID):

Establishing a MCID, i.e., the smallest change in a treatment outcome that an individual patient would identify as important and which would indicate a change in the patient's management, is complex. Especially when there are only few RCTs comparing the exact same interventions in the same patient group as in the study one is conducting (i.e. FCBT vs FPRT in pediatric OCD). According to the litterature most youths with OCD participating in similar psychotherapeutic RCTs had a CY-BOCS total score around 25 at baseline and after a mean intervention length of 12 weeks, it dropped to around 15 for CBT (approximately a 40% reduction), and to around 20 for relaxation training (approximately a 20% reduction). We expected a symptom improvement in both intervention groups from a CY-BOCS pretreatment score around 25, and we estimated that the smallest post-intervention difference that would probably place one group in the subclinical OCD severity group (CYBOCS 11-15) and the other in the moderate OCD severity group (CY-BOCS 16-24) was a mean difference of 4 points which is also in line with other studies (Cervin M, McGuire JF, D'Souza JM, et al. Efficacy and acceptability of cognitive-behavioral therapy and serotonin reuptake inhibitors for pediatric obsessive-compulsive disorder: a network meta-analysis. *J Child Psychol Psychiatry*. 2024;65(5):594-609. doi:10.1111/jcpp.13934)

Details on participant drop-outs (stopped treatment and further assessments) and treatment non-completion (less than 10/14 sessions in 18 weeks):

FCBT:

1: Treatment non-completion. Stopped treatment after session 9, too demanding. Was assessed at week-16 (possible influence on week-16 assessments of treatment non-completion).

2: Drop-out. Stopped treatments and follow-up assessments after session 4 and assessment week-4, too demanding for patient.

3: Treatment non-completion. Stopped treatment after session 2, too demanding for patient. Start SSRI around week-8. Was assessed at week-16 (possible influence of SSRI on week-16 assessments, but medication status at week-16 unknown).

4: Treatment non-completion. Stopped treatment after session 6, due to other treatment (SSRI). Not assessed at week-8. Was assessed at week-16 (was still in SSRI possible influence of SSRI on week-16 assessments).

5: Stopped treatment after week 6, too demanding for patient. Was assessed at week-8. Drop-out after week-8.

6: Treatment non-completion. Missed different session and had only 9. Participated in all assessments (possible influence on week-16 assessments of treatment non-completion).

7: Stopped treatment after session 9 due to aggravation of OCD symptoms. Drop-out at week-10.

8: Treatment non-completion. Stopped treatment after session 6 due to start of other treatment – SSRI. Was not assessed at week-8. Was assessed at week-16 (was still in SSRI possible influence of SSRI on week-16 assessments).

9: Drop-out before session 1 due to start on medication (SSRI).

10: Treatment non-completion. Missed different session and had only 9 sessions. Stopped after session 11 due to a marked reduction in OCD symptoms, felt no need for more sessions. Was assessed at week-16.

11: Treatment non-completion. Stopped treatment after session 2, started other treatment, not SSRI. Was not assessed at week-4, week-8 or week-16.

12: Treatment non-completion. Missed different session and had only 9 sessions. Participated in all assessments (possible influence on week-16 assessments of treatment non-completion).

Special case 13: Treatment completion but stopped after 10 sessions. One week before week-16, started SSRI. Was assessed at week-16 (was still in SSRI possible influence of SSRI on assessments).

Conclusion FCBT (12 cases):

possible influence on week-16 assessments due to SSRI: n= 4

possible influence on week-16 assessments due to non-completion: n=3

possible influence on week-8 assessments due to non-completion: n=2

no influence on assessments: n=3 (drop out – not assessed), n=1 (non-completion due to recovery)

FPRT:

13: drop-out after session 4 due to aggravation of OCD

14: drop-out after session 3 due to aggravation of OCD, change to other treatment including SSRI.

15: Treatment non-completion. Stopped treatment after session 5, aggravation of OCD. Assessed at week-8 and week-16 (possible influence on week-8 and week-16 assessments of treatment non-completion)

16: Treatment non-completion. Stopped treatment after session 6 due to lack of effect. Not assessed at week-8. Assessed at week-16 (possible influence on week-16 assessments of treatment non-completion)

17: drop-out after session 8 and assessment week-8 due to lack of effect.

18: Treatment non-completion. Stopped treatment after session 6. Assessed at week-8 (possible influence). Drop-out after week-8

19: Treatment non-completion. Stopped treatment after session 7, due to aggravation of OCD. Assessed at week-8 and week-16 (possible influence on week-8 and week-16 assessments of treatment non-completion)

20: Treatment non-completion. Stopped treatment after session 9 due to OCD aggravation, assessed at week-16 (possible influence on week-16 assessments of treatment non-completion)

21: dropout after session 10 (i.e., is treatment completer) due to OCD aggravation.

22: Treatment non-completion. Stopped treatment after session 4 and week-4 assessment due to aggravation of OCD. Assessed at week-8 and week 16 (possible influence on week-8 and week-16 assessments of treatment non-completion)

23: drop-out after session 8 and week-8 due to aggravation of OCD.

24: Treatment non-completion. Stopped treatment after session 7 due to lack of effect. Assessed at week-8 and week-16 (possible influence on week-8 and week-16 assessments of treatment non-completion)

25: Treatment non-completion. Stopped treatment after session 7 due to lack of effect. Assessed at week-8. Drop-out after week-8 assessment. SSRI start after week-8 assessment.

26: Treatment non-completion. Stopped treatment after session 7 due to lack of effect. Assessed at week-8. Start of other treatment, SSRI before week-16. Assessed at week-16 (was still in SSRI possible influence of SSRI on week-16 assessments).

27: drop-out after session 1 due to not compliant with therapy.

28: Treatment non-completion. Stopped treatment after session 7 due to lack of effect. Not assessed at week-16.

29: drop-out after session 12 (i.e., is a treatment completer), due to family decision. Not assessed at week-16.

30: drop-out after session 10 (i.e., is a treatment completer) due to lack of effect.

31: Treatment non-completion. Stopped treatment after session 7 due to change to other treatment (n.a. probably other psychotherapy, not SSRI). Assessed at week-8 and week-16 (possible influence of other treatment on week-8 and week-16 assessments).

32: Treatment non-completion. Stopped treatment after session 6 for practical reasons. Assessed at week-8 (possible influence on week-8 assessments of treatment non-completion). Drop-out after week-8. Not assessed at week-16.

33: Treatment non-completion. Stopped treatment after session 8 and week-8 assessment due to shift to other treatment, not SSRI. Assessed at week-16 (possible influence of other treatment on week-16 assessments).

34: Treatment non-completion. Stopped treatment after session 9. Assessed at week-16 (possible influence on week-16 assessments of treatment non-completion).

35: Treatment non-completion. Stopped treatment after session 6. Assessed at week-8 (possible influence on week-8 assessments of treatment non-completion). Drop-out after week-8 due to too demanding.

36: Treatment non-completion. Stopped treatment after session 6. Assessed at week-8 and week-16 (possible influence on week-8 and week- 16 assessments of treatment non-completion).

Conclusion FPRT:

possible influence on week-16 assessments due to SSRI and/or other psychotherapy: n=4 (n=2 SSRI + n=2 other psychotherapy)

possible influence on week-16 assessments due to non-completion: n=8

possible influence on week-8 assessments due to non-completion: n=10

no influence on assessments: n=6

no influence (were dropouts after treatment completion): n=2

Table-S1

A. **Reasons for drop-out/treatment non-completion:**

	**FCBT N=12**	**FPRT N=24**
Therapy too demanding for participant	4	0
Family decision	0	2
Lack of effect	3	11
OCD aggravation	1	8
Not compliant with therapy	0	1
Missed too many session	2	1
Never started treatment	1	0
Practical reasons	0	1
Recovered	1	0

B. **Details on attendance in therapy sessions**

**No of sessions attended**	**FCBT (n=64) N of participants**	**FPRT (n=66) N of participants**
**0**	**1**	**0**
**1**	**0**	**1**
**2**	**2**	**0**
**3**	**0**	**1**
**4**	**1**	**2**
**5**	**1**	**2**
**6**	**2**	**3**
**7**	**1**	**6**
**8**	**0**	**3**
**9**	**4**	**3**
**10**	**2**	**5**
**11**	**7**	**3**
**12**	**10**	**13**
**13**	**13**	**10**
**14**	**20**	**14**

Table-S2 Family Environment Scale (FES), baseline results

The FES is a 90-item inventory that has a 10 subscales measuring. Scale range 0 (low) to 9 (high). The TECTO study used The Real Form (Form R) which measures people's attitude on their family current environment.

1) Interpersonal Relationship dimension, three subscales (10 items each): cohesion, expressiveness, conflict

2) Personal Growth, four subscales (10 items each): independence, achievement orientation, intellectual-cultural orientation, active-recreational orientation, moral-religious orientation

3) The System Maintenance, two subscales (10 items each): organization, control

The responder (here patient or parent) answers each item, with a “true” or a “untrue”.

	**FCBT (n=64)**	**FPRT (n=66)**	**Total (n=130)**
**FES relationship dimension** patient and parent rated mean standard scores (SD)<br>Scale range, items: 0 -9			
Cohesion patient rated	6.73 (1.73)	6.70 (1.69)	6.71 (1.70)
Missing – n (%)	20 (31.3)	16 (24.2)	36 (27.7)
Cohesion parent rated	7.04 (1.55)	6.91 (1.51)	6.97 (1.53)
Missing – n (%)	10 (15.6)	8 (12.1)	18 (13.8)
Expressiveness patient rated	5.44 (1.53)	5.63 (1.66)	5.54 (1.60)
Missing – n (%)	19 (29.7)	15 (22.7)	34 (26.2)
Expressiveness parent rated	6.54 (1.41)	6.28 (1.53)	6.41 (1.47)
Missing – n (%)	10 (15.6)	9 (13.6)	19 (14.6)
Conflict patient rated	2.48 (2.18)	2.59 (2.29)	2.54 (2.23)
Missing – n (%)	20 (31.3)	15 (22.7)	35 (26.9)
Conflict parent rated	2.81 (1.66)	2.63 (1.98)	2.72 (1.83)
Missing – n (%)	10 (15.6)	8 (12.1)	18 (13.8)
**FES personal growth dimensions** patient and parent rated mean standard scores (SD)<br>Scale range, items: 0-9			
Independence patient rated	5.56 (1.57)	5.55 (1.49)	5.55 (1.52)
Missing– n (%)	19.0 (29.7))	15 (22.7)	34 (26.2)
Independence parent rated	5.99 (1.44)	6.11 (1.56)	6.05 (1.49)
Missing– n (%)	10 (15.6)	9 (13.6)	19 (14.6)
Achievement orientation patient rated	3.55 (2.04)	3.20 (1.89)	3.36 (1.96)
Missing– n (%)	20 (31.3)	15 (22.7)	35 (26.9)
Achievement orientation parent rated	3.06 (1.75)	3.24 (1.58)	3.16 (1.66)
Missing (%)– n (%)	10 (15.6)	8 (12.1)	18 (13.8)
Intellectual-cultural orientation patient rated	3.49 (1.89)	4.53 (2.18)	4.04 (2.10)

	FCBT (n=64)	FPRT (n=66)	Total (n=130)
Missing (%)– n (%)	19 (29.7)	15 (22.7)	34 (26.2)
Intellectual-cultural orientation parent rated	4.41 (1.91)	4.85 (2.04)	4.64 (1.98)
Missing (%)– n (%)	10 (15.6)	8 (12.1)	18 (13.8)
Active-recreational orientation patient rated	4.53 (2.03)	4.53 (2.18)	4.04 (2.10)
Missing (%)– n (%)	19 (29.7)	15 (22.7)	34 (26.2)
Active-recreational orientation parent rated	4.44 (1.72)	4.32 (1.95)	4.38 (1.83)
Missing (%)– n (%)	10 (15.6)	9 (13.6)	19 (14.6)
Moral-religious orientation patient rated	1.98 (1.51)	1.88 (1.36)	1.93 (1.43)
Missing (%)– n (%)	19 (29.7)	17 (25.8)	36 (27.7)
Moral-religious orientation parent rated	1.80 (1.27)	1.72 (1.50)	1.76 (1.38)
Missing (%)– n (%)	10 (15.6)	9 (13.6)	19 (14.6)
**FES system maintenance dimensions** patient and parent rated mean standard scores (SD) Scale range, items: 0-9			
Organization patient rated	5.96 (1.61)	5.73 (1.71)	5.84 (1.65)
Missing (%)– n (%)	19 (29.7)	17 (25.8)	37 (27.7)
Organization parent rated	6.52 (1.27)	6.10 (1.49)	6.30 (1.40)
Missing (%)– n (%)	10 (15.6)	8 (12.1)	18 (13.8)
Control patient rated	3.44 (1.82)	3.06 (1.99)	3.24 (1.91)
Missing (%)– n (%)	19 (29.7)	17 (25.8)	36 (27.7)
Control parent rated	3.48 (1.50)	3.48 (1.72)	3.48 (1.61)
Missing (%)– n (%)	10 (15.6)	8 (12.1)	18 (13.8)

Table-S3 Negative treatment effects measured by NEQ-20 covering week 1-4, week 5-8 and week 9-16

	**Week 1-4**			**Week 5-8**			**Week 9-16**		
	**FCBT**	**FPRT**	**Total**	**FCBT**	**FPRT**	**Total**	**FCBT**	**FPRT**	**Total**
**Median number of participant-rated NEQ-20 score (IQR)**	3.50 [0, 12.0]	4.00 [0, 14.0]	4.00 [0, 14.0]	3.00 [0, 16.0]	3.00 [0, 15.0]	3.00 [0, 16.0]	3.00 [0, 13.0]	3.00 [0, 14.0]	3.00 [0, 14.0]
Missing	20 (31.3)	15 (22.7)	35 (26.9)	17 (26.6)	18 (27.3)	35 (26.9)	17 (26.6)	28 (42.2)	45 (34.6)
**Median number of parent-rated NEQ-20 score (IQR)**	3.00 [1.00, 6.00]	3.00 [1.00, 7.63]	3.00 [1.00, 7.00]	2.00 [0.00, 5.00]	4.00 [2.00, 7.75]	3.00 [1.00, 6.00]	1.00 [1.00, 5.00]	3.50 [0.875, 6.00]	2.00 [1.00, 5.00]
Missing	17 (26.6)	14 (21.2)	31 (23.8)	15 (23.4)	12 (18.2)	27 (20.8)	13 (20.3)	22 (33.3)	35 (26.9)

NEQ = Negative Effects Questionnaire.

Details on change in exploratory outcomes from baseline to end-of treatment (week-16):

Figure-S1 below shows the course of all measures of psychopathology, functioning, and family burden during the trial. The **Table-S4** below furthermore gives details of results.

For exploratory outcomes, parent-reported mean **KIDSCREEN-10** T-score showed a parallel decrease over time as the participant-rated scores, for FCBT from 39.2 (4.88) to 36.0 (3.44) and for FPRT from 39.1 (5.02) to 35.7 (3.76), with no significant group differences at week-16 (estimate 0.45, 95% CI -0.82 to -1.73, p=0.483).

Mean **COIS-R** and median and mean **TOCS scores** decreased with no significant group differences. Participant-rated mean COIS-R decreased for the whole sample from 28.0 (18.0) to 17.5 (15.9) with no significant group differences (estimate = -0.74, 95% CI -7.42 to -5.93, p=0.823), parent-rated mean COIS decreased for the whole sample from 30.9 (19.2) to 18.6 (15.5), with no significant group differences (estimate = -2.64, 95% CI -9.68 to -4.40, p=0.455). Participant-rated median TOCS decreased from 14.0 [2.00 to 25.3] to 1.00 [-31.0 to 17.0] for the whole sample with no significant group differences (w=713.5, p=0.489) and parent-rated mean TOCS decreased from 13.0 to -5.02 with no significant group differences (estimate = -4.94, 95% CI -15.07 to -5.20, p=0.335).

Mean **CGI-S** improved by showing a decrease for the whole sample from 4.29 (0.858) to 3.60 (1.34) and significantly more so for FCBT than FPRT, estimate = - 0.54, 95% CI -1.02 to -0.06, p=0.026. The median CGI-I score at week-16 was significantly more favourable 2.0 [2.0, 2.0] (much improved) for FCBT than for FPRT 3.0 [2.0, 3 .0] (minimally improved), w=1711, p=0.045.

Function improved for the whole sample from median **CGAS** score 55.0 [49.0, 63.0] to 63.0 [54.0, 71.0], showing no significant group differences (w=806, p= 0.152).

Mean parental stress (**PSS**) scores decreased for the whole sample from 35.2 (7.74) at baseline to 33.2 (7.42) with no significant group differences estimate = 0.70, 95% CI -1.56 to 2.97, p=0.539.

Median family accommodation (**FAS**) scores decreased for the whole sample from 15.0 [8.25, 26.5] at baseline to 6.0 [0.50, 13.5] and significantly more so with FCBT than with FPRT, w=1126, p=0.015. At week-16, five participants (7.8%) in the FCBT group reported suicidal thoughts, which did not significantly differ from the number affected in the FPRT group, 2 (3.0%), RR=1.9758, 95% CI 0.4846 to 32841110.8752, p=0.3807.

The number of **SAEs** during the 16 weeks of intervention was n=2 (3.1%) in FCBT (hospital admissions, one with an arm fracture and one with aggravation of OCD) and n=3 (4.5%) in FPRT (hospital admissions, one with somatic complications related to OCD, one with aggravation of OCD and one with arm fracture) (relative risk (RR)=0.7005, 95% CI = 0 to 189074400.0839, p=0.6832).

Response rate (at least 30% reduction on CY-BOCS total score from baseline to week-16) was significantly higher in FCBT, n=30/64 (46.9%) versus in FPRT n=17/66 (25.8%) RR=1.5836, 95% CI 1.0265 to 2.7126, p=0.042. **Remission rate** (participant no longer fulfilling diagnostic criteria for OCD as assessed by K-SADS) was higher in FCBT n=14/64 (21.9%) versus FPRT n=8/66 (12.1%), although not significantly, RR=1.81, 95% CI 0.83 to 4.91, p=0.1385, Figure 4 in manuscript.

Table-S4 Change in psychopathological, functional, and family outcomes during trial.

	Baseline			Week 4			Week 8			Week 16			Total sample change baseline to week 16	p	FCBT vs FPRT week 16 Estimate (95%CI)	p
	FCBT N=64	FPRT N=66	Total N=130	FCBT N=64	FPRT N=66	Total N=130	FCBT N=64	FPRT N=66	Total N=130	FCBT N=64	FPRT N=66	Total N=130				
**Mean CY-BOCS score (SD)**	25.8 (4.90)	25.8 (4.96)	25.8 (4.92)	23.0 (6.18)	24.3 (5.23)	23.7 (5.72)	20.1 (5.92)	22.9 (5.34)	21.6 (5.79)	15.9 (8.8)	19.9 (8.09)	17.8 (8.09)	Estimate= -0.45	P<0·001	-3.92 (CI -6.85 to -0.99)	**0.009**
Analysed, n /missing, n (%)	64 /0 (0)	66 /0 (0)	130 /0 (0)	57 /7 (10.9)	62 /4 (6.1)	119 /11 (88.5)	57/ 7 (10.9)	59 /7 (10.6)	116 /14 (10.8)	59 /5 (7.8)	52 /14 (21.2)	111 /19 (14.6)				
**Mean KIDSCREEN-10 patient T-score SD)**	41.0 (6.46)	43.0 (6.13)	42.0 (6.34)	43.0 (7.29)	41.6 (6.81)	42.3 (7.07)	41.3 (5.19)	39.1 (5.16)	40.3 (5.27)	37.3 (5.04)	36.4 (4.53)	36.9 (4.81)	Estimate= -0.45	P<0·001	0.96 (CI -0.97 to 2.88)	0·323
Analysed, n /missing, n (%)	52 /12 (18.8)	53 /13 (19.7)	105 /25 (19.2)	47 /17 (26.6)	41 /25 (37.9)	88 /42 (32.3)	43 /21 (32.8)	36 /30 (45.5)	79 /51 (39.2)	41 /23 (35.9)	30 /36 (54.5)	71 /59 (45.4)				
**Mean KIDSCREEN-10 parent T-score (SD)**	39·2 (4·88)	39·1 (5·02)	39·1 (4·93)	39.5 (4.89)	39.5 (4.48)	39.5 (4.67)	38.9 (4.31)	37.2 (4.45)	38.0 (4.44)	36.0 (3.44)	35.7 (3.76)	35.8 (3.58)			0.45 (CI -0.82 to -1.73)	0·483
Analysed, n /missing, n (%)	56 /8 (12·5)	60 /6 (9·1)	116 /14 (10·8)	51 /13 (20.3)	51 /15 (22.7)	102 /28 (21.5)	52 /12 (18.8)	49 /17 (25.8)	101 /29 (22.3)	44 /20 (31.3)	42 /24 (36.4)	86 /44 (33.8)				
**Mean COIS-R participant-score (SD)**	26.5 (16.4)	29.3 (19.3)	28.0 (18·0)	23.0 (14.0)	31.7 (20.6)	27.1 (17·8)	20.6 (14.6)	23.1 (17.6)	21.8 (16.0)	16.9 (15.1)	18.2 (17.1)	17.5 (15.9)			-0.74 (CI -7.42 to -5.93)	0.823
Analysed, n /missing, n (%)	50 /14 (21.9)	56 /10 (15·2)	106 /24 (18·5)	53 /11 (17·2)	47 /19 (29·8)	100 /30 (23·1)	49 /15 (23·4)	44 /22 (33·3)	93 /37 (28·5)	46 /18 (28·1)	40 /26 (39.40)	86 /44 (33·8)				
**Mean COIS-R parent-score (SD)**	29.4 (17.3)	32.2 (20.9)	30.9 (19·2)	26.6 (14.3)	27.9 (18.7)	27.3 (16.6)	23.3 (15.2)	25.4 (18.3)	24.3 (16.7)	18.0 (15.1)	19.3 (16.2)	18.6 (15.5)			-2.64 (CI -9.68 to -4.40)	0.455
Analysed, n /missing, n (%)	53 /11 (17·2)	57 /9 (13·6)	110 /20 (15·4)	52 /12 (18·8)	53 /13 (19·7)	105 /25 (19·2)	55 /9 (14·1)	55 /11 (16·7)	110 /20 (15·4)	51 /13 (20·3)	48 /18 (27·3)	99 /31 (23·8)			Observati ons 57	

	Baseline			Week 4			Week 8			Week 16			Total sample change baseline to week 16	p	FCBT vs FPRT week 16 Estimate (95%CI)	p
	FCBT N=64	FPRT N=66	Total N=130	FCBT N=64	FPRT N=66	Total N=130	FCBT N=64	FPRT N=66	Total N=130	FCBT N=64	FPRT N=66	Total N=130				
**Median TOCS participant-score (IQR)**	17.0 [3.00,28·0]	11.0 [5.00,23·5]	14.0 [2.00,25·3]							-3.00 [-32.5, 12.5]	7.00 [-27.5, 19.0]	1.00[-31.0, 17.0]			W=713.5	0.489
Analysed, n /missing, n (%)	49 /15 (23.4)	51 /15 (22.7)	114 /30 (23·1)							42 /22 (34.4)	31 /35 (53.0)	73 /57 (43.8)				
**Mean TOCS parent-score (SD)**	12.7 (18·2)	13.4 (17·2)	13.0 (18·7)							-7.99 (23.4)	-1.99 (26.9)	-5.02 (27.2)			-4.94 (CI -15.07 to -5.20)	0.335
Analysed, n /missing, n (%)	57 /7 (10.9)	57 /9 (13.6)	114 /16 (12·3)							43 /21 (32.8)	42 /24 (36.4)	85 /45 (34.6)				
**Mean CGI-S score (SD)**	4.27 (0.86)	4.32 (0.86)	4.29 (0·86)	4.16 (0.96)	4.47 (0.90)	4.32 (0·94)	3.88 (0.965)	4.25 (0.856)	4.07 (0.926)	3.33 (1.31)	3.90 (1.33)	3.60 (1.34)			-0.54 (CI -1.02 to -0.06	**0.026**
Analysed, n /missing, n (%)	64 /0 (0)	66 /0 (0)	130 /0 (0)	57 /7 (10.9)	62 /4 (6.1)	119 /11 (8·5)	57 /7 (10.9)	60 /6 (9.1)	117 /13 (10.0)	57 /7 (10.9)	50 /16 (24.2)	107 /23 (17.7)				
**Median CGI-I score (IQR)****				4.0 [3.0, 4.0]	4.0 [3.0, 4.0]	4.00 [3·00,4·00]	3.0 (2.0, 3.0)	3.0 (3.0, 3.0)	3.0 [2.00, 3.00]	2.0 (2.0, 2.0)	3.0 (2.0, 3.0)	2.0 [2.00, 2.00]			W=1711	**0.045**
Analysed, n /missing, n (%)				57 /7 (10.9)	62 /4 (6.1)	119 /11 (8·5)	57 /7 (10.9)	60 /6 (9.1)	117 /13 (10.0)	57 /7 (10.9)	51 /15 (22.7)	108 /22 (16·9)				

	Baseline			Week 4			Week 8			Week 16			Total sample change baseline to week 16	p	FCBT vs FPRT week 16 Estimate (95%CI)	p
	FCBT N=64	FPRT N=66	Total N=130	FCBT N=64	FPRT N=66	Total N=130	FCBT N=64	FPRT N=66	Total N=130	FCBT N=64	FPRT N=66	Total N=130				
**Median CGAS score (IQR)**	55.0 (49.8,65.0)	55.0 (49.0,62.0)	55.0 (49.0,63.0)							65.0 (57.0,75.0)	61.0 (50.0,68.3)	63.0 (54.0,71.0)			W=806	0.152
Analysed, n /missing, n (%)	64/ 0 (0)	65 /1 (1.5)	129 /1 (0·8)							49 /15 (23.4)	40 /26 (39.4)	89 /41 (31.5)				
**Suicidal thoughts, K-SADS score (2 or 3), n (%)**	6 (9.4)	5 (7.6)	11 (8·5)							5 (7.8)	2 (3.0)	7 (5.58)			RR=1.975 8 CI 0.4846 to 30211093 7.5072	0.381
Analysed, n /missing, n (%)	60 /4 (6.3)	61 /4 (6.1)	122 /8 (6·2)							46 /18 (28.1)	34 /32 (48.5)	80 /50 (38.5)				
**Mean PSS score (SD)**	36.0 (7.94)	34.4 (7·55)	35.2 (7·74)	35.5 (6.26)	34.9 (8.16)	35.2 (7.25)	35.0 (6.67)	34.2 (9.13)	34.7 (7.91)	33.5 (6.47)	32.9 (8.41)	33.2 (7.42)			0.70 (CI - 1.56 to 2.97)	0.539
Analysed, n /missing, n (%)	55 /9 (14.1)	58 /8 (12.1)	113 /17 (13·1)	50 /14 (21.9)	61 /15 (22.7)	101 /29 (22.3)	53 /11 (17.2)	49 /17 (25.8)	102 /28 (21.5)	44 /20 (31.3)	40 /26 (39.4)	84 /46 (35.4)				
**Median FAS score (IQR)**	14.0 (8.0,26.0)	16.0 (9.25,28.4)	15.0 [8.25,26.5]	11.0 [5.63,19.4]	14.0 [6.0,29.0]	13.0 [5.75, 2.8]	8.25 (2.50, 21.0)	10.0 (5.13,19.5)	9.25 [4.00, 20.4]	2.00 (0,10.3)	10.0 (2.38,22.6)	6 [0.500, 13.5]			W=1126	**0.015**
Analysed, n /missing, n (%)	53 /11 (17.2)	58 /8 (12.1)	111 /19 (14·6)	50 /14 (21.9)	49 /17 (25.8)	99 /31 (23·8)	52 /12 (18.8)	46 /20 (30.3)	98 /32 (24·6)	43 /21 (32.8)	40 /26 (39.4)	83 /47 (36.2)				

* Self-reported from age: 11 years. Parent-reported from age: 8–17 years, ** Not at assessed at baseline

CGAS The Children's Global Assessment Scale; *CGI-S The Clinical Global Impression Scale - severity (1=Normal – not at all ill, 2=Borderline mentally ill, 3= Mildly il, 4=Moderately ill, 5=Markedly ill, 6=Severely ill, 7=Among the most extremely ill patients; CGI-I* The Clinical Global Impression Scale – improvement (compared to baseline 1=Very much improved, 2=Much improved, 3=Minimally improved, 4=No change, 5=Minimally worse, 6=Much worse, 7=Very much worse); *COIS-R* Child Obsessive Compulsive Disorder Impact Scale Revised; *CY-BOCS* Children's Yale-Brown Obsessive-Compulsive Scale; *FAS* Family Accommodation Scale; *Screening Instrument for Children and Adolescents KIDSCREEN; K-SADS-PL* Kiddie-Schedule for Affective Disorders and Schizophrenia; *PSS* Parental Stress Scale; *TOCS* Toronto Obsessive-Compulsive Rating Scale.*W* Wilcoxon rank sum

Figure-S1 The course of all symptoms, level of functioning, and family-related outcomes

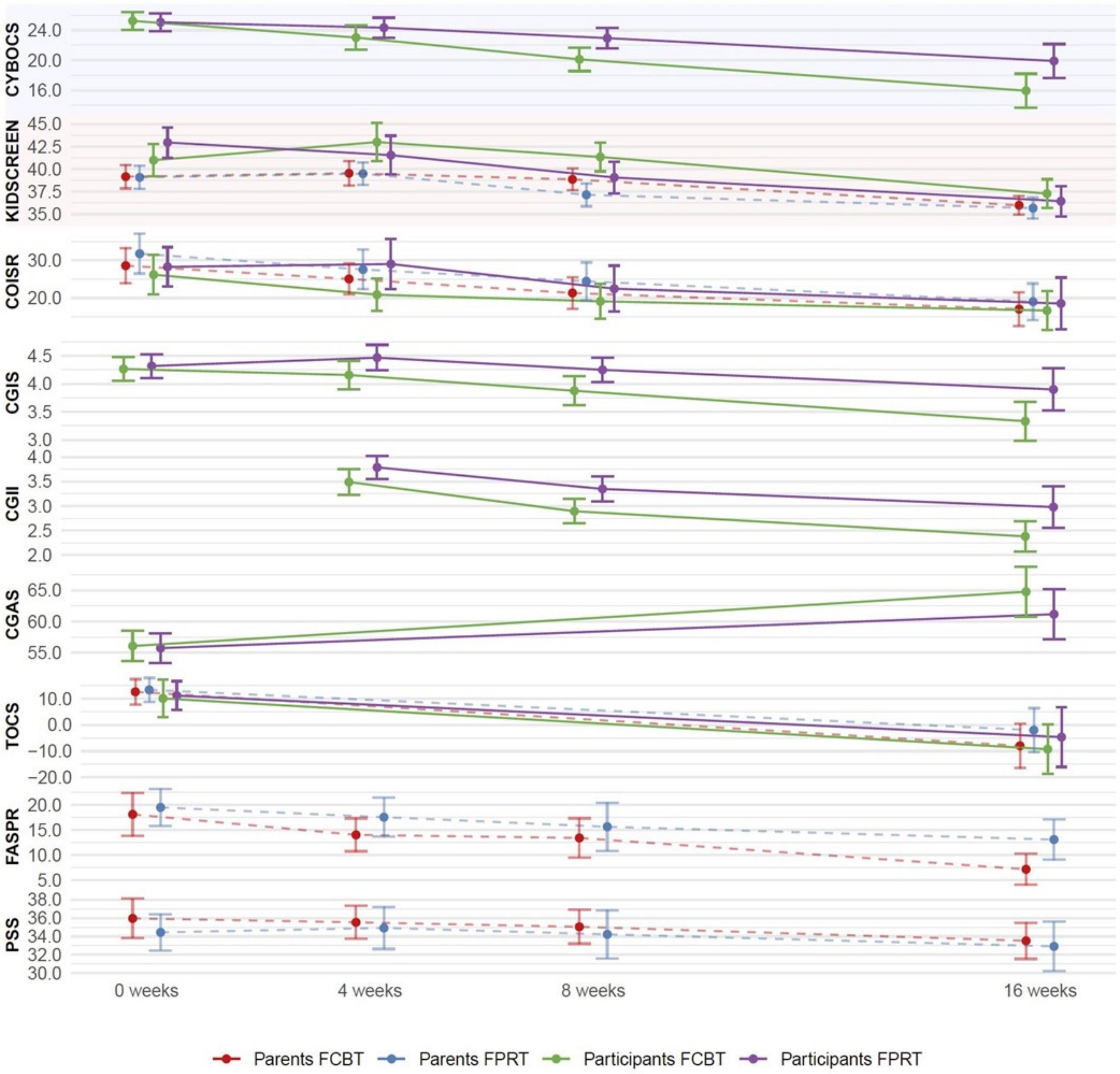

Table-S5 KIDCREEN-52, 10 dimensions, post-hoc analysis

Dimensions	Baseline		Week 4		Week 8		Week 16		FCBT vs FPRT week 16 Estimate (95%CI)	p
	FCBT	FPRT	FCBT	FPRT	FCBT	FPRT	FCBT	FPRT		
**Physical well-Being patient mean score (95% CI)**	49.47 (46.58;52.36)	47.29 (43.77;50.80)	46.54 (42.48;50.60)	45.85 (42.39;39.31)	51.04 (46.72;55.35)	49.21 (45.02;52.40)	46.27 (41.31;51.23)	48.49 (43.99;53.00)	-3.41 (-10.61;3.78)	0.346
Missing, n (%)	12 (18.8)	12 (18.2)	16 (25)	25 (37.9)	20 (31.2)	29 (43.9)	22 (24.4)	34 (51.5)		
**Physical well-Being parent mean score (95% CI)**	44.99 (42.38;47.60)	42.08 (39.60;44.56)	46.48 (44.12;48.85)	44.88 (42.24;47.51)	47.32 (44.60;50.04)	45.16 (42.54;47.79)	48.85 (46.02;51.68)	47.28 (44.40;50.15)	0.48 (-3.38;4.35)	0.804
Missing, n (%)	8 (12.5)	6 (9.1)	13 (20.3)	15 (22.7)	11 (17.2)	16 (24.2)	20 (31.2)	24 (36.4)		
**Psychological well-Being patient mean score (95% CI)**	42.44 (39.70;45.18)	44.25 (41.42;47.08)	46.25 (42.52;47.97)	43.20 (40.22;46.17)	48.39 (45.40;51.38)	44.86 (42.00;47.72)	49.79 (46.16;53.44)	47.20 (43.79;50.61)	3.5 (-1.78;8.09)	0.207
Missing, n (%)	12 (18.8)	13 (19.7)	15 (23.4)	24 (36.4)	20 (31.2)	29 (43.9)	22 (34.4)	34 (51.5)		
**Psychological well-Being parent mean score (95% CI)**	38.47 (36.35;40.60)	38.52 (36.66;40.40)	39.90 (37.96;41.84)	38.42 (36.46;40.38)	42.59 (40.59;44.60)	40.24 (38.15;42.33)	44.67 (42.28;47.06)	42.51 (40.30;44.72)	2.71 (-0.01;5.43)	0.050
Missing, n (%)	8 (12.5)	6 (9.1)	14 (21.9)	15 (22.7)	11 (17.2)	16 (24.2)	20 (31.2)	24 (36.4)		
**Moods and emotions patient mean score (95% CI)**	44.60 (41.92;47.29)	44.67 (42.23;47.10)	46.86 (44.08;49.65)	45.48 (42.12;48.85)	50.04 (47.55;52.52)	45.27 (42.07;48.48)	49.51 (45.81;53.21)	47.12 (43.22;51.01)	0.53 (-3.92;4.98)	0.812
Missing, n (%)	12 (18.8)	12 (18.2)	16 (25.0)	24 (36.4)	20 (31.2)	29 (43.9)	23 (35.9)	34 (51.5)		
**Moods and emotions parent mean score (95% CI)**	41.02 (39.23;42.82)	41.35 (39.62;43.09)	43.35 (41.30;45.39)	42.40 (40.30;44.50)	46.36 (44.21;48.48)	44.46 (42.12;46.80)	47.61 (45.06;50.15)	46.83 (44.34;49.32)	1.23 (-1.79;4.25)	0.421
Missing, n (%)	8 (12.5)	6 (9.1)	13 (20.3)	15 (22.7)	11 (17.2)	16 (24.2)	20 (31.2)	24 (36.4)		
**Self perception patient mean score (95% CI)**	46.52 (43.85;49.20)	48.85 (45.13;50.56)	49.51 (46.56;52.46)	48.81 (44.56;51.06)	50.45 (46.78;54.12)	48.43 (44.82;52.05)	49.43 (45.69;53.16)	48.43 (45.48; 51.37)	-0.47 (-4.06;3.13)	0.795
Missing, n (%)	12 (18.8)	13 (19.7)	16 (25.0)	24 (36.4)	20 (31.2)	29 (43.9)	23 (35.9)	34 (51.5)		
**Self perception parent mean score (95% CI)**	44.41 (42.82;46.00)	44.49 (42.79;46.29)	45.82 (44.12;47.52)	44.98 (43.10;46.85)	47.16 (45.21;49.11)	46.44 (44.35;48.52)	48.48 (46.16;50.82)	48.19 (46.14;50.25)	0.28 (-1.93;2.49)	0.803
Missing, n (%)	8 (12.5)	6 (9.1)	13 (20.3)	15 (22.7)	11 (17.2)	16 (24.2)	20 (31.2)	24 (36.4)		
**Autonomy patient mean score (95% CI)**	46.18 (43.79;48.65)	47.94 (45.49;50.39)	48.58 (45.89;51.27)	47.86 (45.22;50.50)	51.05 (48.03;54.07)	49.64 (46.41;52.87)	52.42 (49.13;55.70)	51.96 (48.13;55.79)	1.53 (-2.81;5.87)	0.483

Dimensions	Baseline		Week 4		Week 8		Week 16		FCBT vs FPRT week 16 Estimate (95%CI)	p
	FCBT	FPRT	FCBT	FPRT	FCBT	FPRT	FCBT	FPRT		
Missing, n (%)	12 (18.8)	12 (18.2)	16 (25.0)	24 (36.4)	20 (31.2)	28 (43.9)	22 (34.4)	35 (53.0)		
**Autonomy parent mean score (95% CI)**	47.32 (45.62;49.02)	47.43 (45.50;49.37)	46.99 (45.12;48.86)	49.34 (47.12;51.55)	49.55 (47.61;51.50)	49.71 (47.40;52.01)	51.65 (49.19;54.10)	51.68 (49.01;54.35)	0.20 (-2.82;3.21)	0.896
Missing, n (%)	8 (12.5)	6 (9.1)	13 (20.3)	15 (22.7)	11 (17.2)	17 (25.8)	20 (31.2)	24 (36.4)		
**Parent relations and home life patient mean score (95% CI)**	47.99 (45.51;50.46)	48.77 (46.40;51.15)	49.56 (47.14;51.98)	46.56 (44.00;49.13)	51.14 (48.37;53.91)	47.12 (44.03;50.22)	51.44 (48.38;54.49)	49.13 (45.83;52.43)	2.42 (-1.23;6.05)	0.191
Missing, n (%)	12 (18.8)	13 (19.7)	16 (25.0)	24 (36.4)	20 (31.2)	29 (43.9)	22 (34.4)	34 (51.5)		
**Parent relations and home life parent mean score (95% CI)**	41.99 (40.31;43.67)	42.19 (40.74;43.64)	42.91 (41.46;44.37)	42.65 (40.61;44.68)	44.75 (43.00;46.51)	43.50 (41.30;45.69)	45.36 (43.44;47.29)	45.12 (42.90;47.34)	1.32 (-0.47;3.12)	0.146
Missing, n (%)	8 (12.5)	7 (10.6)	14 (21.9)	16 (24.2)	11 (17.2)	16 (24.2)	20 (31.2)	24 (36.4)		
**Financial resources patient mean score (95% CI)**	55.49 (53.21;57.77)	56.13 (53.98;58.29)	55.39 (52.90;57.89)	55.57 (52.73;58.41)	55.31 (52.49;58.13)	56.19 (52.97;59.40)	56.55 (53.18;59.92)	56.29 (52.93;59.64)	-1.53 (-5.41;2.34)	0.432
Missing, n (%)	13 (20.3)	13 (19.7)	16 (25.0)	24 (36.4)	20 (31.2)	30 (45.5)	22 (34.4)	34 (51.5)		
**Financial resources parent mean score (95% CI)**	52.15 (49.97;54.34)	52.66 (50.32;55.00)	51.89 (49.59;54.18)	54.01 (51.94;56.09)	55.13 (53.05;57.20)	54.01 (51.75;56.25)	53.43 (50.80;56.06)	55.05 (53.04;57.06)	-1.09 (-3.96;1.78)	0.452
Missing, n (%)	8 (12.5)	7 (10.6)	13 (20.3)	15 (22.7)	11 (17.2)	16 (24.2)	20 (31.2)	24 (36.4)		
**Peers and social support patient mean score (95% CI)**	42.11 (38.79;45.44)	43.88 (41.25;46.59)	43.23 (40.54;45.91)	41.27 (38.07;44.46)	46.17 (42.45;49.90)	43.04 (39.48;46.59)	47.33 (43.40;51.25)	44.87 (41.61;48.14)	2.93 (-1.50;7.36)	0.190
Missing, n (%)	14 (21.9)	12 (18.2)	16 (25.0)	25 (37.9)	21 (32.8)	30 (45.5)	23 (35.9)	34 (51.5)		
**Peers and social support parent mean score (95% CI)**	39.09 (36.86;41.31)	37.81 (35.40;40.23)	39.31 (37.17;41.45)	37.94 (35.21;40.67)	41.56 (39.21;43.92)	39.31 (37.00;41.62)	42.25 (39.34;45.15)	42.13 (39.37;44.88)	-0.55 (-3.60;2.49)	0.717
Missing, n (%)	8 (12.5)	8 (12.1)	13 (20.3)	15 (22.7)	11 (17.2)	16 (24.2)	20 (31.2)	24 (36.4)		
**School environment patient mean score (95% CI)**	47.43 (45.07;49.80)	48.30 (45.53;51.07)	49.13 (46.00;52.25)	45.53 (41.66;49.40)	51.05 (46.95;55.15)	47.43 (43.29;51.47)	53.62 (49.81;57.44)	50.30 (46.18;54.43)	3.08 (-1.57;7.74)	0.190
Missing, n (%)	13 (20.3)	13 (19.7)	16 (25.0)	24 (36.4)	21 (32.8)	30 (45.5)	23 (35.9)	36 (54.5)		
**School environment**	45.45 (43.70;47.19)	46.99 (44.73;49.24)	44.38 (41.86;46.89)	45.15 (42.59;47.70)	47.97 (45.51;50.43)	45.85 (43.14;48.55)	49.49 (47.13;51.86)	48.79 (45.72;51.85)	2.17 (-1.21;5.54)	0.205

Dimensions	Baseline		Week 4		Week 8		Week 16		FCBT vs FPRT week 16 Estimate (95%CI)	p
	FCBT	FPRT	FCBT	FPRT	FCBT	FPRT	FCBT	FPRT		
**parent mean score (95% CI)**										
Missing, n (%)	8 (12.5)	6 (9.1)	13 (20.3)	15 (22.7)	12 (18.8)	17 (25.8)	20 (31.2)	24 (36.4)		
**Bullying patient mean score (95% CI)**	50.93 (48.18;53.68)	50.89 (48.42;53.37)	50.93 (48.09;53.76)	51.46 (48.51;54.42)	53.40 (50.62;56.18)	52.18 (49.41;54.96)	52.87 (49.59;56.14)	52.81 (49.45;56.18)	-0.08 (-4.80;4.64)	0.972
Missing, n (%)	11 (17.2)	12 (18.2)	15 (23.4)	24 (36.4)	20 (31.2)	29 (43.9)	22 (34.4)	34 (51.5)		
**Bullying parent mean score (95% CI)**	46.78 (44.20;49.35)	48.26 (45.73;50.79)	48.37 (45.90;50.84)	50.39 (47.78;53.01)	52.29 (49.97;54.62)	50.76 (48.18;53.34)	52.78 (50.53;55.03)	50.86 (48.29;53.44)	1.53 (-1.46;4.52)	0.311
Missing, n (%)	8 (12.5)	7 (10.6)	13 (20.3)	15 (22.7)	11 (17.2)	17 (25.8)	20 (31.2)	24 (36.4)		

Dimensions: Physical Well-Being (5 items); Psychological Well-Being (6 items); Moods and Emotions (7 items); Self Perception (5 items); Autonomy (5 items); Parent Relations and Home Life (6 items); Financial Resources (3 items); Peers and Social Support (6 items); School Environment (6 items); Bullying (3 items)

As part of our statistical analysis plan, we reported health-related quality-of-life (HRQoL) with the Screening Instrument for Children and Adolescents (KIDSCREEN-10 Index) based on assessment with KIDSCREEN-52. However, after our analyses were completed, a new study examining the criterion-related construct validity and psychometric properties of the Danish language version of the KIDSCREEN-10 using Rasch models was published (please see manuscript) concluding that the child/adolescent self-report questionnaire cannot be recommended for use in population-level or small sample studies. We therefore decided to perform a post-hoc analysis using the data from the full KIDSCREEN-52 version on which our KIDSCREEN-10 index was based.

The mean participant-rated KIDSCREEN-10 T-score, decreased significantly for the whole sample from 42.0 (SD=6.34) to 36.9 (SD=4.81) (estimate -0.45, p<0.001), in the FCBT group from 41.0 (SD=6.46) to 37.3 (SD=5.04) and in the FPRT group from 43.0 (SD=6.13) to 36.4 (SD=4.53) with no significant difference between intervention groups at week-16 (estimate 0.96, 95% CI -0.97 to – 2.88, p=0.323). The post-hoc analysis of KIDSCREEN-52 showed slightly increasing T-scores on all 10 dimensions with no significant differential group effects at week-16. The parent-reported KIDSCREEN-10 T-score showed at parallel decrease over time as the participant-rated scores with no significant group differences at week-16. In contrast, parent reported KIDSCREEN-52 scores increased on all 10 dimensions (except for physical well-being in the FCBT group) with no significant differential group effects at week-16 (please see table above and figure below).

Figure-S2 KIDSCREEN-52, 10 dimensions

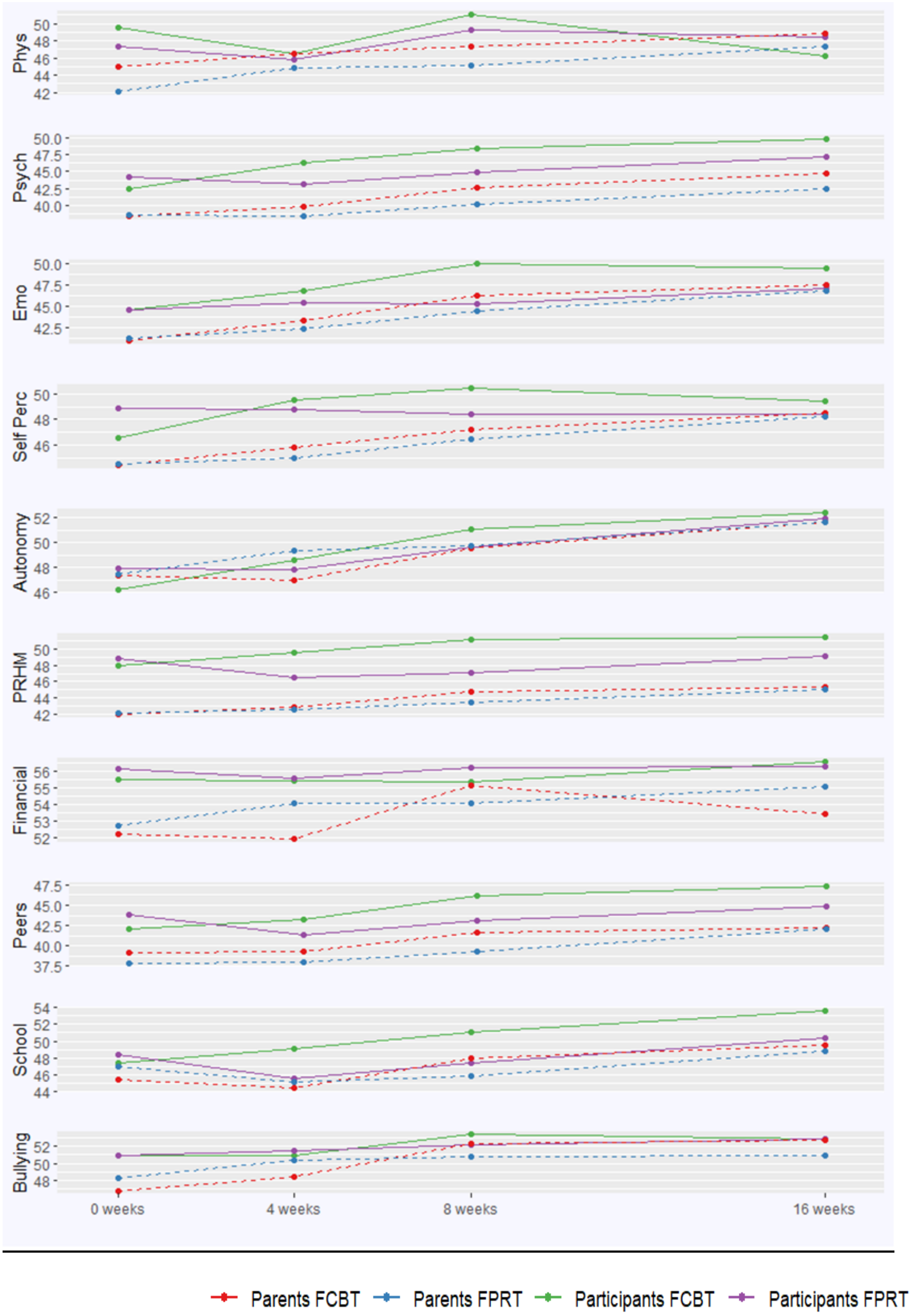

Phys: Physical well-being; Psych: Psychological well-being; Emo: Moods and Emotions; Self Perc: Self Perception; Autonomy: Autonomy; PRHM: Parent Relations and Home Life; Financial: Financial Resources; Peers: Peers and Social Support; School: School Environment; Bullying: Bullying.

Moderating effect of therapy factors on treatment outcomes: Neither therapist confidence in treatment (p=0.3869), nor participant motivation for treatment (average of assessments from week 0, 1, 4, 8, and 14 on 7-point Likert scale; estimate -5.57, 95%CI -11.44 to -0.31, p=0.063), nor therapeutic alliance (average of TASC-R assessments from week 1, 4, 8, and 16; estimate = -0.38, 95%CI -0.40 to -0.64, p=0.461) or compliance (estimate = -0.40, 95%CI -3.58 to 2.78, p=0.801) significantly moderated treatment outcome.

Table-6 Therapy factors – confidence, motivation, alliance, and compliance

1. Confidence in treatment

Therapist rated confidence in treatment assessed after the first session on a 7-point Likert scale (1=no confidence at all 7=high confidence)

	FCBT (N=64)	FPRT (N=66)	Overall (N=130)
Mean (SD)	5.50 (1.15)	4.53 (0.903)	4.97 (1.13)
Median [Min, Max]	5.50 [3.00, 7.00]	5.00 [3.00, 6.00]	5.00 [3.00, 7.00]
Missing	16 (25.0%)	8 (12.1%)	24 (18.5%)

2. Motivation for treatment

Patient rated motivation for treatment at week 0, 1, 4, 8, 14; 7-point Likert scale (1=not at all motivated; 7=highly motivated)

Session number	FCBT (N=64)	FPRT (N=66)	Total (N=130)
**Baseline**			
Mean (SD)	5.16 (1.59)	5.62 (1.69)	5.40 (1.65)
Median [Min, Max]	5.00 [1.00, 7.00]	6.00 [1.00, 7.00]	6.00 [1.00, 7.00]
Missing	15 (23.4%)	14 (21.2%)	29 (22.3%)
**Session 1**			
Mean (SD)	5.45 (1.76)	5.62 (1.28)	5.54 (1.52)
Median [Min, Max]	6.00 [1.00, 7.00]	6.00 [2.00, 7.00]	6.00 [1.00, 7.00]
Missing	20 (31.3%)	19 (28.8%)	39 (30.0%)
**Session 4**			
Mean (SD)	5.53 (1.62)	5.05 (1.67)	5.30 (1.65)
Median [Min, Max]	6.00 [1.00, 7.00]	5.00 [2.00, 7.00]	6.00 [1.00, 7.00]

Session number	FCBT (N=64)	FPRT (N=66)	Total (N=130)
Missing	19 (29.7%)	24 (36.4%)	43 (33.1%)
**Session 8**			
Mean (SD)	5.65 (1.48)	5.00 (1.52)	5.36 (1.52)
Median [Min, Max]	6.00 [1.00, 7.00]	5.00 [2.00, 7.00]	6.00 [1.00, 7.00]
Missing	21 (32.8%)	32 (48.5%)	53 (40.8%)
**Session 14**			
Mean (SD)	5.74 (1.44)	5.45 (1.23)	5.60 (1.34)
Median [Min, Max]	6.00 [1.00, 7.00]	6.00 [3.00, 7.00]	6.00 [1.00, 7.00]
Missing	30 (46.9%)	35 (53.0%)	65 (50.0%)

3. Therapeutic alliance

Patient rated at week 1, 4, 8, and 16 on Therapeutic Alliance Scale for Children–revised (TASC-R) , a 12-item, 4-point scale (1 (not at all) to 4 (very much)), range 12-48

Session number	FCBT (N=64)	FPRT (N=66)	Total (N=130)
**Session 1**			
Mean (SD)	30.6 (2.50)	29.5 (2.89)	30.0 (2.75)
Median [Min, Max]	31.0 [25.0, 35.0]	30.0 [23.0, 35.0]	30.0 [23.0, 35.0]
Missing	31 (48.4%)	27 (40.9%)	58 (44.6%)
**Session 4**			
Mean (SD)	30.8 (4.16)	29.7 (3.26)	30.3 (3.77)
Median [Min, Max]	31.0 [20.0, 42.0]	29.0 [22.0, 39.0]	30.0 [20.0, 42.0]
Missing	20 (31.3%)	24 (36.4%)	44 (33.8%)
**Session 8**			
Mean (SD)	30.7 (3.57)	29.8 (2.75)	30.3 (3.26)
Median [Min, Max]	31.0 [22.0, 37.0]	30.5 [25.0, 36.0]	31.0 [22.0, 37.0]
Missing	21 (32.8%)	34 (51.5%)	55 (42.3%)
**Session 16**			
Mean (SD)	31.2 (4.32)	30.4 (3.33)	30.8 (3.90)
Median [Min, Max]	32.0 [22.0, 41.0]	31.0 [20.0, 37.0]	31.0 [20.0, 41.0]
Missing	26 (40.6%)	35 (53.0%)	61 46.9%)

4. Therapy compliance

Therapist rated patient compliance with treatment at session # 2 to # 14 (session # 1 excluded because prior homework has not yet been given). 7-point Likert scale (1=no compliance at all; 7=very high compliance)

	FCBT (N=64)	FPRT (N=66)	Total (N=130)
**Session 2**<br>Mean (SD)	5.30 (1.16)	5.15 (1.44)	5.22 (1.31)
Median [Min, Max]	6.00 [2.00, 7.00]	6.00 [1.00, 7.00]	6.00 [1.00, 7.00]
Missing	7 (10.9%)	6 (9.1%)	13 (10.0%)
**Session 3**<br>Mean (SD)	5.05 (1.61)	5.16 (1.42)	5.11 (1.51)
Median [Min, Max]	6.00 [1.00, 7.00]	6.00 [1.00, 7.00]	6.00 [1.00, 7.00]
Missing	6 (9.4%)	5 (7.6%)	11 (8.5%)
**Session 4**<br>Mean (SD)	5.16 (1.71)	5.13 (1.63)	5.15 (1.66)
Median [Min, Max]	6.00 [1.00, 7.00]	6.00 [1.00, 7.00]	6.00 [1.00, 7.00]
Missing	9 (14.1%)	4 (6.1%)	13 (10.0%)
**Session 5**<br>Mean (SD)	5.47 (1.36)	5.28 (1.36)	5.37 (1.36)
Median [Min, Max]	6.00 [1.00, 7.00]	6.00 [1.00, 7.00]	6.00 [1.00, 7.00]
Missing	13 (20.3%)	8 (12.1%)	21 (16.2%)
**Session 6**<br>Mean (SD)	4.90 (1.64)	4.91 (1.57)	4.91 (1.60)
Median [Min, Max]	5.00 [1.00, 7.00]	6.00 [1.00, 7.00]	5.00 [1.00, 7.00]
Missing	12 (18.8%)	9 (13.6%)	21 (16.2%)
**Session 7**<br>Mean (SD)	5.47 (1.52)	5.21 (1.30)	5.33 (1.40)
Median [Min, Max]	6.00 [1.00, 7.00]	6.00 [1.00, 7.00]	6.00 [1.00, 7.00]
Missing	17 (26.6%)	10 (15.2%)	27 (20.8%)
**Session 8**<br>Mean (SD)	5.35 (1.31)	5.14 (1.24)	5.26 (1.28)
Median [Min, Max]	6.00 [2.00, 7.00]	6.00 [2.00, 7.00]	6.00 [2.00, 7.00]

	FCBT (N=64)	FPRT (N=66)	Total (N=130)
Missing	13 (20.3%)	24 (36.4%)	37 (28.5%)
**Session 9** Mean (SD)	5.47 (1.73)	5.16 (1.44)	5.33 (1.61)
Median [Min, Max]	6.00 [1.00, 7.00]	6.00 [1.00, 7.00]	6.00 [1.00, 7.00]
Missing	17 (26.6%)	28 (42.4%)	45 (34.6%)
**Session 10** Mean (SD)	5.15 (1.27)	4.95 (1.41)	5.05 (1.34)
Median [Min, Max]	5.00 [2.00, 7.00]	5.00 [1.00, 7.00]	5.00 [1.00, 7.00]
Missing	25 (39.1%)	29 (43.9%)	54 (41.5%)
**Session 11** Mean (SD)	4.88 (1.59)	5.35 (1.06)	5.10 (1.38)
Median [Min, Max]	5.00 [2.00, 7.00]	6.00 [3.00, 7.00]	5.50 [2.00, 7.00]
Missing	21 (32.8%)	29 (43.9%)	50 (38.5%)
**Session 12** Mean (SD)	5.17 (1.61)	4.93 (1.70)	5.07 (1.64)
Median [Min, Max]	6.00 [1.00, 7.00]	5.50 [1.00, 7.00]	6.00 [1.00, 7.00]
Missing	22 (34.4%)	36 (54.5%)	58 (44.6%)
**Session 13** Mean (SD)	5.42 (1.54)	5.23 (1.19)	5.35 (1.41)
Median [Min, Max]	6.00 [1.00, 7.00]	5.00 [3.00, 7.00]	6.00 [1.00, 7.00]
Missing	26 (40.6%)	44 (66.7%)	70 (53.8%)
**Session 14** Mean (SD)	5.10 (1.48)	5.47 (1.25)	5.26 (1.39)
Median [Min, Max]	5.00 [1.00, 7.00]	6.00 [2.00, 7.00]	6.00 [1.00, 7.00]
Missing	25 (39.1%)	36 (54.5%)	61 (46.9%)

Results of manual fidelity ratings:

We evaluated treatment fidelity and adherence to the manuals using the NordLOTS Treatment Integrity Scale for FCBT and a corresponding manual developed by the TECTO research team for FPRT. Each manual included ratings of

(a) adherence to the manual (assessment of homework, exposure and response prevention/relaxation training, and parental involvement)

(b) therapist competence (ability to explain and implement the manual in session)

(c) relational skills

(d) treatment differentiation

Ratings were scored on a 4-point Likert scale ranging from 1 (“very poor competence”) to 4 (“very good competence”).

A total of eight therapists conducted the treatments. As patients were randomly assigned to either FCBT or FPRT and some patients did not consent to being video recorded, some therapists had fewer than the planned three available videos per treatment group resulting in a total of 40 therapy sessions (20 FCBT and 20 FPRT) available for fidelity rating.

The fidelity rating based on video recordings of 40 therapy sessions (20 FCBT and 20 FPRT) showed an overall good to very good manual compliance, competence, and differentiation, and all measures including patient difficulty showed no significant group differences for FCBT versus FPRT in ratings.

Table-S7 Comparison of mean fidelity ratings for 20 FCBT video-recordings and 20 FPRT video-recordings.

Comparisons by t-tests showed no significant differences in fidelity ratings between FCBT vs FPRT:

Item	Total group	FCBT n=20	FPRT n=20	FCBT vs FPRT *t*(degrees of freedom). p
Homework mean (SD)	3.45 (0.60)	3.50 (0.61)	3.40 (0.60)	*t*(38) = 0.53 . *p* = .60
Exercise	3.56 (0.55)	3.47 (0.61)	3.65 (0.49)	*t*(37) = -0.10 . *p* = .33
New homework	3.56 (0.50)	3.47 (0.51)	3.65 (0.49)	*t*(37) = -1.10. *p* = .28
Parental involvement	3.60 (0.55)	3.65 (0.49)	3.55 (0.61)	*t*(38) = 0.58 . *p* = .57
Total assessment	3.55 (0.51)	3.55 (0.51)	3.50 (0.51)	*t*(38) = 0.31 . *p* = .76
Competence administration	3.50 (0.51)	3.50 (0.51)	3.50 (0.51)	*t*(38) = 0.00. *p* = 1.00
Competence relations	3.55 (0.50)	3.55 (0.51)	3.55 (0.51)]	*t*(38) = 0.00. *p* = 1.00
Differentiation	3.73 (0.45)	3.80 (0.41)	3.65 (0.49)	*t*(38) = 1.05. *p* = .30

Item	Total group	FCBT n=20	FPRT n=20	FCBT vs FPRT *t*(degrees of freedom). p
Patient difficulty	3.13 (0.83)	3.26 (0.93)	3.00 (0.73)	*t*(37) = 0.99 . *p* = .33

Categorical comparison of fidelity rating. Pearson Chi-Square test showed no significant group differences between FCBT versus FPRT:

Item	Total group n=40	FCBT n=20	FPRT n=20	FCBT vs FPRT Pearson Chi-Square *t*(degrees of freedom). p
**Homework**				χ^2= .422
Compliance severely lacking, n	0	0	0	t(2)
Compliance lacking, n	2	1	1	p= .810
Good compliance, n	18	8	10	
Very good compliance, n	20	11	9	
Total, n	40	20	20	
Missing, n	0	0	0	
**Exercise**				χ^2= 1.433
Compliance severely lacking, n	0	0	0	t(2)
Compliance lacking, n	1	1	0	p= .488
Good compliance, n	15	8	7	
Very good compliance, n	23	10	13	
Total, n	39	19	20	
Missing, n	1	1	1	
**New homework**				χ^2=1.232
Compliance severely lacking, n	0	0	0	t(1)
Compliance lacking, n	0	0	0	p= .267
Good compliance, n	17	10	7	
Very good compliance, n	22	9	13	
Total, n	39	19	20	
Missing, n	1	1	0	
**Parental involvement**				χ^2= 1.040
Compliance severely lacking, n	0	0	0	t(2)
Compliance lacking, n	1	0	1	p= 0.595
Good compliance, n	14	7	7	
Very good compliance, n	25	13	12	
Total, n	40	20	40	
Missing, n	0	0	0	
**Total assessment**				χ^2= .100
Compliance severely lacking, n	0	0	0	t(1)
Compliance lacking, n	0	0	0	p= .752
Good compliance, n	19	9	10	
Very good compliance, n	21	11	10	
Total, n	40	20	20	
Missing, n	0	0	0	

Item	Total group n=40	FCBT n=20	FPRT n=20	FCBT vs FPRT Pearson Chi-Square *t*(degrees of freedom). p
**Competence administration**				χ^2= 0.000
Competence severely lacking, n	0	0	0	t(1)
Competence lacking, n	0	0	0	p=1.000
Good competence, n	20	10	10	
Very good competence, n	20	10	10	
Total, n	40	20	20	
Missing, n	0	0	0	
**Competence relations**				χ^2= 0.000
Competence severely lacking, n	0	0	0	t(1)
Competence lacking, n	0	0	0	p=1.000
Good competence, n	18	9	9	
Very good competence, n	22	11	11	
Total, n	40	20	20	
Missing, n	0	0	0	
**Differentiation**				χ^2= 1.129
Differentiation severely lacking, n	0	0	0	t(1)
Differentiation lacking, n	0	0	0	p= .288
Good differentiation, n	11	4	7	
Very good differentiation, n	29	16	13	
Total, n	40	20	20	
Missing, n	0	0	0	
**Patient difficulty**				χ^2= 4.811
Very difficult, n	1	1	0	t(3)
Difficult, n	8	3	5	p= .186
Not difficult, n	15	5	10	
Not difficult at all, n	15	10	5	
Total, n	39	19	20	
Missing, n	1	1	0	
