# Supplementary material for: Family-based cognitive behavioral therapy versus family-based psychoeducation and relaxation training for obsessive-compulsive disorder in children and adolescents: a randomized clinical trial (TECTO)
Source: Eur Child Adolesc Psychiatry. 2025 Jul 31;34(12):3955–71. doi: 10.1007/s00787-025-02797-4 (PMC12743107; doi:10.1007/s00787-025-02797-4)
Supplement: Supplementary file 2 — Supplementary Material 2 [file 787_2025_2797_MOESM2_ESM.pdf]

Tables from the TECTO-trial

Markus Harboe Olsen

12/3/23

Table of contents

Baseline table **4**

Outcome tables **9**

Gender and Sex agreement **10**

Posthoc interaction between sex and primary outcome **10**

Posthoc per protocol **11**

CY-BOCS effectsize and 95% confidence intervals **11**
Cohen's d | 95% CI 11

Figures **11**

Figure 2 - Psychopathology and family burden **12**

Figure 3 - Response status at 16 weeks **13**

Figure 4 - Negative effects questionnaire **14**

Posthoc nec per week **14**

Figure 5 - Raincloud **15**

Therapy compliance **16**
Reanalysis - Interaction analysis 16

Raw data **18**

Confidence in treatment **19**

Motivation for treatment **19**

Therapeutic alliance **20**

Kidscreen-52 **21**

Baseline table

	0	1	Overall
	(N=66)	(N=64)	(N=130)
**age__exact**			
Mean (SD)	13.4 (2.73)	13.1 (2.97)	13.3 (2.84)
Median [Min, Max]	13.6 [8.28, 18.0]	13.7 [8.07, 18.0]	13.7 [8.07, 18.0]
**gender**			
0	32 (48.5%)	20 (31.3%)	52 (40.0%)
1	21 (31.8%)	30 (46.9%)	51 (39.2%)
Missing	13 (19.7%)	14 (21.9%)	27 (20.8%)
**sex**			
Female	28 (42.4%)	40 (62.5%)	68 (52.3%)
Male	38 (57.6%)	24 (37.5%)	62 (47.7%)
**nationality**			
Danish	53 (80.3%)	53 (82.8%)	106 (81.5%)
Other	1 (1.5%)	2 (3.1%)	3 (2.3%)
Missing	12 (18.2%)	9 (14.1%)	21 (16.2%)
**nationality__p**			
Danish	50 (75.8%)	51 (79.7%)	101 (77.7%)
Danish and other	7 (10.6%)	6 (9.4%)	13 (10.0%)
Other	1 (1.5%)	3 (4.7%)	4 (3.1%)
Missing	8 (12.1%)	4 (6.3%)	12 (9.2%)
**COVID-19**			
Included after	44 (66.7%)	41 (64.1%)	85 (65.4%)
Included before	22 (33.3%)	23 (35.9%)	45 (34.6%)
**education**			
Mean (SD)	6.14 (1.01)	6.23 (1.10)	6.18 (1.05)
Median [Min, Max]	6.00 [3.00, 8.00]	6.00 [2.00, 8.00]	6.00 [2.00, 8.00]
Missing	9 (13.6%)	7 (10.9%)	16 (12.3%)
**IQ**			
Mean (SD)	99.4 (13.7)	97.8 (12.1)	98.6 (12.9)
Median [Min, Max]	99.5 [72.0, 132]	97.5 [70.0, 129]	98.0 [70.0, 132]
Missing	2 (3.0%)	0 (0%)	2 (1.5%)
**CYBOCS__b**			
Mean (SD)	25.8 (4.96)	25.8 (4.90)	25.8 (4.92)
Median [Min, Max]	25.0 [16.0, 35.0]	25.0 [16.0, 36.0]	25.0 [16.0, 36.0]
**KIDSCREEN__d__0**			
Mean (SD)	43.0 (6.13)	41.0 (6.46)	42.0 (6.34)
Median [Min, Max]	41.2 [31.9, 57.3]	40.7 [28.9, 59.8]	41.2 [28.9, 59.8]
Missing	13 (19.7%)	12 (18.8%)	25 (19.2%)
**COISR__d__0**			
Mean (SD)	28.2 (18.1)	26.2 (17.3)	27.3 (17.6)
Median [Min, Max]	28.0 [1.00, 70.0]	22.0 [3.00, 67.0]	27.0 [1.00, 70.0]
Missing	17 (25.8%)	20 (31.3%)	37 (28.5%)
**TOCS__d__0**			
Mean (SD)	11.2 (19.6)	10.1 (25.2)	10.7 (22.4)
Median [Min, Max]	11.0 [-54.0, 48.0]	17.0 [-56.0, 53.0]	14.0 [-56.0, 53.0]
Missing	15 (22.7%)	15 (23.4%)	30 (23.1%)
**CGIS__b**			
Mean (SD)	4.59 (0.830)	4.51 (0.878)	4.55 (0.852)
Median [Min, Max]	4.00 [3.00, 7.00]	4.00 [3.00, 7.00]	4.00 [3.00, 7.00]
Missing	2 (3.0%)	1 (1.6%)	3 (2.3%)
**CGAS__0**			
Mean (SD)	55.5 (9.54)	56.1 (9.66)	55.8 (9.57)
Median [Min, Max]	55.0 [39.0, 80.0]	55.0 [35.0, 75.0]	55.0 [35.0, 80.0]

	0	1	Overall
	(N=66)	(N=64)	(N=130)
**selvmordstanker_0**			
0	57 (86.4%)	54 (84.4%)	111 (85.4%)
1	5 (7.6%)	6 (9.4%)	11 (8.5%)
Missing	4 (6.1%)	4 (6.3%)	8 (6.2%)
**PSS_p_0**			
Mean (SD)	34.4 (7.55)	36.0 (7.89)	35.2 (7.72)
Median [Min, Max]	33.8 [19.5, 65.5]	37.0 [19.0, 56.5]	35.0 [19.0, 65.5]
Missing	8 (12.1%)	12 (18.8%)	20 (15.4%)
**FASPR_p_0**			
Mean (SD)	19.5 (14.1)	18.1 (15.7)	18.9 (14.8)
Median [Min, Max]	16.0 [1.00, 62.0]	14.0 [0, 66.5]	15.0 [0, 66.5]
Missing	8 (12.1%)	11 (17.2%)	19 (14.6%)
**subtype**			
Mixed obsessional thoughts and acts	64 (97.0%)	63 (98.4%)	127 (97.7%)
Predominantly compulsive acts	2 (3.0%)	0 (0%)	2 (1.5%)
Predominantly obsessional thoughts or ruminations	0 (0%)	1 (1.6%)	1 (0.8%)
**Depressive**			
0	59 (89.4%)	62 (96.9%)	121 (93.1%)
1	7 (10.6%)	2 (3.1%)	9 (6.9%)
**Developmental**			
0	56 (84.8%)	57 (89.1%)	113 (86.9%)
1	10 (15.2%)	7 (10.9%)	17 (13.1%)
**Anxiety**			
0	56 (84.8%)	57 (89.1%)	113 (86.9%)
1	10 (15.2%)	7 (10.9%)	17 (13.1%)
**Eating**			
0	65 (98.5%)	60 (93.8%)	125 (96.2%)
1	1 (1.5%)	4 (6.3%)	5 (3.8%)
**Personality**			
0	66 (100%)	63 (98.4%)	129 (99.2%)
1	0 (0%)	1 (1.6%)	1 (0.8%)
**Hyperkinetic**			
0	57 (86.4%)	58 (90.6%)	115 (88.5%)
1	9 (13.6%)	6 (9.4%)	15 (11.5%)
**Conduct**			
0	66 (100%)	63 (98.4%)	129 (99.2%)
1	0 (0%)	1 (1.6%)	1 (0.8%)
**Tics**			
0	57 (86.4%)	57 (89.1%)	114 (87.7%)
1	9 (13.6%)	7 (10.9%)	16 (12.3%)
**srs_p_0**			
Mean (SD)	52.9 (8.10)	52.7 (7.99)	52.8 (8.01)
Median [Min, Max]	53.0 [39.0, 75.0]	50.0 [41.5, 70.5]	51.3 [39.0, 75.0]
Missing	2 (3.0%)	4 (6.3%)	6 (4.6%)

	0	1	Overall
	(N=66)	(N=64)	(N=130)
**Emotional**			
0	62 (93.9%)	62 (96.9%)	124 (95.4%)
1	4 (6.1%)	2 (3.1%)	6 (4.6%)
**Sleeping**			
0	66 (100%)	63 (98.4%)	129 (99.2%)
1	0 (0%)	1 (1.6%)	1 (0.8%)
**Enuresis**			
0	65 (98.5%)	60 (93.8%)	125 (96.2%)
1	1 (1.5%)	4 (6.3%)	5 (3.8%)
**Attachment**			
0	65 (98.5%)	64 (100%)	129 (99.2%)
1	1 (1.5%)	0 (0%)	1 (0.8%)

	0	1	Overall
	(N=66)	(N=64)	(N=130)
**FES.D__Cohesion**			
Mean (SD)	6.70 (1.69)	6.73 (1.73)	6.71 (1.70)
Median [Min, Max]	7.00 [0, 9.00]	7.00 [1.00, 9.00]	7.00 [0, 9.00]
Missing	16 (24.2%)	20 (31.3%)	36 (27.7%)
**FES.D__Expressiveness**			
Mean (SD)	5.63 (1.66)	5.44 (1.53)	5.54 (1.60)
Median [Min, Max]	6.00 [1.00, 8.00]	5.00 [2.00, 8.00]	6.00 [1.00, 8.00]
Missing	15 (22.7%)	19 (29.7%)	34 (26.2%)
**FES.D__Conflict**			
Mean (SD)	2.59 (2.29)	2.48 (2.18)	2.54 (2.23)
Median [Min, Max]	2.00 [0, 8.00]	2.00 [0, 8.00]	2.00 [0, 8.00]
Missing	15 (22.7%)	20 (31.3%)	35 (26.9%)
**FES.D__Independence**			
Mean (SD)	5.55 (1.49)	5.56 (1.57)	5.55 (1.52)
Median [Min, Max]	5.00 [3.00, 9.00]	6.00 [1.00, 8.00]	6.00 [1.00, 9.00]
Missing	15 (22.7%)	19 (29.7%)	34 (26.2%)
**FES.D__Achievement__Orientation**			
Mean (SD)	3.20 (1.89)	3.55 (2.04)	3.36 (1.96)
Median [Min, Max]	3.00 [0, 7.00]	3.50 [0, 8.00]	3.00 [0, 8.00]
Missing	15 (22.7%)	20 (31.3%)	35 (26.9%)
**FES.D__Intellectual__Cultural__Orientation**			
Mean (SD)	4.53 (2.18)	3.49 (1.89)	4.04 (2.10)
Median [Min, Max]	4.00 [0, 8.00]	3.00 [0, 8.00]	4.00 [0, 8.00]
Missing	15 (22.7%)	19 (29.7%)	34 (26.2%)
**FES.D__Active__Recreational__Orientation**			
Mean (SD)	4.48 (2.12)	4.53 (2.03)	4.51 (2.07)
Median [Min, Max]	4.00 [0, 8.00]	4.00 [0, 8.00]	4.00 [0, 8.00]
Missing	16 (24.2%)	19 (29.7%)	35 (26.9%)
**FES.D__Moral__Religious__Emphasis**			
Mean (SD)	1.88 (1.36)	1.98 (1.51)	1.93 (1.43)
Median [Min, Max]	2.00 [0, 7.00]	2.00 [0, 7.00]	2.00 [0, 7.00]
Missing	17 (25.8%)	19 (29.7%)	36 (27.7%)
**FES.D__Organization**			
Mean (SD)	5.73 (1.71)	5.96 (1.61)	5.84 (1.65)
Median [Min, Max]	6.00 [2.00, 9.00]	6.00 [1.00, 9.00]	6.00 [1.00, 9.00]
Missing	17 (25.8%)	19 (29.7%)	36 (27.7%)
**FES.D__Control**			
Mean (SD)	3.06 (1.99)	3.44 (1.82)	3.24 (1.91)
Median [Min, Max]	3.00 [0, 9.00]	4.00 [0, 8.00]	3.00 [0, 9.00]
Missing	17 (25.8%)	19 (29.7%)	36 (27.7%)

	0	1	Overall
	(N=66)	(N=64)	(N=130)
**FES.P__Cohesion**			
Mean (SD)	6.91 (1.51)	7.04 (1.55)	6.97 (1.53)
Median [Min, Max]	7.00 [1.50, 9.00]	7.50 [1.00, 9.00]	7.00 [1.00, 9.00]
Missing	8 (12.1%)	10 (15.6%)	18 (13.8%)
**FES.P__Expressiveness**			
Mean (SD)	6.28 (1.53)	6.54 (1.41)	6.41 (1.47)
Median [Min, Max]	6.50 [2.50, 9.00]	6.50 [2.50, 9.00]	6.50 [2.50, 9.00]
Missing	9 (13.6%)	10 (15.6%)	19 (14.6%)
**FES.P__Conflict**			
Mean (SD)	2.63 (1.98)	2.81 (1.66)	2.72 (1.83)
Median [Min, Max]	2.25 [0, 7.50]	2.50 [0, 6.50]	2.50 [0, 7.50]
Missing	8 (12.1%)	10 (15.6%)	18 (13.8%)
**FES.P__Independence**			
Mean (SD)	6.11 (1.56)	5.99 (1.44)	6.05 (1.49)
Median [Min, Max]	6.00 [3.00, 9.00]	6.00 [2.50, 9.00]	6.00 [2.50, 9.00]
Missing	9 (13.6%)	10 (15.6%)	19 (14.6%)
**FES.P__Achievement__Orientation**			
Mean (SD)	3.24 (1.58)	3.06 (1.75)	3.16 (1.66)
Median [Min, Max]	3.00 [0, 7.00]	3.00 [0, 7.00]	3.00 [0, 7.00]
Missing	8 (12.1%)	10 (15.6%)	18 (13.8%)
**FES.P__Intellectual__Cultural__Orientation**			
Mean (SD)	4.85 (2.04)	4.41 (1.91)	4.64 (1.98)
Median [Min, Max]	4.75 [0, 9.00]	4.50 [0.500, 9.00]	4.50 [0, 9.00]
Missing	8 (12.1%)	10 (15.6%)	18 (13.8%)
**FES.P__Active__Recreational__Orientation**			
Mean (SD)	4.32 (1.95)	4.44 (1.72)	4.38 (1.83)
Median [Min, Max]	4.00 [0, 9.00]	4.50 [0, 9.00]	4.50 [0, 9.00]
Missing	9 (13.6%)	10 (15.6%)	19 (14.6%)
**FES.P__Moral__Religious__Emphasis**			
Mean (SD)	1.72 (1.50)	1.80 (1.27)	1.76 (1.38)
Median [Min, Max]	1.50 [0, 8.00]	1.50 [0, 5.00]	1.50 [0, 8.00]
Missing	9 (13.6%)	10 (15.6%)	19 (14.6%)
**FES.P__Organization**			
Mean (SD)	6.10 (1.49)	6.52 (1.27)	6.30 (1.40)
Median [Min, Max]	6.00 [3.50, 9.00]	6.50 [3.00, 8.50]	6.50 [3.00, 9.00]
Missing	8 (12.1%)	10 (15.6%)	18 (13.8%)
**FES.P__Control**			
Mean (SD)	3.48 (1.72)	3.48 (1.50)	3.48 (1.61)
Median [Min, Max]	3.50 [0, 7.50]	3.50 [1.00, 7.50]	3.50 [0, 7.50]
Missing	8 (12.1%)	10 (15.6%)	18 (13.8%)

	0	1	Overall
	(N=66)	(N=64)	(N=130)
**numberofcomorb**			
0	36 (54.5%)	36 (56.3%)	72 (55.4%)
1	13 (19.7%)	17 (26.6%)	30 (23.1%)
2	15 (22.7%)	8 (12.5%)	23 (17.7%)
3	0 (0%)	3 (4.7%)	3 (2.3%)
4	1 (1.5%)	0 (0%)	1 (0.8%)
5	1 (1.5%)	0 (0%)	1 (0.8%)

	0	1	Overall
	(N=66)	(N=64)	(N=130)
**as.factor(education)**			
2	0 (0%)	1 (1.6%)	1 (0.8%)
3	2 (3.0%)	1 (1.6%)	3 (2.3%)
5	11 (16.7%)	10 (15.6%)	21 (16.2%)
6	21 (31.8%)	18 (28.1%)	39 (30.0%)
7	21 (31.8%)	24 (37.5%)	45 (34.6%)
8	2 (3.0%)	3 (4.7%)	5 (3.8%)
Missing	9 (13.6%)	7 (10.9%)	16 (12.3%)

Outcome tables

	0	1	Overall
	(N=66)	(N=64)	(N=130)
**CYBOCS_b**			
Mean (SD)	25.8 (4.96)	25.8 (4.90)	25.8 (4.92)
Median [Min, Max]	25.0 [16.0, 35.0]	25.0 [16.0, 36.0]	25.0 [16.0, 36.0]
**CYBOCS_4**			
Mean (SD)	24.3 (5.23)	23.0 (6.18)	23.7 (5.72)
Median [Min, Max]	24.5 [12.0, 36.0]	22.0 [7.00, 36.0]	23.0 [7.00, 36.0]
Missing	4 (6.1%)	7 (10.9%)	11 (8.5%)
**CYBOCS_8**			
Mean (SD)	22.9 (5.34)	20.1 (5.92)	21.6 (5.79)
Median [Min, Max]	23.0 [9.00, 34.0]	20.0 [3.00, 33.0]	22.0 [3.00, 34.0]
Missing	7 (10.6%)	7 (10.9%)	14 (10.8%)
**CYBOCS_16**			
Mean (SD)	19.9 (8.09)	15.9 (8.68)	17.8 (8.60)
Median [Min, Max]	20.5 [3.00, 35.0]	16.0 [0, 39.0]	18.0 [0, 39.0]
Missing	14 (21.2%)	5 (7.8%)	19 (14.6%)

	0	1	Overall
	(N=66)	(N=64)	(N=130)
**KIDSCREEN_d_0**			
Mean (SD)	43.0 (6.13)	41.0 (6.46)	42.0 (6.34)
Median [Min, Max]	41.2 [31.9, 57.3]	40.7 [28.9, 59.8]	41.2 [28.9, 59.8]
Missing	13 (19.7%)	12 (18.8%)	25 (19.2%)
**KIDSCREEN_d_4**			
Mean (SD)	41.6 (6.81)	43.0 (7.29)	42.3 (7.07)
Median [Min, Max]	41.2 [28.9, 59.8]	42.3 [30.0, 62.9]	42.3 [28.9, 62.9]
Missing	25 (37.9%)	17 (26.6%)	42 (32.3%)
**KIDSCREEN_d_8**			
Mean (SD)	39.1 (5.16)	41.3 (5.19)	40.3 (5.27)
Median [Min, Max]	38.8 [28.9, 49.8]	42.3 [31.9, 51.4]	40.2 [28.9, 51.4]
Missing	30 (45.5%)	21 (32.8%)	51 (39.2%)
**KIDSCREEN_d_16**			
Mean (SD)	36.4 (4.53)	37.3 (5.04)	36.9 (4.81)
Median [Min, Max]	37.0 [24.0, 45.7]	36.5 [22.4, 45.7]	36.5 [22.4, 45.7]
Missing	36 (54.5%)	23 (35.9%)	59 (45.4%)

	0	1	Overall
	(N=66)	(N=64)	(N=130)
**NEQ_d_4**			
Mean (SD)	5.04 (3.90)	3.61 (3.02)	4.38 (3.58)
Median [Min, Max]	4.00 [0, 14.0]	3.50 [0, 12.0]	4.00 [0, 14.0]
Missing	15 (22.7%)	20 (31.3%)	35 (26.9%)
**NEQ_d_8**			
Mean (SD)	4.85 (3.84)	3.85 (3.91)	4.36 (3.89)
Median [Min, Max]	3.00 [0, 15.0]	3.00 [0, 16.0]	3.00 [0, 16.0]
Missing	18 (27.3%)	17 (26.6%)	35 (26.9%)
**NEQ_d_16**			
Mean (SD)	3.87 (3.68)	3.17 (2.89)	3.48 (3.27)
Median [Min, Max]	3.00 [0, 14.0]	3.00 [0, 13.0]	3.00 [0, 14.0]
Missing	28 (42.4%)	17 (26.6%)	45 (34.6%)

Gender and Sex agreement

[1] 0 Missing data på 27 selvrapporterede gender, men alle rapporterede stemte overens med sex!

Posthoc interaction between sex and primary outcome

	CYBOCS_16		
Predictors	Estimates	CI	p
(Intercept)	0.43	-8.66 – 9.52	0.926

	CYBOCS_16		
allocation [1]	-3.40	-7.41 – 0.62	0.096
strata age [1]	3.57	0.47 – 6.66	**0.024**
CYBOCS b	0.65	0.33 – 0.96	**<0.001**
male [1]	2.48	-7.11 – 12.07	0.609
allocation * male [1]	-0.62	-6.61 – 5.38	0.839
Observations	111		
R^2 / R^2 adjusted	0.212 / 0.174		

Posthoc per protocol

	CYBOCS_16		
Predictors	Estimates	CI	p
(Intercept)	2.32	-6.33 – 10.97	0.596
allocation [1]	-2.59	-5.66 – 0.49	0.098
strata age [1]	4.15	1.09 – 7.21	**0.008**
CYBOCS b	0.54	0.21 – 0.87	**0.002**
Observations	93		
R^2 / R^2 adjusted	0.186 / 0.159		

CY-BOCS effectsize and 95% confidence intervals

95% Confidence intervals:Group 0: 19.9 ; 95%CI: 17.65 - 22.16Group 1: 15.95 ; 95%CI: 13.69 - 18.21Effect size:

Cohen's d | 95% CI

0.47 | [0.09, 0.85]

- Estimated using pooled SD.

Figures

Figure 2 - Psychopathology and family burden

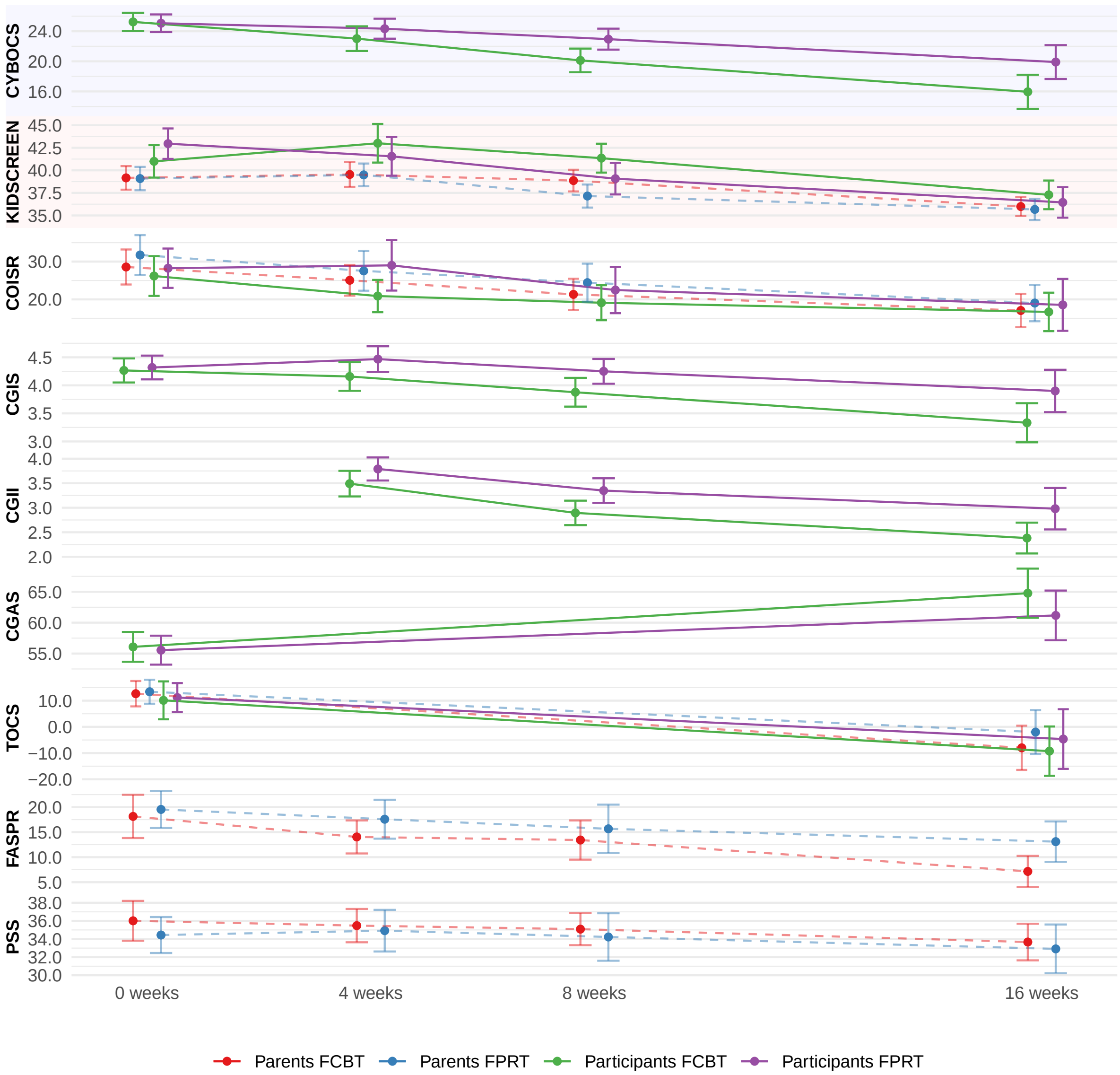

CY-BOCS: Children's Yale–Brown Obsessive Compulsive Scale; COIS-R: Child Obsessive-Compulsive Impact Scale-Revised; CGI-S: Clinical Global Impressions Severity; CGI-I: Clinical Global Impressions Improvement; Children's Global Assessment Scale; TOCS: Toronto Obsessive-Compulsive Scale; FAS-PR: Family Accommodation Scale for Obsessive-Compulsive Disorder; PSS: Parental stress scale.

Figure 3 - Response status at 16 weeks

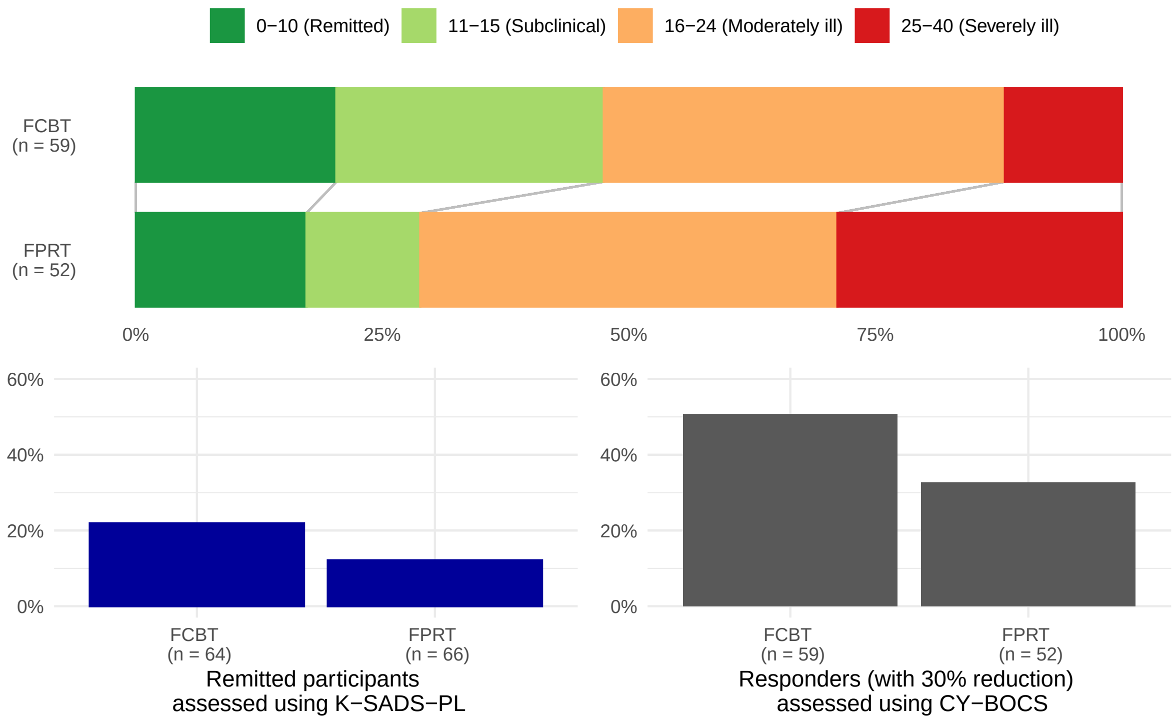

	0	1	Overall
	(N=66)	(N=64)	(N=130)
**CB_group**			
0-10 (Remitted)	9 (13.6%)	12 (18.8%)	21 (16.2%)
11-15 (Subclinical)	6 (9.1%)	16 (25.0%)	22 (16.9%)
16-24 (Moderately ill)	22 (33.3%)	24 (37.5%)	46 (35.4%)
25-40 (Severely ill)	15 (22.7%)	7 (10.9%)	22 (16.9%)
Missing	14 (21.2%)	5 (7.8%)	19 (14.6%)

Figure 4 - Negative effects questionnaire

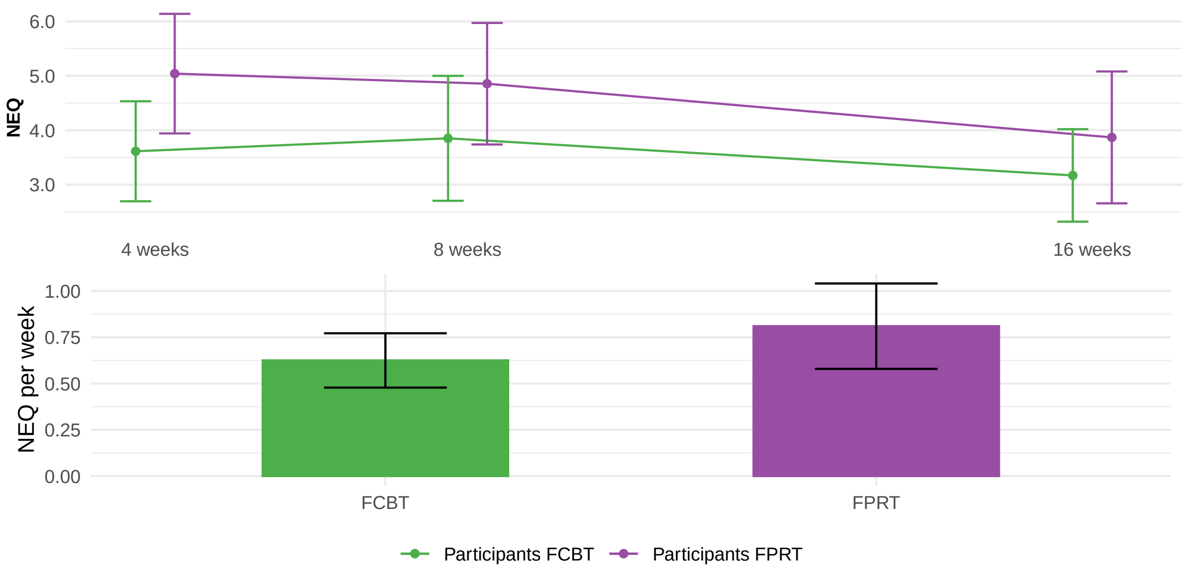

Posthoc nec per week

	0	1	Overall
	(N=66)	(N=64)	(N=130)
**Negative effect burden**			
Mean (SD)	0.810 (0.595)	0.625 (0.434)	0.706 (0.515)
Median [Min, Max]	0.625 [0.0625, 2.63]	0.563 [0, 1.69]	0.594 [0, 2.63]
Missing	38 (57.6%)	28 (43.8%)	66 (50.8%)

	Negative effect burden		
Predictors	Estimates	CI	p
(Intercept)	0.59	0.33 – 0.85	**<0.001**
allocation [1]	-0.20	-0.44 – 0.05	0.114
strata age [1]	0.36	0.11 – 0.61	**0.006**
strata cybocs [1]	0.01	-0.24 – 0.25	0.966
Observations	64		
R^2 / R^2 adjusted	0.149 / 0.107		

Figure 5 - Raincloud

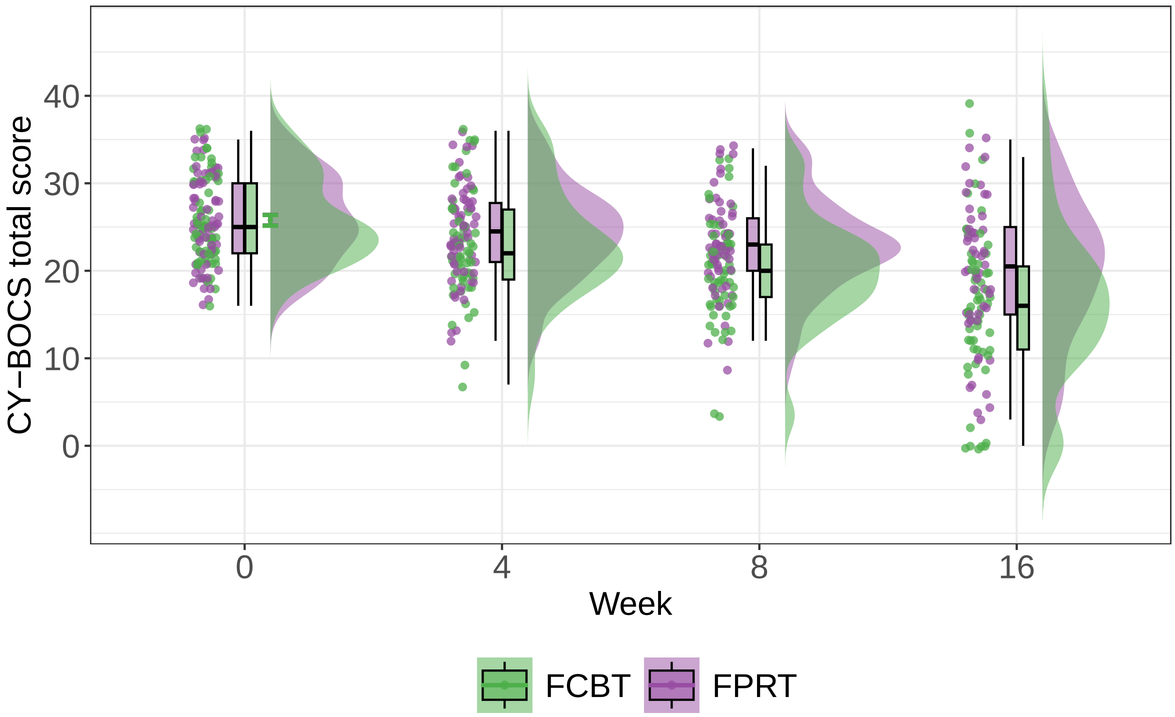

Therapy compliance

Reanalysis - Interaction analysis

	CYBOCS_16		
Predictors	Estimates	CI	p
(Intercept)	10.71	-4.48 – 25.89	0.165
allocation [1]	10.75	-5.72 – 27.21	0.198
strata age [1]	2.97	0.30 – 5.64	**0.030**
CYBOCS b	0.68	0.40 – 0.97	**<0.001**
moderator compliance	0.94	-4.33 – 6.21	0.725
allocation * moderator compliance	-2.83	-5.93 – 0.27	0.073
Observations	110		
R^2 / R^2 adjusted	0.369 / 0.338		

Raw data

	0	1	Overall
	(N=66)	(N=64)	(N=130)
**S10__Compliance**			
Mean (SD)	4.95 (1.41)	5.15 (1.27)	5.05 (1.34)
Median [Min, Max]	5.00 [1.00, 7.00]	5.00 [2.00, 7.00]	5.00 [1.00, 7.00]
Missing	29 (43.9%)	25 (39.1%)	54 (41.5%)
**S11__Compliance**			
Mean (SD)	5.35 (1.06)	4.88 (1.59)	5.10 (1.38)
Median [Min, Max]	6.00 [3.00, 7.00]	5.00 [2.00, 7.00]	5.50 [2.00, 7.00]
Missing	29 (43.9%)	21 (32.8%)	50 (38.5%)
**S12__Compliance**			
Mean (SD)	4.93 (1.70)	5.17 (1.61)	5.07 (1.64)
Median [Min, Max]	5.50 [1.00, 7.00]	6.00 [1.00, 7.00]	6.00 [1.00, 7.00]
Missing	36 (54.5%)	22 (34.4%)	58 (44.6%)
**S13__Compliance**			
Mean (SD)	5.23 (1.19)	5.42 (1.54)	5.35 (1.41)
Median [Min, Max]	5.00 [3.00, 7.00]	6.00 [1.00, 7.00]	6.00 [1.00, 7.00]
Missing	44 (66.7%)	26 (40.6%)	70 (53.8%)
**S14__Compliance**			
Mean (SD)	5.47 (1.25)	5.10 (1.48)	5.26 (1.39)
Median [Min, Max]	6.00 [2.00, 7.00]	5.00 [1.00, 7.00]	6.00 [1.00, 7.00]
Missing	36 (54.5%)	25 (39.1%)	61 (46.9%)
**S2__Compliance**			
Mean (SD)	5.15 (1.44)	5.30 (1.16)	5.22 (1.31)
Median [Min, Max]	6.00 [1.00, 7.00]	6.00 [2.00, 7.00]	6.00 [1.00, 7.00]
Missing	6 (9.1%)	7 (10.9%)	13 (10.0%)
**S3__Compliance**			
Mean (SD)	5.16 (1.42)	5.05 (1.61)	5.11 (1.51)
Median [Min, Max]	6.00 [1.00, 7.00]	6.00 [1.00, 7.00]	6.00 [1.00, 7.00]
Missing	5 (7.6%)	6 (9.4%)	11 (8.5%)
**S4__Compliance**			
Mean (SD)	5.13 (1.63)	5.16 (1.71)	5.15 (1.66)
Median [Min, Max]	6.00 [1.00, 7.00]	6.00 [1.00, 7.00]	6.00 [1.00, 7.00]
Missing	4 (6.1%)	9 (14.1%)	13 (10.0%)
**S5__Compliance**			
Mean (SD)	5.28 (1.36)	5.47 (1.36)	5.37 (1.36)
Median [Min, Max]	6.00 [1.00, 7.00]	6.00 [1.00, 7.00]	6.00 [1.00, 7.00]
Missing	8 (12.1%)	13 (20.3%)	21 (16.2%)
**S6__Compliance**			
Mean (SD)	4.91 (1.57)	4.90 (1.64)	4.91 (1.60)
Median [Min, Max]	6.00 [1.00, 7.00]	5.00 [1.00, 7.00]	5.00 [1.00, 7.00]
Missing	9 (13.6%)	12 (18.8%)	21 (16.2%)
**S7__Compliance**			
Mean (SD)	5.21 (1.30)	5.47 (1.52)	5.33 (1.40)
Median [Min, Max]	6.00 [1.00, 7.00]	6.00 [1.00, 7.00]	6.00 [1.00, 7.00]
Missing	10 (15.2%)	17 (26.6%)	27 (20.8%)
**S8__Compliance**			
Mean (SD)	5.14 (1.24)	5.35 (1.31)	5.26 (1.28)
Median [Min, Max]	6.00 [2.00, 7.00]	6.00 [2.00, 7.00]	6.00 [2.00, 7.00]
Missing	24 (36.4%)	13 (20.3%)	37 (28.5%)
**S9__Compliance**			
Mean (SD)	5.16 (1.44)	5.47 (1.73)	5.33 (1.61)
Median [Min, Max]	6.00 [1.00, 7.00]	6.00 [1.00, 7.00]	6.00 [1.00, 7.00]
Missing	28 (42.4%)	17 (26.6%)	45 (34.6%)

Confidence in treatment

	0	1	Overall
	(N=66)	(N=64)	(N=130)
**moderator__confidence**			
Mean (SD)	4.53 (0.903)	5.50 (1.15)	4.97 (1.13)
Median [Min, Max]	5.00 [3.00, 6.00]	5.50 [3.00, 7.00]	5.00 [3.00, 7.00]
Missing	8 (12.1%)	16 (25.0%)	24 (18.5%)

Motivation for treatment

	0	1	Overall
	(N=66)	(N=64)	(N=130)
**Motivation__b**			
Mean (SD)	5.62 (1.69)	5.16 (1.59)	5.40 (1.65)
Median [Min, Max]	6.00 [1.00, 7.00]	5.00 [1.00, 7.00]	6.00 [1.00, 7.00]
Missing	14 (21.2%)	15 (23.4%)	29 (22.3%)
**Motivation__1**			
Mean (SD)	5.62 (1.28)	5.45 (1.76)	5.54 (1.52)
Median [Min, Max]	6.00 [2.00, 7.00]	6.00 [1.00, 7.00]	6.00 [1.00, 7.00]
Missing	19 (28.8%)	20 (31.3%)	39 (30.0%)
**Motivation__4**			
Mean (SD)	5.05 (1.67)	5.53 (1.62)	5.30 (1.65)
Median [Min, Max]	5.00 [2.00, 7.00]	6.00 [1.00, 7.00]	6.00 [1.00, 7.00]
Missing	24 (36.4%)	19 (29.7%)	43 (33.1%)
**Motivation__8**			
Mean (SD)	5.00 (1.52)	5.65 (1.48)	5.36 (1.52)
Median [Min, Max]	5.00 [2.00, 7.00]	6.00 [1.00, 7.00]	6.00 [1.00, 7.00]
Missing	32 (48.5%)	21 (32.8%)	53 (40.8%)
**Motivation__14**			
Mean (SD)	5.45 (1.23)	5.74 (1.44)	5.60 (1.34)
Median [Min, Max]	6.00 [3.00, 7.00]	6.00 [1.00, 7.00]	6.00 [1.00, 7.00]
Missing	35 (53.0%)	30 (46.9%)	65 (50.0%)

Therapeutic alliance

	0	1	Overall
	(N=66)	(N=64)	(N=130)
**Alliance_1**			
Mean (SD)	29.5 (2.89)	30.6 (2.50)	30.0 (2.75)
Median [Min, Max]	30.0 [23.0, 35.0]	31.0 [25.0, 35.0]	30.0 [23.0, 35.0]
Missing	27 (40.9%)	31 (48.4%)	58 (44.6%)
**Alliance_4**			
Mean (SD)	29.7 (3.26)	30.8 (4.16)	30.3 (3.77)
Median [Min, Max]	29.0 [22.0, 39.0]	31.0 [20.0, 42.0]	30.0 [20.0, 42.0]
Missing	24 (36.4%)	20 (31.3%)	44 (33.8%)
**Alliance_8**			
Mean (SD)	29.8 (2.75)	30.7 (3.57)	30.3 (3.26)
Median [Min, Max]	30.5 [25.0, 36.0]	31.0 [22.0, 37.0]	31.0 [22.0, 37.0]
Missing	34 (51.5%)	21 (32.8%)	55 (42.3%)
**Alliance_16**			
Mean (SD)	30.4 (3.33)	31.2 (4.32)	30.8 (3.90)
Median [Min, Max]	31.0 [20.0, 37.0]	32.0 [22.0, 41.0]	31.0 [20.0, 41.0]
Missing	35 (53.0%)	26 (40.6%)	61 (46.9%)

Kidscreen-52

	0<br>*n = 66*	1<br>*n = 64*	Linear regression<br>*estimate (95%CI)*	*p*
d_0_k52phy				
mean (95%CI)	47.29 (43.77;50.80)	49.47 (46.58;52.36)		
median (min, max)	45.91 (24.00, 73.20)	49.63 (30.57, 73.20)		
missing	12 (18.2%)	12 (18.8%)		
d_4_k52phy				
mean (95%CI)	45.85 (42.39;49.31)	46.54 (42.48;50.60)		
median (min, max)	47.08 (24.00, 73.20)	44.73 (24.00, 73.20)		
missing	25 (37.9%)	16 (25%)		
d_8_k52phy				
mean (95%CI)	49.21 (45.02;53.40)	51.04 (46.72;55.35)		
median (min, max)	49.63 (24.00, 73.20)	51.03 (24.00, 73.20)		
missing	29 (43.9%)	20 (31.2%)		
d_16_k52phy			-3.41 (-10.61;3.78)	0.346
mean (95%CI)	48.49 (43.99;53.00)	46.27 (41.31;51.23)		
median (min, max)	49.63 (24.00, 73.20)	47.08 (24.00, 73.20)		
missing	34 (51.5%)	22 (34.4%)		

	0<br>*n = 66*	1<br>*n = 64*	Linear regression<br>*estimate (95%CI)*	*p*
p_0_k52phy				
mean (95%CI)	42.08 (39.60;44.56)	44.99 (42.38;47.60)		
median (min, max)	40.07 (24.00, 68.75)	44.77 (20.70, 73.20)		
missing	6 (9.1%)	8 (12.5%)		
p_4_k52phy				
mean (95%CI)	44.88 (42.24;47.51)	46.48 (44.12;48.85)		
median (min, max)	44.73 (28.13, 64.30)	45.91 (20.70, 73.20)		
missing	15 (22.7%)	13 (20.3%)		
p_8_k52phy				
mean (95%CI)	45.16 (42.54;47.79)	47.32 (44.60;50.04)		
median (min, max)	43.76 (25.07, 73.20)	48.36 (24.00, 73.20)		
missing	16 (24.2%)	11 (17.2%)		
p_16_k52phy			0.48 (-3.38;4.35)	0.804
mean (95%CI)	47.28 (44.40;50.15)	48.85 (46.02;51.68)		
median (min, max)	45.47 (24.50, 73.20)	47.33 (32.69, 73.20)		
missing	24 (36.4%)	20 (31.2%)		

	0	1	Linear regression	
	n = 66	*n = 64*	*estimate (95%CI)*	*p*
d_0_k52pwb				
mean (95%CI)	44.25 (41.42;47.08)	42.44 (39.70;45.18)		
median (min, max)	41.53 (23.07, 68.49)	43.25 (25.23, 68.49)		
missing	13 (19.7%)	12 (18.8%)		
d_4_k52pwb				
mean (95%CI)	43.20 (40.22;46.18)	45.25 (42.52;47.97)		
median (min, max)	43.25 (25.23, 68.49)	45.10 (27.04, 68.49)		
missing	24 (36.4%)	15 (23.4%)		
d_8_k52pwb				
mean (95%CI)	44.86 (42.00;47.72)	48.39 (45.40;51.38)		
median (min, max)	45.10 (28.63, 68.49)	47.12 (25.23, 68.49)		
missing	29 (43.9%)	20 (31.2%)		
d_16_k52pwb			3.15 (-1.78;8.09)	0.207
mean (95%CI)	47.20 (43.79;50.61)	49.79 (46.15;53.44)		
median (min, max)	45.10 (35.50, 68.49)	47.12 (31.45, 68.49)		
missing	34 (51.5%)	22 (34.4%)		

	0	1	Linear regression	
	n = 66	*n = 64*	*estimate (95%CI)*	*p*
p_0_k52pwb				
mean (95%CI)	38.53 (36.66;40.40)	38.47 (36.35;40.60)		
median (min, max)	36.91 (27.84, 61.49)	38.00 (23.07, 65.02)		
missing	6 (9.1%)	8 (12.5%)		
p_4_k52pwb				
mean (95%CI)	38.42 (36.46;40.38)	39.90 (37.96;41.84)		
median (min, max)	37.64 (26.58, 54.49)	40.48 (16.65, 54.49)		
missing	15 (22.7%)	14 (21.9%)		
p_8_k52pwb				
mean (95%CI)	40.24 (38.15;42.33)	42.59 (40.59;44.60)		
median (min, max)	39.91 (25.23, 61.49)	42.39 (23.07, 65.02)		
missing	16 (24.2%)	11 (17.2%)		
p_16_k52pwb			2.71 (-0.01;5.43)	0.050
mean (95%CI)	42.51 (40.30;44.72)	44.67 (42.28;47.06)		
median (min, max)	42.26 (30.76, 61.49)	44.33 (27.66, 68.49)		
missing	24 (36.4%)	20 (31.2%)		

	0	1	Linear regression	
	n = 66	*n = 64*	*estimate (95%CI)*	*p*
d_0_k52emo				
mean (95%CI)	44.67 (42.23;47.10)	44.60 (41.92;47.29)		
median (min, max)	43.20 (29.04, 70.91)	43.91 (32.51, 70.91)		
missing	12 (18.2%)	12 (18.8%)		
d_4_k52emo				
mean (95%CI)	45.48 (42.12;48.85)	46.86 (44.08;49.65)		
median (min, max)	46.30 (31.42, 70.91)	47.15 (31.42, 70.91)		
missing	24 (36.4%)	16 (25%)		
d_8_k52emo				
mean (95%CI)	45.27 (42.07;48.48)	50.04 (47.55;52.52)		
median (min, max)	43.91 (29.04, 70.91)	51.34 (36.70, 70.91)		
missing	29 (43.9%)	20 (31.2%)		
d_16_k52emo			0.53 (-3.92;4.98)	0.812
mean (95%CI)	47.12 (43.22;51.01)	49.51 (45.81;53.21)		
median (min, max)	45.44 (31.42, 70.91)	47.15 (29.04, 70.91)		
missing	34 (51.5%)	23 (35.9%)		

	0	1	Linear regression	
	n = 66	*n = 64*	*estimate (95%CI)*	*p*
p_0_k52emo				
mean (95%CI)	41.35 (39.62;43.09)	41.02 (39.23;42.82)		
median (min, max)	39.24 (32.44, 62.06)	41.25 (29.04, 62.06)		
missing	6 (9.1%)	8 (12.5%)		
p_4_k52emo				
mean (95%CI)	42.40 (40.30;44.50)	43.35 (41.30;45.39)		
median (min, max)	41.25 (29.06, 58.04)	42.15 (29.04, 64.16)		
missing	15 (22.7%)	13 (20.3%)		
p_8_k52emo				
mean (95%CI)	44.46 (42.12;46.80)	46.35 (44.21;48.49)		
median (min, max)	43.65 (31.42, 62.47)	44.67 (30.27, 62.47)		
missing	16 (24.2%)	11 (17.2%)		
p_16_k52emo			1.23 (-1.79;4.25)	0.421
mean (95%CI)	46.83 (44.34;49.32)	47.61 (45.06;50.15)		
median (min, max)	46.72 (32.34, 66.48)	47.44 (32.51, 62.06)		
missing	24 (36.4%)	20 (31.2%)		

	0<br>*n = 66*	1<br>*n = 64*	Linear regression<br>*estimate (95%CI)*	*p*
d__0__k52sel				
mean (95%CI)	47.85 (45.13;50.56)	46.52 (43.85;49.20)		
median (min, max)	46.09 (33.20, 69.78)	46.09 (28.88, 69.78)		
missing	13 (19.7%)	12 (18.8%)		
d__4__k52sel				
mean (95%CI)	47.81 (44.56;51.06)	49.51 (46.56;52.46)		
median (min, max)	45.34 (25.83, 69.78)	47.78 (31.24, 69.78)		
missing	24 (36.4%)	16 (25%)		
d__8__k52sel				
mean (95%CI)	48.43 (44.82;52.05)	50.45 (46.78;54.12)		
median (min, max)	46.09 (28.88, 69.78)	47.78 (25.83, 69.78)		
missing	29 (43.9%)	20 (31.2%)		
d__16__k52sel			-0.47 (-4.06;3.13)	0.795
mean (95%CI)	48.43 (45.48;51.37)	49.43 (45.69;53.16)		
median (min, max)	46.94 (36.43, 69.78)	47.78 (12.10, 69.78)		
missing	34 (51.5%)	23 (35.9%)		

	0<br>*n = 66*	1<br>*n = 64*	Linear regression<br>*estimate (95%CI)*	*p*
p__0__k52sel				
mean (95%CI)	44.49 (42.79;46.20)	44.41 (42.82;46.00)		
median (min, max)	44.63 (30.06, 69.78)	44.58 (31.24, 69.78)		
missing	6 (9.1%)	8 (12.5%)		
p__4__k52sel				
mean (95%CI)	44.98 (43.10;46.85)	45.82 (44.12;47.52)		
median (min, max)	46.18 (28.90, 60.11)	45.34 (34.05, 64.94)		
missing	15 (22.7%)	13 (20.3%)		
p__8__k52sel				
mean (95%CI)	46.44 (44.35;48.52)	47.16 (45.21;49.11)		
median (min, max)	47.07 (25.66, 69.78)	46.09 (32.22, 69.78)		
missing	16 (24.2%)	11 (17.2%)		
p__16__k52sel			0.28 (-1.93;2.49)	0.803
mean (95%CI)	48.19 (46.14;50.25)	48.49 (46.16;50.82)		
median (min, max)	47.78 (36.37, 60.11)	47.78 (37.85, 69.78)		
missing	24 (36.4%)	20 (31.2%)		

	0	1	Linear regression	
	n = 66	*n = 64*	*estimate (95%CI)*	*p*
d_0_k52aut				
mean (95%CI)	47.94 (45.49;50.39)	46.18 (43.70;48.65)		
median (min, max)	45.17 (31.57, 68.75)	46.01 (29.16, 68.75)		
missing	12 (18.2%)	12 (18.8%)		
d_4_k52aut				
mean (95%CI)	47.86 (45.22;50.50)	48.58 (45.89;51.27)		
median (min, max)	46.85 (29.16, 68.75)	46.85 (31.57, 68.75)		
missing	24 (36.4%)	16 (25%)		
d_8_k52aut				
mean (95%CI)	49.64 (46.41;52.87)	51.05 (48.03;54.07)		
median (min, max)	46.85 (37.35, 68.75)	48.70 (37.35, 68.75)		
missing	29 (43.9%)	20 (31.2%)		
d_16_k52aut			1.53 (-2.81;5.87)	0.483
mean (95%CI)	51.96 (48.13;55.79)	52.42 (49.13;55.70)		
median (min, max)	48.70 (37.35, 68.75)	48.70 (31.57, 68.75)		
missing	35 (53%)	22 (34.4%)		

	0	1	Linear regression	
	n = 66	*n = 64*	*estimate (95%CI)*	*p*
p_0_k52aut				
mean (95%CI)	47.43 (45.50;49.37)	47.32 (45.62;49.02)		
median (min, max)	46.41 (35.61, 68.75)	46.89 (31.57, 64.64)		
missing	6 (9.1%)	8 (12.5%)		
p_4_k52aut				
mean (95%CI)	49.34 (47.12;51.55)	46.99 (45.12;48.86)		
median (min, max)	48.41 (30.37, 68.75)	46.94 (33.70, 68.75)		
missing	15 (22.7%)	13 (20.3%)		
p_8_k52aut				
mean (95%CI)	49.71 (47.40;52.01)	49.55 (47.61;51.50)		
median (min, max)	47.78 (38.16, 68.75)	47.78 (34.46, 68.75)		
missing	17 (25.8%)	11 (17.2%)		
p_16_k52aut			0.20 (-2.82;3.21)	0.896
mean (95%CI)	51.68 (49.01;54.35)	51.65 (49.19;54.10)		
median (min, max)	48.70 (33.70, 68.75)	50.77 (38.08, 68.75)		
missing	24 (36.4%)	20 (31.2%)		

	0<br>*n = 66*	1<br>*n = 64*	Linear regression<br>*estimate (95%CI)*	*p*
d_0_k52par				
mean (95%CI)	48.77 (46.40;51.15)	47.99 (45.51;50.46)		
median (min, max)	47.50 (30.18, 65.87)	47.50 (27.06, 65.87)		
missing	13 (19.7%)	12 (18.8%)		
d_4_k52par				
mean (95%CI)	46.56 (44.00;49.13)	49.56 (47.14;51.98)		
median (min, max)	44.09 (34.32, 65.87)	49.50 (28.68, 65.87)		
missing	24 (36.4%)	16 (25%)		
d_8_k52par				
mean (95%CI)	47.12 (44.03;50.22)	51.14 (48.37;53.91)		
median (min, max)	45.72 (32.97, 65.87)	48.50 (27.06, 65.87)		
missing	29 (43.9%)	20 (31.2%)		
d_16_k52par			2.41 (-1.23;6.05)	0.191
mean (95%CI)	49.13 (45.83;52.43)	51.44 (48.38;54.49)		
median (min, max)	46.61 (34.32, 65.87)	49.50 (31.61, 65.87)		
missing	34 (51.5%)	22 (34.4%)		

	0<br>*n = 66*	1<br>*n = 64*	Linear regression<br>*estimate (95%CI)*	*p*
p_0_k52par				
mean (95%CI)	42.19 (40.74;43.64)	41.99 (40.31;43.67)		
median (min, max)	41.35 (31.61, 56.59)	42.29 (25.27, 65.87)		
missing	7 (10.6%)	8 (12.5%)		
p_4_k52par				
mean (95%CI)	42.65 (40.61;44.68)	42.91 (41.46;44.37)		
median (min, max)	41.52 (24.23, 65.87)	43.41 (28.62, 54.65)		
missing	16 (24.2%)	14 (21.9%)		
p_8_k52par				
mean (95%CI)	43.50 (41.30;45.69)	44.75 (43.00;46.51)		
median (min, max)	42.55 (15.27, 65.87)	45.02 (32.97, 62.20)		
missing	16 (24.2%)	11 (17.2%)		
p_16_k52par			1.32 (-0.47;3.12)	0.146
mean (95%CI)	45.12 (42.90;47.34)	45.36 (43.44;47.29)		
median (min, max)	44.09 (34.32, 60.26)	45.51 (34.32, 65.87)		
missing	24 (36.4%)	20 (31.2%)		

	0<br>*n = 66*	1<br>*n = 64*	Linear regression<br>*estimate (95%CI)*	*p*
d_0_k52fin				
mean (95%CI)	56.13 (53.98;58.29)	55.49 (53.21;57.77)		
median (min, max)	62.86 (37.47, 62.86)	56.35 (35.12, 62.86)		
missing	13 (19.7%)	13 (20.3%)		
d_4_k52fin				
mean (95%CI)	55.57 (52.73;58.41)	55.39 (52.90;57.89)		
median (min, max)	62.86 (35.12, 62.86)	59.61 (29.15, 62.86)		
missing	24 (36.4%)	16 (25%)		
d_8_k52fin				
mean (95%CI)	56.19 (52.97;59.40)	55.31 (52.49;58.13)		
median (min, max)	62.86 (23.24, 62.86)	62.86 (29.15, 62.86)		
missing	30 (45.5%)	20 (31.2%)		
d_16_k52fin			-1.53 (-5.41;2.34)	0.432
mean (95%CI)	56.29 (52.93;59.64)	56.55 (53.18;59.92)		
median (min, max)	62.86 (35.12, 62.86)	62.86 (23.24, 62.86)		
missing	34 (51.5%)	22 (34.4%)		

	0<br>*n = 66*	1<br>*n = 64*	Linear regression<br>*estimate (95%CI)*	*p*
p_0_k52fin				
mean (95%CI)	52.66 (50.32;55.00)	52.15 (49.97;54.34)		
median (min, max)	52.81 (23.24, 62.86)	52.40 (32.48, 62.86)		
missing	7 (10.6%)	8 (12.5%)		
p_4_k52fin				
mean (95%CI)	54.01 (51.94;56.09)	51.89 (49.59;54.18)		
median (min, max)	56.07 (37.42, 62.86)	50.84 (32.58, 62.86)		
missing	15 (22.7%)	13 (20.3%)		
p_8_k52fin				
mean (95%CI)	54.01 (51.75;56.26)	55.13 (53.05;57.20)		
median (min, max)	56.21 (38.59, 62.86)	56.07 (38.52, 62.86)		
missing	16 (24.2%)	11 (17.2%)		
p_16_k52fin			-1.09 (-3.96;1.78)	0.452
mean (95%CI)	55.05 (53.04;57.06)	53.43 (50.80;56.06)		
median (min, max)	56.07 (38.52, 62.86)	53.17 (29.18, 62.86)		
missing	24 (36.4%)	20 (31.2%)		

	**0**<br>*n = 66*	**1**<br>*n = 64*	**Linear regression**<br>*estimate (95%CI)*	*p*
d_0_k52soc				
mean (95%CI)	43.88 (41.25;46.50)	42.11 (38.79;45.44)		
median (min, max)	43.60 (9.40, 62.66)	42.20 (9.40, 71.46)		
missing	12 (18.2%)	14 (21.9%)		
d_4_k52soc				
mean (95%CI)	41.27 (38.07;44.46)	43.23 (40.54;45.91)		
median (min, max)	43.60 (9.40, 54.93)	43.60 (24.88, 71.46)		
missing	25 (37.9%)	16 (25%)		
d_8_k52soc				
mean (95%CI)	43.04 (39.48;46.59)	46.17 (42.45;49.90)		
median (min, max)	42.90 (9.40, 71.46)	46.66 (21.95, 71.46)		
missing	30 (45.5%)	21 (32.8%)		
d_16_k52soc			2.93 (-1.50;7.36)	0.190
mean (95%CI)	44.87 (41.61;48.14)	47.33 (43.40;51.25)		
median (min, max)	45.87 (24.88, 71.46)	45.08 (24.88, 71.46)		
missing	34 (51.5%)	23 (35.9%)		

	**0**<br>*n = 66*	**1**<br>*n = 64*	**Linear regression**<br>*estimate (95%CI)*	*p*
p_0_k52soc				
mean (95%CI)	37.81 (35.40;40.23)	39.09 (36.86;41.31)		
median (min, max)	39.49 (9.40, 60.85)	38.82 (15.68, 63.19)		
missing	8 (12.1%)	8 (12.5%)		
p_4_k52soc				
mean (95%CI)	37.94 (35.21;40.67)	39.31 (37.17;41.45)		
median (min, max)	38.82 (9.40, 58.14)	39.49 (18.31, 64.80)		
missing	15 (22.7%)	13 (20.3%)		
p_8_k52soc				
mean (95%CI)	39.31 (37.00;41.62)	41.56 (39.21;43.92)		
median (min, max)	39.16 (9.40, 58.80)	41.05 (9.40, 67.06)		
missing	16 (24.2%)	11 (17.2%)		
p_16_k52soc			-0.55 (-3.60;2.49)	0.717
mean (95%CI)	42.13 (39.37;44.88)	42.25 (39.34;45.15)		
median (min, max)	39.52 (23.10, 71.46)	42.93 (9.40, 60.85)		
missing	24 (36.4%)	20 (31.2%)		

	**0** *n = 66*	**1** *n = 64*	**Linear regression** *estimate (95%CI)*	*p*
d_0_k52sch				
mean (95%CI)	48.30 (45.53;51.07)	47.43 (45.07;49.80)		
median (min, max)	48.61 (14.02, 73.80)	46.94 (25.68, 73.80)		
missing	13 (19.7%)	13 (20.3%)		
d_4_k52sch				
mean (95%CI)	45.53 (41.66;49.40)	49.13 (46.00;52.25)		
median (min, max)	47.77 (14.02, 73.80)	46.94 (14.02, 73.80)		
missing	24 (36.4%)	16 (25%)		
d_8_k52sch				
mean (95%CI)	47.37 (43.29;51.44)	51.05 (46.95;55.15)		
median (min, max)	48.61 (14.02, 73.80)	48.61 (14.02, 73.80)		
missing	30 (45.5%)	21 (32.8%)		
d_16_k52sch			3.08 (-1.57;7.74)	0.190
mean (95%CI)	50.30 (46.18;54.43)	53.62 (49.81;57.44)		
median (min, max)	52.23 (14.02, 73.80)	52.23 (25.68, 73.80)		
missing	36 (54.5%)	23 (35.9%)		

	**0** *n = 66*	**1** *n = 64*	**Linear regression** *estimate (95%CI)*	*p*
p_0_k52sch				
mean (95%CI)	46.99 (44.73;49.24)	45.45 (43.70;47.19)		
median (min, max)	46.67 (22.24, 73.80)	45.41 (33.12, 61.87)		
missing	6 (9.1%)	8 (12.5%)		
p_4_k52sch				
mean (95%CI)	45.15 (42.59;47.70)	44.38 (41.86;46.89)		
median (min, max)	46.14 (14.02, 63.91)	45.38 (14.02, 58.88)		
missing	15 (22.7%)	13 (20.3%)		
p_8_k52sch				
mean (95%CI)	45.85 (43.14;48.55)	47.97 (45.51;50.43)		
median (min, max)	48.61 (14.02, 67.83)	48.61 (14.02, 65.94)		
missing	17 (25.8%)	12 (18.8%)		
p_16_k52sch			2.17 (-1.21;5.54)	0.205
mean (95%CI)	48.79 (45.72;51.85)	49.49 (47.13;51.86)		
median (min, max)	50.52 (14.02, 64.01)	49.54 (30.30, 73.80)		
missing	24 (36.4%)	20 (31.2%)		

	0	1	Linear regression	
	n = 66	*n = 64*	*estimate (95%CI)*	*p*
d_0_k52bul				
mean (95%CI)	50.89 (48.42;53.37)	50.93 (48.18;53.68)		
median (min, max)	58.85 (29.13, 58.85)	58.85 (24.99, 58.85)		
missing	12 (18.2%)	11 (17.2%)		
d_4_k52bul				
mean (95%CI)	51.46 (48.51;54.42)	50.93 (48.09;53.76)		
median (min, max)	58.85 (31.08, 58.85)	58.85 (24.99, 58.85)		
missing	24 (36.4%)	15 (23.4%)		
d_8_k52bul				
mean (95%CI)	52.18 (49.41;54.96)	53.40 (50.62;56.18)		
median (min, max)	58.85 (35.44, 58.85)	58.85 (22.38, 58.85)		
missing	29 (43.9%)	20 (31.2%)		
d_16_k52bul			-0.08 (-4.80;4.64)	0.972
mean (95%CI)	52.81 (49.45;56.18)	52.87 (49.59;56.14)		
median (min, max)	58.85 (33.13, 58.85)	58.85 (10.99, 58.85)		
missing	34 (51.5%)	22 (34.4%)		

	0	1	Linear regression	
	n = 66	*n = 64*	*estimate (95%CI)*	*p*
p_0_k52bul				
mean (95%CI)	48.26 (45.73;50.79)	46.78 (44.20;49.35)		
median (min, max)	48.57 (28.03, 58.85)	48.07 (28.03, 58.85)		
missing	7 (10.6%)	8 (12.5%)		
p_4_k52bul				
mean (95%CI)	50.39 (47.78;53.01)	48.37 (45.90;50.84)		
median (min, max)	53.46 (31.64, 58.85)	48.57 (24.99, 58.85)		
missing	15 (22.7%)	13 (20.3%)		
p_8_k52bul				
mean (95%CI)	50.76 (48.18;53.34)	52.29 (49.97;54.62)		
median (min, max)	53.46 (26.07, 58.85)	58.85 (29.13, 58.85)		
missing	17 (25.8%)	11 (17.2%)		
p_16_k52bul			1.53 (-1.46;4.52)	0.311
mean (95%CI)	50.86 (48.29;53.44)	52.78 (50.53;55.03)		
median (min, max)	53.46 (32.11, 58.85)	58.85 (33.71, 58.85)		
missing	24 (36.4%)	20 (31.2%)		

29 October 2022

TECTO trial

Markus Harboe Olsen & Janus Christian Jakobsen

Markus Harboe Olsen (MHO) finished the analysis at 16:17, 28 October 2022 and Janus Christian Jakobsen (JCJ) finished the analysis at 16:14, 28 October 2021.

The results from the analyses carried out in R (by MHO) and in Stata 17 (by JCJ) are not identical. Analyses carried out for each outcome and question are different. However, the overall conclusions and all primary analyses are identical with subsequent identical results.

Complete case analysis is suggested as the primary analysis of the primary outcome. Multiple imputation was not used because data were only missing on one outcome and relevant ancillary variables were not available.

Due to the distribution of data, NEQ was analysed using non-parametric analysis methods. MHO used Wilcoxon rank sum test without any covariates and JCJ used Van Elteren's test with CYBOCS strata as a covariate. Both analyses showed no difference between the groups, and even though Van Elteren's test might be more precise the preplanned Wilcoxon rank sum test for count outcomes will be used as the primary analysis.

When assessing the potential interaction between the treatment variable and motivation (moderator), skewed distribution of the 'motivation' variable lead to analytic problems. MHO transformed the continuous variable to a dichotomous variable (dichotomised at the mean). JCJ transformed the variable to a factor variable with 7 levels. Both analyses showed no significant interaction. The dichotomised variable is included in the primary analysis.

One participant was allocated in one group but was treated as in the opposite group. The first analyses were carried out with one participant in the wrong group. This participant is now handled as being in the group with the carried out intervention.

Appendices:

1. The report in R – 16:17, 28 October 2022
2. The report in Stata – 16:14, 28 October 2021

Statistical report for the TECTO-trial

Markus Harboe Olsen

28 oktober 2022 16:17

Table of contents

Primary outcome 3
CYBOCS 16 - Complete case analysis 3
Assumptions for linear regression 3
Analysis 4
Sensitivity - per protocol 5

CYBOCS 16 - Multiple imputation 5
Multiple imputations 5
Assumptions for linear regression 7
Analysis 8
Sensitivity - per protocol 9

Secondary outcomes 10
KIDSCREEN-10 10
Assumptions for linear regression 10
Analysis 11
NEQ 12
Assumptions for linear regression 12
Analysis 12

Moderators 13
COVID-19 as a moderator 13
Interaction analysis 13
Confidence in treatment as moderator 13
Interaction analysis 13
Motivation for treatment as moderator 14
Interaction analysis 14

Therapeutic alliance as moderator 15
Interaction analysis 15
Compliance as moderator 15
Interaction analysis 15

Primary outcome

CYBOCS 16 - Complete case analysis

Assumptions for linear regression

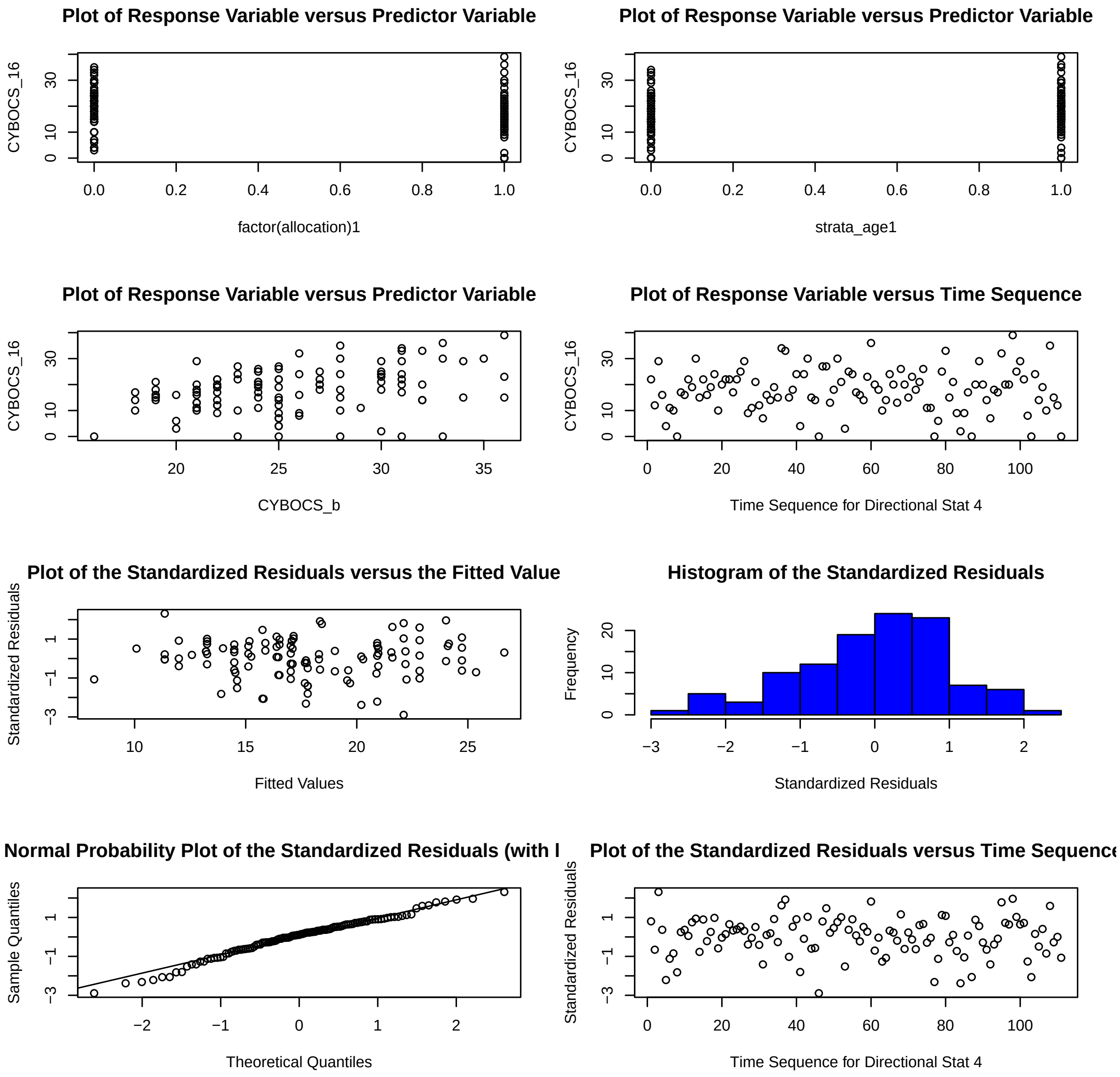

	Value	p-value	Decision
Global Stat	5.4674288	0.2426074	Assumptions acceptable.
Skewness	3.4145641	0.0646235	Assumptions acceptable.
Kurtosis	0.1818756	0.6697663	Assumptions acceptable.
Link Function	1.7309546	0.1882890	Assumptions acceptable.
Heteroscedasticity	0.1400345	0.7082468	Assumptions acceptable.

Analysis

	CYBOCS_16		
Predictors	Estimates	CI	p
(Intercept)	1.91	-6.62 – 10.43	0.658
allocation [1]	-3.89	-6.83 – -0.96	**0.010**
strata age [1]	3.13	0.19 – 6.07	**0.037**
CYBOCS b	0.64	0.32 – 0.95	**<0.001**
Observations	111		
R^2 / R^2 adjusted	0.204 / 0.182		

Sensitivity - per protocol

	CYBOCS_16		
Predictors	Estimates	CI	p
(Intercept)	-0.79	-11.48 – 9.90	0.884
allocation	-3.96	-6.90 – -1.01	**0.009**
strata age [1]	3.25	0.29 – 6.21	**0.032**
strata cybocs [1]	-1.99	-6.73 – 2.76	0.409
CYBOCS b	0.79	0.30 – 1.28	**0.002**
Observations	111		
R^2 / R^2 adjusted	0.209 / 0.179		

CYBOCS 16 - Multiple imputation

Multiple imputations

```
Complete data summary


   Min. 1st Qu.  Median    Mean 3rd Qu.    Max.    NA's
    0.0    12.5    18.0    17.8    23.5    39.0      19

Imputed data summary


   Min. 1st Qu.  Median    Mean 3rd Qu.    Max.
   0.00   13.25   18.50   18.38   24.00   39.00
```

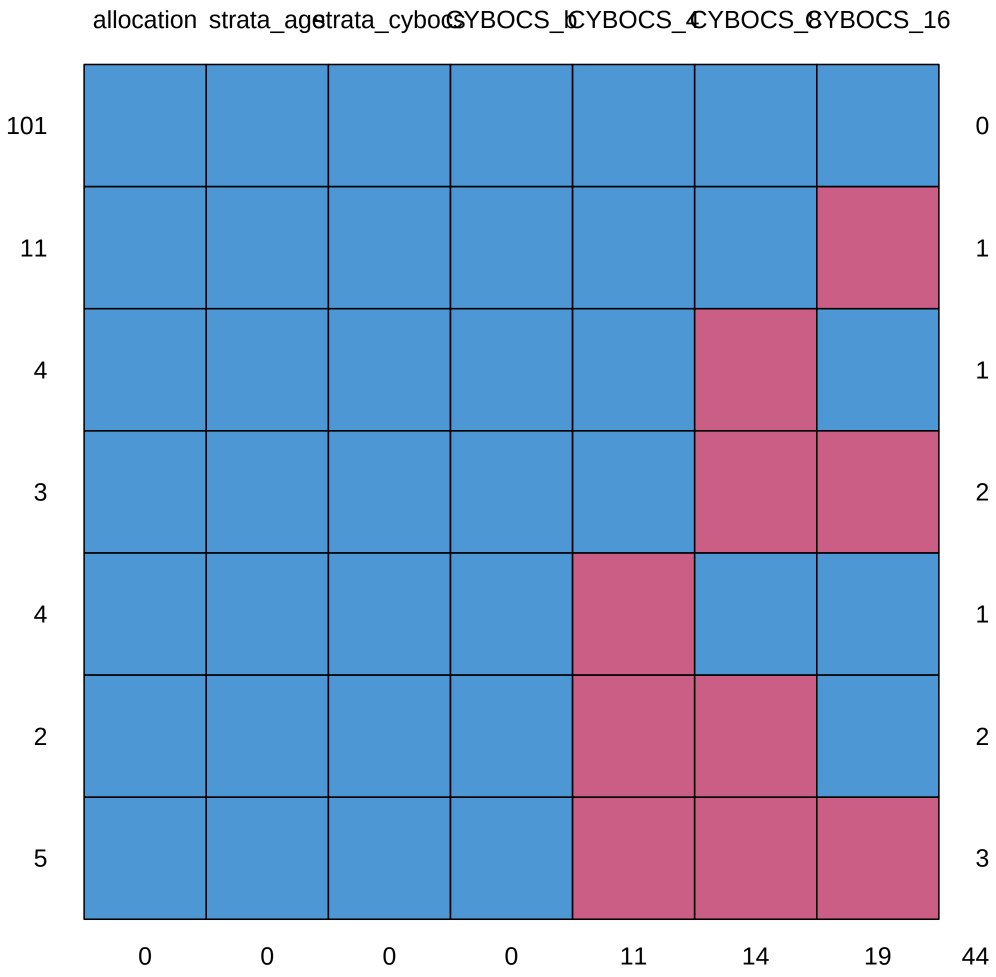
allocation
strata_age
strata_cybocs
CYBOCS_b
CYBOCS_4
CYBOCS_8
CYBOCS_16
101
11
4
3
4
2
5
0
1
1
2
1
2
3
0
0
0
0
11
14
19
44

Assumptions for linear regression

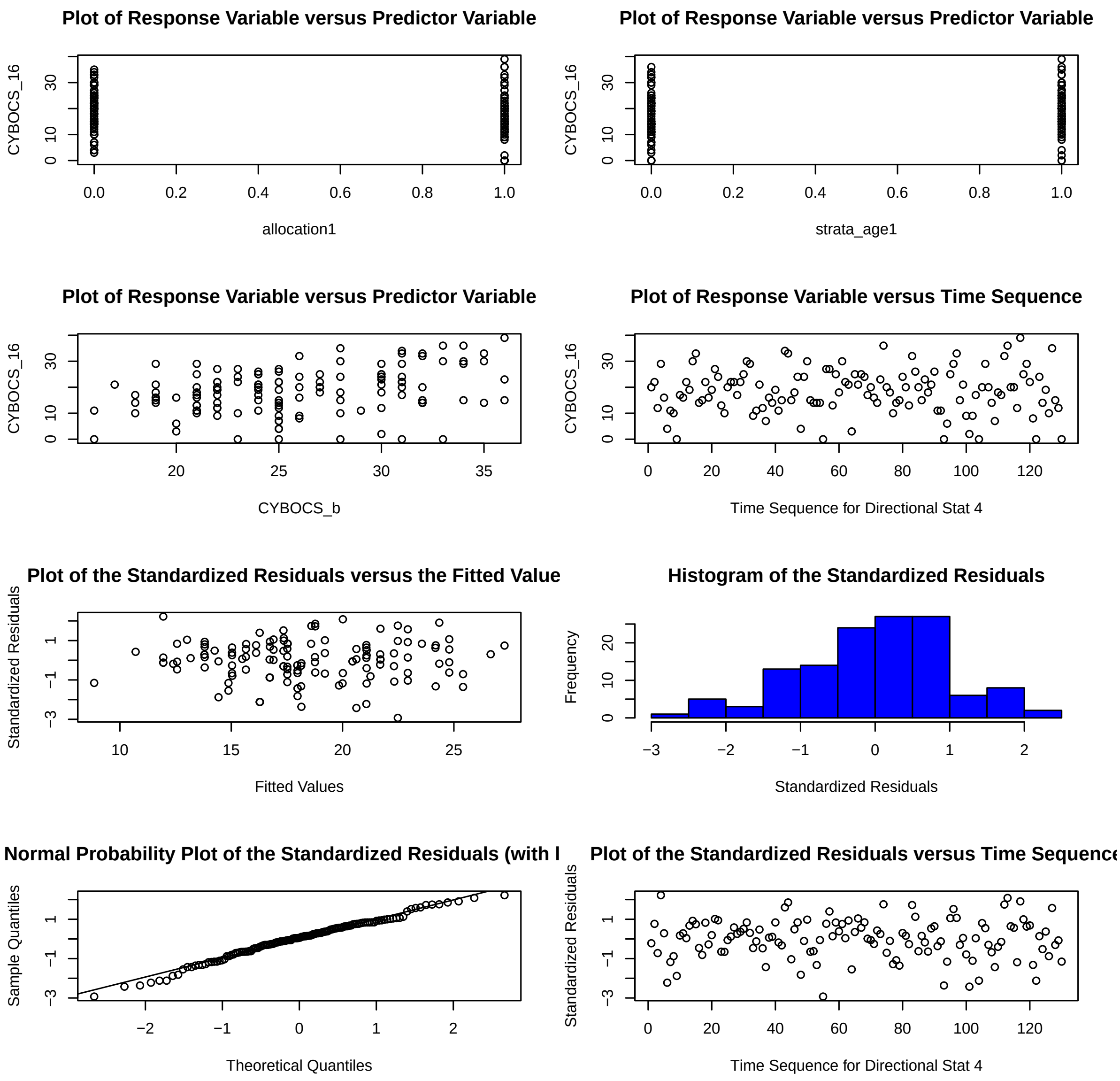

	Value	p-value	Decision
Global Stat	4.8015608	0.3082712	Assumptions acceptable.
Skewness	2.8682257	0.0903444	Assumptions acceptable.
Kurtosis	0.0676469	0.7947941	Assumptions acceptable.
Link Function	0.5460465	0.4599376	Assumptions acceptable.

	Value	p-value	Decision
Heteroscedasticity	1.3196416	0.2506564	Assumptions acceptable.

Analysis

	CYBOCS_16		
Predictors	Estimates	CI	p
(Intercept)	2.47	-5.11 – 10.05	0.521
allocation [1]	-3.55	-6.26 – -0.84	**0.011**
strata age [1]	3.09	0.37 – 5.81	**0.026**
CYBOCS b	0.62	0.34 – 0.90	**<0.001**
Observations	130		
R^2 / R^2 adjusted	0.195 / 0.176		

Sensitivity - per protocol

	CYBOCS_16		
Predictors	Estimates	CI	p
(Intercept)	-1.37	-10.76 – 8.01	0.773
allocation [1]	-3.58	-6.28 – -0.88	**0.010**
strata age [1]	3.25	0.53 – 5.97	**0.020**
strata cybocs [1]	-3.04	-7.45 – 1.37	0.175
CYBOCS b	0.84	0.42 – 1.27	**<0.001**
Observations	130		
R^2 / R^2 adjusted	0.207 / 0.182		

Secondary outcomes

KIDSCREEN-10

Assumptions for linear regression

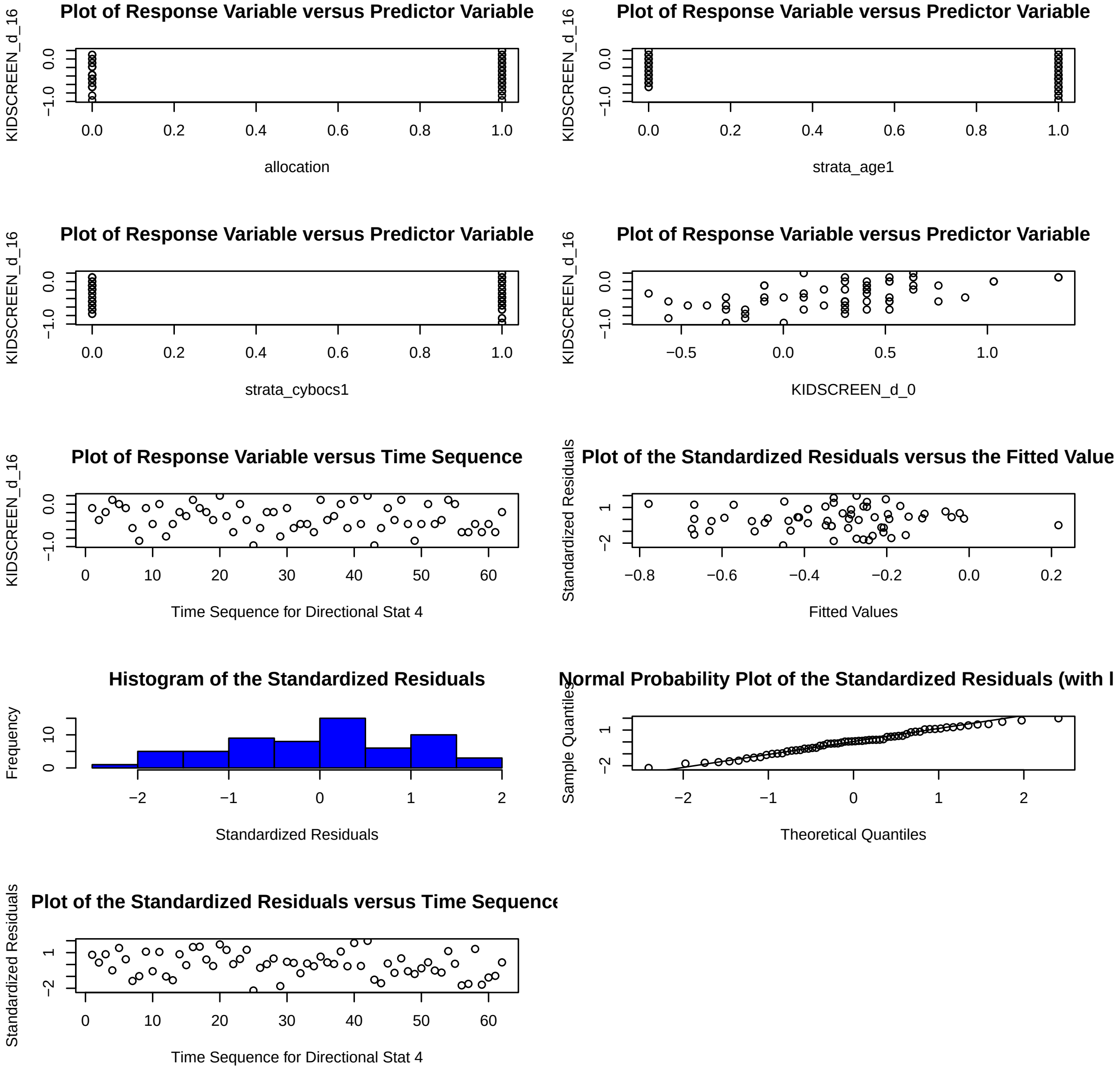

	Value	p-value	Decision
Global Stat	1.4638081	0.8330325	Assumptions acceptable.
Skewness	0.1261296	0.7224793	Assumptions acceptable.
Kurtosis	1.2173536	0.2698806	Assumptions acceptable.
Link Function	0.0099552	0.9205224	Assumptions acceptable.
Heteroscedasticity	0.1103698	0.7397235	Assumptions acceptable.

Analysis

	KIDSCREEN_d_16		
Predictors	Estimates	CI	p
(Intercept)	-0.41	-0.57 – -0.26	**<0.001**
allocation	0.10	-0.02 – 0.23	0.106
strata age [1]	-0.14	-0.27 – -0.01	**0.033**
strata cybocs [1]	-0.01	-0.13 – 0.12	0.938
KIDSCREEN d 0	0.39	0.24 – 0.54	**<0.001**
Observations	62		
R^2 / R^2 adjusted	0.386 / 0.343		

NEQ

Assumptions for linear regression

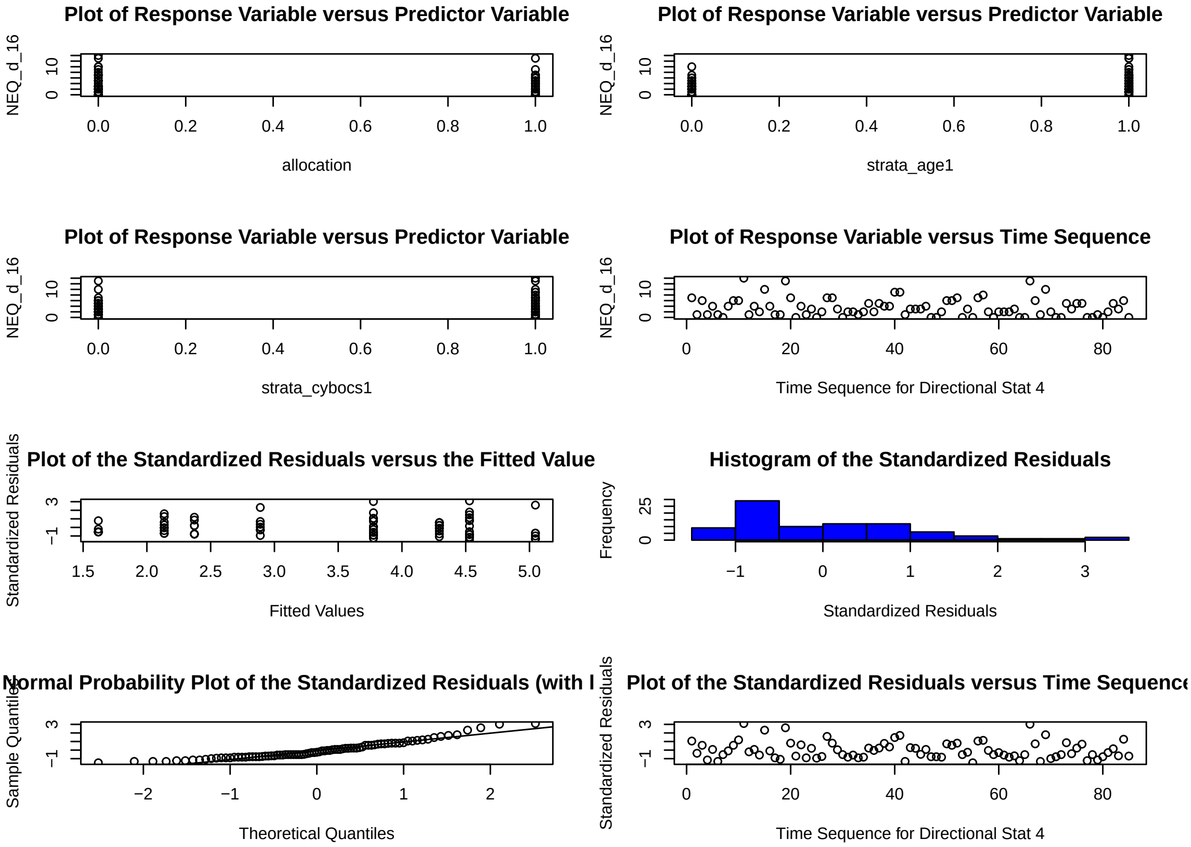

	Value	p-value	Decision
Global Stat	20.733748	0.0003576	Assumptions NOT satisfied!
Skewness	14.952486	0.0001103	Assumptions NOT satisfied!
Kurtosis	2.422047	0.1196385	Assumptions acceptable.
Link Function	2.674305	0.1019799	Assumptions acceptable.
Heteroscedasticity	0.684910	0.4079011	Assumptions acceptable.

Analysis

```
Wilcoxon rank sum test with continuity correction
```

```
data:  NEQ_d_16 by allocation
W = 968.5, p-value = 0.5037
alternative hypothesis: true location shift is not equal to 0
95 percent confidence interval:
 -0.9999777  1.9999693
sample estimates:
difference in location
          8.887042e-05
```

Moderators

COVID-19 as a moderator

Interaction analysis

		CYBOCS_16	
Predictors	Estimates	CI	p
(Intercept)	1.13	-7.73 – 9.98	0.801
allocation	-4.33	-7.97 – -0.69	**0.020**
strata age [1]	3.08	0.12 – 6.05	**0.042**
CYBOCS b	0.66	0.34 – 0.98	**<0.001**
before corona	0.59	-4.08 – 5.26	0.804
allocation * before corona	1.08	-5.21 – 7.38	0.734
Observations	111		
R^2 / R^2 adjusted	0.209 / 0.171		

Confidence in treatment as moderator

Interaction analysis

		CYBOCS_16	
Predictors	Estimates	CI	p
(Intercept)	9.02	-2.89 – 20.93	0.136
allocation	-2.97	-14.27 – 8.32	0.601
strata age [1]	4.20	0.74 – 7.66	**0.018**
CYBOCS b	0.48	0.10 – 0.85	**0.013**

	CYBOCS_16		
moderator confidence [4]	-4.74	-12.31 – 2.84	0.217
moderator confidence [5]	-3.59	-11.00 – 3.82	0.338
moderator confidence [6]	-6.77	-15.45 – 1.92	0.125
moderator confidence [7]	-9.37	-20.18 – 1.44	0.088
allocation * moderator confidence [4]	8.37	-5.89 – 22.62	0.246
allocation * moderator confidence [5]	-0.06	-12.58 – 12.47	0.993
allocation * moderator confidence [6]	-0.41	-13.70 – 12.87	0.951
Observations	90		
R^2 / R^2 adjusted	0.297 / 0.208		

Motivation for treatment as moderator

Interaction analysis

Dichotomise at < 5.393 is 0 and >= 5.393 is 1::: {.cell-output-display}

	CYBOCS_16		
Predictors	Estimates	CI	p
(Intercept)	0.37	-8.34 – 9.07	0.934
allocation	-0.38	-4.76 – 4.00	0.865
moderator motivation dicho [1]	0.91	-3.26 – 5.08	0.666
strata age [1]	3.00	0.07 – 5.92	**0.045**
CYBOCS b	0.67	0.36 – 0.98	**<0.001**
allocation * moderator motivation dicho [1]	-5.57	-11.44 – 0.31	0.063
Observations	104		

	CYBOCS_16
R^2 / R^2 adjusted	0.268 / 0.230

:::

Therapeutic alliance as moderator

Interaction analysis

	CYBOCS_16		
Predictors	Estimates	CI	p
(Intercept)	-5.21	-30.93 – 20.51	0.688
allocation	7.87	-23.37 – 39.12	0.618
strata age [1]	3.31	0.18 – 6.44	**0.038**
CYBOCS b	0.69	0.37 – 1.01	**<0.001**
moderator alliance	0.18	-0.64 – 1.00	0.662
allocation * moderator alliance	-0.38	-1.40 – 0.64	0.461
Observations	99		
R^2 / R^2 adjusted	0.241 / 0.201		

Compliance as moderator

Interaction analysis

	CYBOCS_16		
Predictors	Estimates	CI	p
(Intercept)	16.91	2.04 – 31.79	**0.026**
allocation	-2.35	-17.42 – 12.72	0.758
strata age [1]	2.90	0.12 – 5.68	**0.041**
CYBOCS b	0.59	0.30 – 0.89	**<0.001**
moderator compliance	-2.93	-5.50 – -0.36	**0.026**
allocation * moderator compliance	-0.40	-3.58 – 2.78	0.801

	CYBOCS_16
Observations	110
R^2 / R^2 adjusted	0.318 / 0.285

```
--------------------------------------------------------------------------------------------------
      name:  <unnamed>
       log:  /Users/janusjakobsen/Documents/OUTPUT_2.log
  log type:  text
 opened on:  28 Oct 2022, 16:14:46

. *SYNTAX

. 
. *TECTO

. 
. *CYBOCS

. 
. *describe

. 
. codebook CYBOCS_16 if allocation ==0

--------------------------------------------------------------------------------------------------
CYBOCS_16                                                                                CYBOCS_16
--------------------------------------------------------------------------------------------------

                  Type: Numeric (float)

                 Range: [3,35]                        Units: 1
         Unique values: 25                        Missing .: 14/66

                  Mean: 19.9038
             Std. dev.: 8.09081

           Percentiles:     10%       25%       50%       75%       90%
                              7        15      20.5        25        30

. codebook CYBOCS_16 if allocation ==1

--------------------------------------------------------------------------------------------------
```

```
CYBOCS_16                                                                                  CYBOCS_16
----------------------------------------------------------------------------------------------------

                  Type: Numeric (float)

                 Range: [0,39]                           Units: 1
         Unique values: 26                           Missing .: 5/64

                  Mean: 15.9492
             Std. dev.: 8.68297

           Percentiles:     10%       25%       50%       75%       90%
                              0        11        16        21        27

.
. ttest CYBOCS_16, by(allocation)

Two-sample t test with equal variances
------------------------------------------------------------------------------
   Group |     Obs        Mean    Std. err.   Std. dev.   [95% conf. interval]
---------+--------------------------------------------------------------------
       0 |      52    19.90385    1.121993    8.090807    17.65135    22.15634
       1 |      59    15.94915    1.130426    8.682967    13.68636    18.21195
---------+--------------------------------------------------------------------
Combined |     111     17.8018    .8166791    8.604248    16.18334    19.42027
---------+--------------------------------------------------------------------
    diff |            3.954694    1.599876                 .783792    7.125595
------------------------------------------------------------------------------
    diff = mean(0) - mean(1)                                      t =   2.4719
H0: diff = 0                                     Degrees of freedom =      109

    Ha: diff < 0                 Ha: diff != 0                 Ha: diff > 0
 Pr(T < t) = 0.9925         Pr(|T| > |t|) = 0.0150          Pr(T > t) = 0.0075

.
. *Linear regression CYBOCS

.
```

```
. regress CYBOCS_16 i.allocation i.strata_age CYBOCS_b

      Source |       SS           df       MS      Number of obs   =       111
-------------+----------------------------------   F(3, 107)       =      9.15
       Model |  1662.03838         3  554.012793   Prob > F        =    0.0000
    Residual |  6481.60126       107  60.5757127   R-squared       =    0.2041
-------------+----------------------------------   Adj R-squared   =    0.1818
       Total |  8143.63964       110  74.0330876   Root MSE        =     7.783

------------------------------------------------------------------------------
   CYBOCS_16 | Coefficient  Std. err.      t    P>|t|     [95% conf. interval]
-------------+----------------------------------------------------------------
1.allocation |  -3.893854   1.481067    -2.63   0.010    -6.829896   -.9578122
1.strata_age |   3.129873   1.485171     2.11   0.037     .1856938    6.074052
    CYBOCS_b |   .6354153   .1587409     4.00   0.000       .32073    .9501005
       _cons |   1.905465   4.298449     0.44   0.658    -6.615708    10.42664
------------------------------------------------------------------------------

.
.
. *Interaction corona

.
. regress CYBOCS_16 i.allocation##before_corona i.strata_age CYBOCS_b

      Source |       SS           df       MS      Number of obs   =       111
-------------+----------------------------------   F(5, 105)       =      5.55
       Model |  1702.83388         5  340.566776   Prob > F        =    0.0001
    Residual |  6440.80576       105  61.3410072   R-squared       =    0.2091
-------------+----------------------------------   Adj R-squared   =    0.1714
       Total |  8143.63964       110  74.0330876   Root MSE        =    7.8321

------------------------------------------------------------------------------------------
               CYBOCS_16 | Coefficient  Std. err.      t    P>|t|     [95% conf. interval]
-------------------------+----------------------------------------------------------------
            1.allocation |  -4.330167   1.835981    -2.36   0.020    -7.970579   -.6897553
         1.before_corona |   .5867836   2.354848     0.25   0.804    -4.082444    5.256012
                         |
```

```
allocation#before_corona |
                    1 1  |   1.083186   3.176371     0.34   0.734    -5.214972    7.381343
                         |
            1.strata_age |   3.081625   1.495775     2.06   0.042     .1157801     6.04747
                CYBOCS_b |   .6597575    .162568     4.06   0.000     .3374152    .9820998
                   _cons |   1.125956    4.46584     0.25   0.801     -7.72898    9.980892
------------------------------------------------------------------------------------------

.
. mean CYBOCS_16, over(allocation before_corona)

Mean estimation                                                   Number of obs = 111

--------------------------------------------------------------------------------------
                                      |       Mean   Std. err.     [95% conf. interval]
--------------------------------------+-----------------------------------------------
c.CYBOCS_16@allocation#before_corona |
                                  0 0  |   19.77778    1.36167      17.07927    22.47629
                                  0 1  |    20.1875   2.041688      16.14135    24.23365
                                  1 0  |   15.86486   1.593984      12.70596    19.02377
                                  1 1  |   16.09091   1.462236       13.1931    18.98872
--------------------------------------------------------------------------------------

.
.
. *Moderator Confidence

. regress CYBOCS_16 i.allocation##i.moderator_confidence i.strata_age CYBOCS_b
note: 0b.allocation#7.moderator_confidence identifies no observations in the sample.
note: 1.allocation#7.moderator_confidence omitted because of collinearity.

      Source |       SS           df       MS      Number of obs   =        90
-------------+----------------------------------   F(10, 79)       =      3.34
       Model |  2042.52787        10  204.252787   Prob > F        =    0.0011
    Residual |  4827.26102        79  61.1045699   R-squared       =    0.2973
-------------+----------------------------------   Adj R-squared   =    0.2084
       Total |  6869.78889        89  77.1886392   Root MSE        =    7.8169
```

```
------------------------------------------------------------------------------------------------
                        CYBOCS_16 | Coefficient  Std. err.      t    P>|t|     [95% conf. interval]
----------------------------------+-------------------------------------------------------------
                     1.allocation |  -2.974873   5.673211    -0.52   0.601    -14.26712    8.317371
                                  |
            moderator_confidence |
                               4  |  -4.737013   3.806644    -1.24   0.217    -12.31395    2.839923
                               5  |  -3.589082   3.724702    -0.96   0.338    -11.00292    3.824752
                               6  |   -6.76566   4.362104    -1.55   0.125    -15.44821    1.916892
                               7  |  -9.371947   5.431424    -1.73   0.088    -20.18293    1.439032
                                  |
allocation#moderator_confidence |
                             0 7  |          0  (empty)
                             1 4  |   8.366484   7.160695     1.17   0.246    -5.886523    22.61949
                             1 5  |  -.0564698   6.292405    -0.01   0.993    -12.58119    12.46825
                             1 6  |  -.4121116   6.673463    -0.06   0.951    -13.69531    12.87108
                             1 7  |          0  (omitted)
                                  |
                     1.strata_age |   4.202489   1.738669     2.42   0.018     .7417548    7.663223
                         CYBOCS_b |   .4756566   .1868109     2.55   0.013     .1038188    .8474944
                            _cons |   9.022279   5.984749     1.51   0.136    -2.890066    20.93462
------------------------------------------------------------------------------------------------

.
. testparm i.allocation#i.moderator_confidence

 ( 1)  1.allocation#4.moderator_confidence = 0
 ( 2)  1.allocation#5.moderator_confidence = 0
 ( 3)  1.allocation#6.moderator_confidence = 0

       F(  3,    79) =    1.02
            Prob > F =    0.3869

.
. mean CYBOCS_16, over(allocation moderator_confidence)

Mean estimation                                                        Number of obs = 90
```

```
-----------------------------------------------------------------------------------------------
                                        |       Mean   Std. err.     [95% conf. interval]
----------------------------------------+------------------------------------------------------
c.CYBOCS_16@allocation#moderator_confidence |
                                    0 3  |   24.66667   3.353274      18.00378    31.32955
                                    0 4  |   18.33333   2.087757        14.185    22.48166
                                    0 5  |   19.94118    1.53984      16.88155    23.00081
                                    0 6  |   17.28571   4.596657      8.152256    26.41917
                                    0 7  |          0  (no observations)
                                    1 3  |         20   2.645751      14.74295    25.25705
                                    1 4  |       22.5   2.254625      18.02011    26.97989
                                    1 5  |   16.66667   2.039296      12.61463    20.71871
                                    1 6  |   13.61538   2.914461      7.824413    19.40636
                                    1 7  |       10.7   2.530042      5.672861    15.72714
-----------------------------------------------------------------------------------------------

.
.
. *Moderator_motivation

.
. *does not fit

. regress CYBOCS_16 i.allocation##c.moderator_motivation i.strata_age CYBOCS_b

      Source |       SS           df       MS      Number of obs   =       104
-------------+----------------------------------   F(5, 98)        =      9.48
       Model |  2421.27347         5  484.254694   Prob > F        =    0.0000
    Residual |  5006.63999        98  51.0881632   R-squared       =    0.3260
-------------+----------------------------------   Adj R-squared   =    0.2916
       Total |  7427.91346       103  72.1156647   Root MSE        =    7.1476

----------------------------------------------------------------------------------------------------
                        CYBOCS_16 | Coefficient  Std. err.      t    P>|t|     [95% conf. interval]
----------------------------------+-----------------------------------------------------------------
                     1.allocation |   10.55194   5.859288     1.80   0.075    -1.075622    22.17951
             moderator_motivation |   .0075989   .7877411     0.01   0.992    -1.555648    1.570845
                                  |
```

```
allocation#c.moderator_motivation |
                               1  |  -2.628248   1.052082    -2.50   0.014     -4.71607   -.5404255
                                  |
                    1.strata_age |   3.088247   1.423579     2.17   0.032     .2632001    5.913294
                        CYBOCS_b |   .6709268   .1509068     4.45   0.000     .3714571    .9703965
                           _cons |   .7373932   6.145842     0.12   0.905    -11.45883    12.93362
--------------------------------------------------------------------------------------------------

.
. *WALD

.
. regress CYBOCS_16 i.allocation c.moderator_motivation i.strata_age CYBOCS_b

      Source |       SS           df       MS      Number of obs   =       104
-------------+----------------------------------   F(4, 99)        =      9.77
       Model |  2102.44743         4  525.611857   Prob > F        =    0.0000
    Residual |  5325.46603        99  53.7925862   R-squared       =    0.2830
-------------+----------------------------------   Adj R-squared   =    0.2541
       Total |  7427.91346       103  72.1156647   Root MSE        =    7.3343

--------------------------------------------------------------------------------------
           CYBOCS_16 | Coefficient  Std. err.      t    P>|t|     [95% conf. interval]
---------------------+----------------------------------------------------------------
        1.allocation |  -3.659255   1.440166    -2.54   0.013    -6.516857   -.8016533
moderator_motivation |  -1.466755   .5353816    -2.74   0.007    -2.529068   -.4044414
        1.strata_age |   3.578967   1.446799     2.47   0.015     .7082025    6.449731
            CYBOCS_b |   .6494499   .1545981     4.20   0.000     .3426939     .956206
               _cons |   9.051179   5.301796     1.71   0.091    -1.468734    19.57109
--------------------------------------------------------------------------------------

.
. est store m1

.
. regress CYBOCS_16 i.allocation## c.moderator_motivation i.strata_age CYBOCS_b

      Source |       SS           df       MS      Number of obs   =       104
```

```
-------------+----------------------------------   F(5, 98)        =      9.48
       Model |  2421.27347         5  484.254694   Prob > F        =    0.0000
    Residual |  5006.63999        98  51.0881632   R-squared       =    0.3260
-------------+----------------------------------   Adj R-squared   =    0.2916
       Total |  7427.91346       103  72.1156647   Root MSE        =    7.1476

----------------------------------------------------------------------------------------------------
                       CYBOCS_16 | Coefficient  Std. err.      t    P>|t|     [95% conf. interval]
---------------------------------+------------------------------------------------------------------
                    1.allocation |   10.55194   5.859288     1.80   0.075    -1.075622    22.17951
           moderator_motivation |   .0075989   .7877411     0.01   0.992    -1.555648    1.570845
                                 |
allocation#c.moderator_motivation |
                              1  |  -2.628248   1.052082    -2.50   0.014     -4.71607   -.5404255
                                 |
                    1.strata_age |   3.088247   1.423579     2.17   0.032     .2632001    5.913294
                        CYBOCS_b |   .6709268   .1509068     4.45   0.000     .3714571    .9703965
                           _cons |   .7373932   6.145842     0.12   0.905    -11.45883    12.93362
----------------------------------------------------------------------------------------------------

.
. est store m2

.
. lrtest m2 m1

Likelihood-ratio test
Assumption: m1 nested within m2

 LR chi2(1) =   6.42
Prob > chi2 = 0.0113

.
.
. regress CYBOCS_16 i.allocation##i.Moderator_Moti_CATAG i.strata_age CYBOCS_b
note: 0b.allocation#1b.Moderator_Moti_CATAG identifies no observations in the sample.
note: 1.allocation#7.Moderator_Moti_CATAG omitted because of collinearity.
```

```
      Source |       SS           df       MS      Number of obs   =       104
-------------+----------------------------------   F(14, 89)       =      3.68
       Model |  2721.07548        14  194.362534   Prob > F        =    0.0001
    Residual |  4706.83798        89    52.88582   R-squared       =    0.3663
-------------+----------------------------------   Adj R-squared   =    0.2667
       Total |  7427.91346       103  72.1156647   Root MSE        =    7.2723

------------------------------------------------------------------------------------------------
                     CYBOCS_16 | Coefficient  Std. err.      t    P>|t|     [95% conf. interval]
-------------------------------+----------------------------------------------------------------
                  1.allocation |  -14.16688   4.118887    -3.44   0.001    -22.35102   -5.982735
                               |
          Moderator_Moti_CATAG |
                            2  |  -28.83799    9.38714    -3.07   0.003    -47.49004   -10.18595
                            3  |  -25.91395   9.356708    -2.77   0.007    -44.50553   -7.322372
                            4  |  -29.76646   8.851445    -3.36   0.001     -47.3541   -12.17883
                            5  |  -28.53061    8.57703    -3.33   0.001    -45.57299   -11.48823
                            6  |   -29.2877   8.626685    -3.40   0.001    -46.42874   -12.14666
                            7  |  -27.55898   8.073516    -3.41   0.001    -43.60089   -11.51708
                               |
allocation#Moderator_Moti_CATAG |
                          0 1  |          0  (empty)
                          1 2  |   14.24384   7.661259     1.86   0.066    -.9789185     29.4666
                          1 3  |   13.39985   6.518228     2.06   0.043      .448274    26.35144
                          1 4  |   12.04981   5.770071     2.09   0.040     .5848047    23.51482
                          1 5  |   11.23962    5.04977     2.23   0.029     1.205838    21.27341
                          1 6  |   10.69108   4.937005     2.17   0.033     .8813588    20.50081
                          1 7  |          0  (omitted)
                               |
                  1.strata_age |   2.705728   1.579969     1.71   0.090    -.4336361    5.845092
                      CYBOCS_b |   .7655051   .1679246     4.56   0.000     .4318424    1.099168
                         _cons |   27.09127   8.895225     3.05   0.003     9.416648    44.76589
------------------------------------------------------------------------------------------------

.
. testparm i.allocation#i.Moderator_Moti_CATAG

 ( 1)  1.allocation#2.Moderator_Moti_CATAG = 0
```

```
 ( 2)  1.allocation#3.Moderator_Moti_CATAG = 0
 ( 3)  1.allocation#4.Moderator_Moti_CATAG = 0
 ( 4)  1.allocation#5.Moderator_Moti_CATAG = 0
 ( 5)  1.allocation#6.Moderator_Moti_CATAG = 0

       F(  5,    89) =    1.45
            Prob > F =    0.2127

.
. *WALD

.
. regress CYBOCS_16 i.allocation i.Moderator_Moti_CATAG i.strata_age CYBOCS_b

      Source |       SS           df       MS      Number of obs   =       104
-------------+----------------------------------   F(9, 94)        =      4.79
       Model |    2336.388         9  259.598666   Prob > F        =    0.0000
    Residual |  5091.52547        94  54.1651645   R-squared       =    0.3145
-------------+----------------------------------   Adj R-squared   =    0.2489
       Total |  7427.91346       103  72.1156647   Root MSE        =    7.3597

---------------------------------------------------------------------------------------
           CYBOCS_16 | Coefficient  Std. err.      t    P>|t|     [95% conf. interval]
---------------------+-----------------------------------------------------------------
        1.allocation |  -4.152845   1.506838    -2.76   0.007    -7.144707   -1.160983
                     |
Moderator_Moti_CATAG |
                  2  |  -17.33139   8.215174    -2.11   0.038    -33.64281   -1.019973
                  3  |   -14.2026   7.899285    -1.80   0.075    -29.88681    1.481618
                  4  |  -18.94162   7.708825    -2.46   0.016    -34.24767   -3.635566
                  5  |  -18.21698   7.606266    -2.39   0.019     -33.3194   -3.114557
                  6  |   -19.3042   7.577857    -2.55   0.012    -34.35022   -4.258191
                  7  |  -21.22434   7.783796    -2.73   0.008    -36.67925   -5.769427
                     |
        1.strata_age |   3.651035   1.519701     2.40   0.018     .6336324    6.668438
            CYBOCS_b |   .7270147   .1650914     4.40   0.000      .399222    1.054807
               _cons |   17.88554    8.24647     2.17   0.033     1.511976     34.2591
---------------------------------------------------------------------------------------
```

```
.
. est store m1

.
. regress CYBOCS_16 i.allocation##i.Moderator_Moti_CATAG i.strata_age CYBOCS_b
note: 0b.allocation#1b.Moderator_Moti_CATAG identifies no observations in the sample.
note: 1.allocation#7.Moderator_Moti_CATAG omitted because of collinearity.

      Source |       SS           df       MS      Number of obs   =       104
-------------+----------------------------------   F(14, 89)       =      3.68
       Model |  2721.07548        14  194.362534   Prob > F        =    0.0001
    Residual |  4706.83798        89    52.88582   R-squared       =    0.3663
-------------+----------------------------------   Adj R-squared   =    0.2667
       Total |  7427.91346       103  72.1156647   Root MSE        =    7.2723

------------------------------------------------------------------------------------------------
                      CYBOCS_16 | Coefficient  Std. err.      t    P>|t|     [95% conf. interval]
--------------------------------+---------------------------------------------------------------
                   1.allocation |  -14.16688   4.118887    -3.44   0.001    -22.35102   -5.982735
                                |
           Moderator_Moti_CATAG |
                             2  |  -28.83799    9.38714    -3.07   0.003    -47.49004   -10.18595
                             3  |  -25.91395   9.356708    -2.77   0.007    -44.50553   -7.322372
                             4  |  -29.76646   8.851445    -3.36   0.001     -47.3541   -12.17883
                             5  |  -28.53061    8.57703    -3.33   0.001    -45.57299   -11.48823
                             6  |   -29.2877   8.626685    -3.40   0.001    -46.42874   -12.14666
                             7  |  -27.55898   8.073516    -3.41   0.001    -43.60089   -11.51708
                                |
allocation#Moderator_Moti_CATAG |
                           0 1  |          0  (empty)
                           1 2  |   14.24384   7.661259     1.86   0.066    -.9789185     29.4666
                           1 3  |   13.39985   6.518228     2.06   0.043      .448274    26.35144
                           1 4  |   12.04981   5.770071     2.09   0.040     .5848047    23.51482
                           1 5  |   11.23962    5.04977     2.23   0.029     1.205838    21.27341
                           1 6  |   10.69108   4.937005     2.17   0.033     .8813588    20.50081
                           1 7  |          0  (omitted)
                                |
```

```
                  1.strata_age |   2.705728   1.579969     1.71   0.090    -.4336361    5.845092
                      CYBOCS_b |   .7655051   .1679246     4.56   0.000     .4318424    1.099168
                         _cons |   27.09127   8.895225     3.05   0.003     9.416648    44.76589
------------------------------------------------------------------------------------------------

.
. est store m2

.
. lrtest m2 m1

Likelihood-ratio test
Assumption: m1 nested within m2

 LR chi2(5) =   8.17
Prob > chi2 = 0.1471

.
.
. mean CYBOCS_16, over(allocation Moderator_Moti_CATAG)

Mean estimation                                                      Number of obs = 104

--------------------------------------------------------------------------------------------
                                             |       Mean   Std. err.     [95% conf. interval]
---------------------------------------------+----------------------------------------------
c.CYBOCS_16@allocation#Moderator_Moti_CATAG |
                                        0 1  |          0  (no observations)
                                        0 2  |         24   3.674235      16.71302    31.28698
                                        0 3  |         22   6.110101      9.882056    34.11794
                                        0 4  |   15.83333     3.0157      9.852404    21.81426
                                        0 5  |         19   2.196588      14.64359    23.35641
                                        0 6  |   18.58333   2.541946      13.54198    23.62468
                                        0 7  |       21.6   1.484737      18.65537    24.54463
                                        1 1  |         29          .             .           .
                                        1 2  |       21.5         .5      20.50837    22.49163
                                        1 3  |       22.5   4.828733      12.92335    32.07665
                                        1 4  |   17.55556   3.245129      11.11961     23.9915
```

```
                                 1 5  |       15.2   2.091252       11.0525     19.3475
                                 1 6  |      14.65   1.642006      11.39347    17.90653
                                 1 7  |        7.8   3.367492      1.121375    14.47863
----------------------------------------------------------------------------------------

.
.
. *Moderator_alliance

.
. regress CYBOCS_16 i.allocation##c.moderator_alliance i.strata_age CYBOCS_b

      Source |       SS           df       MS      Number of obs   =        99
-------------+----------------------------------   F(5, 93)        =      5.92
       Model |  1731.32439         5  346.264879   Prob > F        =    0.0001
    Residual |   5441.5847        93  58.5116634   R-squared       =    0.2414
-------------+----------------------------------   Adj R-squared   =    0.2006
       Total |  7172.90909        98  73.1929499   Root MSE        =    7.6493

--------------------------------------------------------------------------------------------
                   CYBOCS_16 | Coefficient  Std. err.      t    P>|t|     [95% conf. interval]
-----------------------------+--------------------------------------------------------------
                1.allocation |   7.872931   15.73529     0.50   0.618    -23.37425    39.12011
          moderator_alliance |   .1810215   .4130123     0.44   0.662     -.639139    1.001182
                             |
allocation#c.moderator_alliance |
                          1  |  -.3806795   .5140077    -0.74   0.461    -1.401397    .6400379
                             |
                1.strata_age |    3.30803   1.575645     2.10   0.038     .1791116    6.436949
                    CYBOCS_b |   .6893037   .1624888     4.24   0.000     .3666331    1.011974
                       _cons |  -5.210813   12.95249    -0.40   0.688    -30.93189    20.51026
--------------------------------------------------------------------------------------------

.
. *WALD

.
. regress CYBOCS_16 i.allocation c.moderator_alliance i.strata_age CYBOCS_b
```

```
      Source |       SS           df       MS      Number of obs   =        99
-------------+----------------------------------   F(4, 94)        =      7.30
       Model |  1699.23052         4  424.80763   Prob > F        =    0.0000
    Residual |  5473.67857        94  58.2306231   R-squared       =    0.2369
-------------+----------------------------------   Adj R-squared   =    0.2044
       Total |  7172.90909        98  73.1929499   Root MSE        =    7.6309

------------------------------------------------------------------------------------
         CYBOCS_16 | Coefficient  Std. err.      t    P>|t|     [95% conf. interval]
-------------------+----------------------------------------------------------------
      1.allocation |  -3.724279   1.544064    -2.41   0.018    -6.790053   -.6585041
moderator_alliance |  -.0620848    .250057    -0.25   0.804    -.5585788    .4344092
      1.strata_age |   3.343165   1.571144     2.13   0.036     .2236215    6.462708
          CYBOCS_b |   .6987057   .1616026     4.32   0.000     .3778399    1.019571
             _cons |   1.891558   8.685443     0.22   0.828    -15.35359    19.13671
------------------------------------------------------------------------------------

.
. est store m1

.
. regress CYBOCS_16 i.allocation##c.moderator_alliance i.strata_age CYBOCS_b

      Source |       SS           df       MS      Number of obs   =        99
-------------+----------------------------------   F(5, 93)        =      5.92
       Model |  1731.32439         5  346.264879   Prob > F        =    0.0001
    Residual |   5441.5847        93  58.5116634   R-squared       =    0.2414
-------------+----------------------------------   Adj R-squared   =    0.2006
       Total |  7172.90909        98  73.1929499   Root MSE        =    7.6493

-----------------------------------------------------------------------------------------------
                    CYBOCS_16 | Coefficient  Std. err.      t    P>|t|     [95% conf. interval]
------------------------------+----------------------------------------------------------------
                 1.allocation |   7.872931   15.73529     0.50   0.618    -23.37425    39.12011
           moderator_alliance |   .1810215   .4130123     0.44   0.662     -.639139    1.001182
                              |
allocation#c.moderator_alliance |
```

```
                       1  |  -.3806795   .5140077    -0.74   0.461    -1.401397    .6400379
                          |
            1.strata_age |    3.30803   1.575645     2.10   0.038     .1791116    6.436949
                CYBOCS_b |   .6893037   .1624888     4.24   0.000     .3666331    1.011974
                   _cons |  -5.210813   12.95249    -0.40   0.688    -30.93189    20.51026
------------------------------------------------------------------------------------------

.
. est store m2

.
. lrtest m2 m1

Likelihood-ratio test
Assumption: m1 nested within m2

 LR chi2(1) =   0.58
Prob > chi2 = 0.4455

.
.
. *Moderator_compliance

.
. *Fitter ikke

.
. regress CYBOCS_16 i.allocation##c. moderator_compliance i.strata_age CYBOCS_b

      Source |       SS           df       MS      Number of obs   =       110
-------------+----------------------------------   F(5, 104)       =      9.70
       Model |  2589.01731         5  517.803463   Prob > F        =    0.0000
    Residual |  5549.74632       104  53.3629454   R-squared       =    0.3181
-------------+----------------------------------   Adj R-squared   =    0.2853
       Total |  8138.76364       109  74.6675563   Root MSE        =     7.305

--------------------------------------------------------------------------------------------
                  CYBOCS_16 | Coefficient  Std. err.      t    P>|t|     [95% conf. interval]
```

```
------------------------------+----------------------------------------------------------------
                 1.allocation |  -2.350476   7.601333    -0.31   0.758     -17.4242    12.72325
        moderator_compliance |  -2.929639    1.29796    -2.26   0.026    -5.503542   -.3557362
                              |
allocation#c.moderator_compliance |
                            1 |  -.4044347   1.603615    -0.25   0.801    -3.584464    2.775595
                              |
                 1.strata_age |   2.898468   1.400602     2.07   0.041     .1210215    5.675914
                     CYBOCS_b |   .5915928   .1494669     3.96   0.000     .2951943    .8879914
                        _cons |   16.91373    7.49981     2.26   0.026     2.041322    31.78613
-----------------------------------------------------------------------------------------------

.
. regress CYBOCS_16 i.allocation##i.MOD_COMPL_FACTOR i.strata_age CYBOCS_b
note: 0b.allocation#1b.MOD_COMPL_FACTOR identifies no observations in the sample.
note: 1.allocation#5.MOD_COMPL_FACTOR omitted because of collinearity.

      Source |       SS           df       MS      Number of obs   =       110
-------------+----------------------------------   F(10, 99)       =      5.94
       Model |  3051.77106        10  305.177106   Prob > F        =    0.0000
    Residual |  5086.99257        99  51.3837634   R-squared       =    0.3750
-------------+----------------------------------   Adj R-squared   =    0.3118
       Total |  8138.76364       109  74.6675563   Root MSE        =    7.1682

----------------------------------------------------------------------------------------
             CYBOCS_16 | Coefficient  Std. err.      t    P>|t|     [95% conf. interval]
-----------------------+----------------------------------------------------------------
          1.allocation |  -5.111022   2.007641    -2.55   0.012    -9.094617   -1.127428
                       |
      MOD_COMPL_FACTOR |
                     2 |   .0405987   9.045253     0.00   0.996    -17.90714    17.98834
                     3 |  -6.745393    6.08148    -1.11   0.270    -18.81237    5.321583
                     4 |  -14.37379   5.784487    -2.48   0.015    -25.85147   -2.896116
                     5 |  -13.28854   5.331633    -2.49   0.014    -23.86766   -2.709423
                       |
allocation#MOD_COMPL_FACTOR |
                   0 1 |          0  (empty)
                   1 2 |   -1.64924   8.324255    -0.20   0.843    -18.16637    14.86789
```

```
                  1 3  |  -.0368712   4.382917    -0.01   0.993    -8.733529    8.659787
                  1 4  |   1.706269   3.136149     0.54   0.588    -4.516531    7.929068
                  1 5  |          0  (omitted)
                       |
         1.strata_age |   2.787368   1.420145     1.96   0.052    -.0305081    5.605244
             CYBOCS_b |   .5843729   .1500406     3.89   0.000     .2866599    .8820859
                _cons |   15.80959   7.336498     2.15   0.034     1.252388     30.3668
---------------------------------------------------------------------------------------------

.
. testparm i.allocation#i.MOD_COMPL_FACTOR

 ( 1)  1.allocation#2.MOD_COMPL_FACTOR = 0
 ( 2)  1.allocation#3.MOD_COMPL_FACTOR = 0
 ( 3)  1.allocation#4.MOD_COMPL_FACTOR = 0

       F(  3,    99) =    0.13
            Prob > F =    0.9406

.
. *WALD

.
. regress CYBOCS_16 i.allocation i.MOD_COMPL_FACTOR i.strata_age CYBOCS_b

      Source |       SS           df       MS      Number of obs   =       110
-------------+----------------------------------   F(7, 102)       =      8.65
       Model |  3031.35712         7  433.051017   Prob > F        =    0.0000
    Residual |  5107.40652       102  50.0726129   R-squared       =    0.3725
-------------+----------------------------------   Adj R-squared   =    0.3294
       Total |  8138.76364       109  74.6675563   Root MSE        =    7.0762

-----------------------------------------------------------------------------------
       CYBOCS_16 | Coefficient  Std. err.      t    P>|t|     [95% conf. interval]
-----------------+-----------------------------------------------------------------
    1.allocation |   -4.56969   1.383047    -3.30   0.001    -7.312958   -1.826423
                 |
MOD_COMPL_FACTOR |
```

```
          2  |   -1.09316   5.997515    -0.18   0.856    -12.9892    10.80288
          3  |  -6.419669   5.505405    -1.17   0.246   -17.33961    4.500276
          4  |  -13.21533   5.256278    -2.51   0.013   -23.64113   -2.789525
          5  |  -12.98881   5.221794    -2.49   0.014   -23.34622   -2.631409
             |
1.strata_age |   2.941718   1.370468     2.15   0.034    .2234022    5.660035
    CYBOCS_b |   .5945257   .1470799     4.04   0.000    .3027934    .8862579
       _cons |   14.87127   7.027117     2.12   0.037    .9330195    28.80953
------------------------------------------------------------------------------

.
. est store m1

.
. regress CYBOCS_16 i.allocation##i.MOD_COMPL_FACTOR i.strata_age CYBOCS_b
note: 0b.allocation#1b.MOD_COMPL_FACTOR identifies no observations in the sample.
note: 1.allocation#5.MOD_COMPL_FACTOR omitted because of collinearity.

      Source |       SS           df       MS      Number of obs   =       110
-------------+----------------------------------   F(10, 99)       =      5.94
       Model |  3051.77106        10  305.177106   Prob > F        =    0.0000
    Residual |  5086.99257        99  51.3837634   R-squared       =    0.3750
-------------+----------------------------------   Adj R-squared   =    0.3118
       Total |  8138.76364       109  74.6675563   Root MSE        =    7.1682

-----------------------------------------------------------------------------------------------
                    CYBOCS_16 | Coefficient  Std. err.      t    P>|t|     [95% conf. interval]
------------------------------+----------------------------------------------------------------
                 1.allocation |  -5.111022   2.007641    -2.55   0.012    -9.094617   -1.127428
                              |
             MOD_COMPL_FACTOR |
                           2  |   .0405987   9.045253     0.00   0.996    -17.90714    17.98834
                           3  |  -6.745393    6.08148    -1.11   0.270    -18.81237    5.321583
                           4  |  -14.37379   5.784487    -2.48   0.015    -25.85147   -2.896116
                           5  |  -13.28854   5.331633    -2.49   0.014    -23.86766   -2.709423
                              |
allocation#MOD_COMPL_FACTOR |
                         0 1  |          0  (empty)
```

```
                 1 2  |   -1.64924   8.324255    -0.20   0.843    -18.16637    14.86789
                 1 3  |  -.0368712   4.382917    -0.01   0.993    -8.733529    8.659787
                 1 4  |   1.706269   3.136149     0.54   0.588    -4.516531    7.929068
                 1 5  |          0  (omitted)
                      |
         1.strata_age |   2.787368   1.420145     1.96   0.052    -.0305081    5.605244
             CYBOCS_b |   .5843729   .1500406     3.89   0.000     .2866599    .8820859
                _cons |   15.80959   7.336498     2.15   0.034     1.252388     30.3668
------------------------------------------------------------------------------------------

.
. est store m2

.
. lrtest m2 m1

Likelihood-ratio test
Assumption: m1 nested within m2

 LR chi2(3) =   0.44
Prob > chi2 = 0.9317

.
. mean CYBOCS_16, over(allocation MOD_COMPL_FACTOR)

Mean estimation                                           Number of obs = 110

--------------------------------------------------------------------------------------
                                        |       Mean   Std. err.     [95% conf. interval]
----------------------------------------+---------------------------------------------
c.CYBOCS_16@allocation#MOD_COMPL_FACTOR |
                                   0 1  |          0  (no observations)
                                   0 2  |         35           .             .          .
                                   0 3  |         26   1.636634      22.75624    29.24376
                                   0 4  |   17.52632   1.691687      14.17345    20.87918
                                   0 5  |   19.08696   1.756876      15.60489    22.56903
                                   1 1  |       30.5        8.5      13.65328    47.34672
                                   1 2  |      24.25   5.375484      13.59596    34.90404
```

```
                     1 3  |   20.16667    2.19722      15.81185    24.52149
                     1 4  |   15.27778   1.689656      11.92893    18.62662
                     1 5  |   13.34483   1.510453      10.35116     16.3385
--------------------------------------------------------------------------

.
.
. *KIDSCREEN

. codebook KIDSCREEN_d_16 if allocation ==0

-----------------------------------------------------------------------------------
KIDSCREEN_d_16                                                       KIDSCREEN_d_16
-----------------------------------------------------------------------------------

                  Type: Numeric (double)

                 Range: [-.971,.1]                   Units: .001
         Unique values: 11                       Missing .: 36/66

                  Mean:  -.3832
             Std. dev.: .281329

           Percentiles:     10%       25%       50%       75%       90%
                         -.7615     -.563     -.374     -.187      .002

. codebook KIDSCREEN_d_16 if allocation ==1

-----------------------------------------------------------------------------------
KIDSCREEN_d_16                                                       KIDSCREEN_d_16
-----------------------------------------------------------------------------------

                  Type: Numeric (double)

                 Range: [-.971,.199]                 Units: .001
         Unique values: 13                       Missing .: 23/64

                  Mean: -.304902
```

```
            Std. dev.:  .318525

          Percentiles:      10%       25%       50%       75%       90%
                           -.76     -.563     -.281      .002        .1

.
. ttest KIDSCREEN_d_16, by(allocation)

Two-sample t test with equal variances
------------------------------------------------------------------------------
   Group |     Obs        Mean    Std. err.   Std. dev.   [95% conf. interval]
---------+--------------------------------------------------------------------
       0 |      30      -.3832    .0513634    .2813289   -.4882499   -.2781501
       1 |      41   -.3049024    .0497453    .3185255   -.4054415   -.2043634
---------+--------------------------------------------------------------------
Combined |      71   -.3379859    .0360521    .3037804   -.4098896   -.2660823
---------+--------------------------------------------------------------------
    diff |           -.0782976    .0729056               -.2237403    .0671452
------------------------------------------------------------------------------
    diff = mean(0) - mean(1)                                      t =  -1.0740
H0: diff = 0                                     Degrees of freedom =       69

    Ha: diff < 0                 Ha: diff != 0                 Ha: diff > 0
 Pr(T < t) = 0.1433         Pr(|T| > |t|) = 0.2866          Pr(T > t) = 0.8567

.
. regress KIDSCREEN_d_16 i.allocation i.strata_age i.strata_cybocs KIDSCREEN_d_0

      Source |       SS           df       MS      Number of obs   =        62
-------------+----------------------------------   F(4, 57)        =      8.97
       Model |  2.19554404         4   .54888601   Prob > F        =    0.0000
    Residual |   3.4883759        57  .061199577   R-squared       =    0.3863
-------------+----------------------------------   Adj R-squared   =    0.3432
       Total |  5.68391994        61  .093179015   Root MSE        =    .24739

---------------------------------------------------------------------------------
 KIDSCREEN_d_16 | Coefficient  Std. err.      t    P>|t|     [95% conf. interval]
----------------+----------------------------------------------------------------
```

```
   1.allocation |   .1047722   .0637673     1.64   0.106    -.0229195     .232464
   1.strata_age |  -.1398979   .0639269    -2.19   0.033    -.2679094   -.0118865
1.strata_cybocs |  -.0050331   .0646162    -0.08   0.938    -.1344248    .1243586
  KIDSCREEN_d_0 |    .392696   .0741952     5.29   0.000     .2441226    .5412693
          _cons |  -.4122794   .0783913    -5.26   0.000    -.5692552   -.2553035
------------------------------------------------------------------------------------

.
.
. *NEQ_d_16

.
. by allocation, sort : summarize NEQ_d_16, detail

---------------------------------------------------------------------------------------------------------
-> allocation = 0

                          NEQ_d_16
-------------------------------------------------------------
      Percentiles      Smallest
 1%            0              0
 5%            0              0
10%            0              0       Obs                  38
25%            1              0       Sum of wgt.          38

50%            3                      Mean           3.868421
                        Largest       Std. dev.      3.684673
75%            6             10
90%           10             10       Variance       13.57681
95%           13             13       Skewness       1.127532
99%           14             14       Kurtosis       3.521707

---------------------------------------------------------------------------------------------------------
-> allocation = 1

                          NEQ_d_16
-------------------------------------------------------------
      Percentiles      Smallest
```

```
 1%            0              0
 5%            0              0
10%            0              0       Obs                  47
25%            1              0       Sum of wgt.          47

50%            3                      Mean           3.170213
                        Largest       Std. dev.      2.891661
75%            5              7
90%            7              7       Variance       8.361702
95%            7              9       Skewness        .957295
99%           13             13       Kurtosis       4.070753

.
. by allocation, sort : inspect NEQ_d_16

----------------------------------------------------------------------------------------------------
-> allocation = 0

NEQ_d_16:  NEQ_d_16                             Number of observations
-------------------                    ---------------------------------------
                                             Total      Integers   Nonintegers
|  #                        Negative             -             -             -
|  #                        Zero                 6             6             -
|  #                        Positive            32            32             -
|  #   #                               -----------   -----------   -----------
|  #   #                    Total               38            38             -
|  #   #   #   #   .        Missing             28
+----------------------                -----------
0                     14                        66
  (13 unique values)

----------------------------------------------------------------------------------------------------
-> allocation = 1

NEQ_d_16:  NEQ_d_16                             Number of observations
-------------------                    ---------------------------------------
                                             Total      Integers   Nonintegers
```

```
|  #                          Negative            -             -             -
|  #                          Zero               11            11             -
|  #                          Positive           36            36             -
|  #   #                               -----------   -----------   -----------
|  #   #   #                  Total              47            47             -
|  #   #   #   .   .          Missing            17
+----------------------                ----------- 
0                    13                          64
  (10 unique values)


.
. vanelteren NEQ_d_16, by(allocation) strata(strata_cybocs)

Generalized Wilcoxon-Mann-Whitney Ranksum Test (van Elteren's Test)


                                              Variance of
                      Weighted    Expected      Weighted
   Stratum    |   n   | Ranksum   | Ranksum  |   Ranksum
-------------+-------+-----------+----------+-------------
           0 |   34  |    6.27   |    7.0   |    0.656
           1 |   51  |   13.30   |   12.0   |    1.011
-------------+-------+-----------+----------+-------------
    Sums     |   85  |   19.57   |   19.0   |    1.666

Asymptotic test statistic
z =   .4412
Prob(Z > |z|) =  .6591

.
. cendif NEQ_d_16,by(allocation) level(95)
Y-variable: NEQ_d_16 (NEQ_d_16)
Grouped by: allocation (allocation)
Group numbers:

 allocation |      Freq.     Percent        Cum.
------------+-----------------------------------
```

```
          0 |         38       44.71       44.71
          1 |         47       55.29      100.00
------------+-----------------------------------
      Total |         85      100.00
Transformation: Fisher's z
95% confidence interval(s) for percentile difference(s)
between values of NEQ_d_16 in first and second groups:
   Percent    Pctl_Dif     Minimum     Maximum
        50           0          -1           2

.
.
.
. log close
      name:  <unnamed>
       log:  /Users/janusjakobsen/Documents/OUTPUT_2.log
  log type:  text
 closed on:  28 Oct 2022, 16:15:00
----------------------------------------------------------------------------------------------------
```

Statistical report for exploratory analyses from the TECTO-trial

Markus Harboe Olsen

Invalid Date

Table of contents

Exploratory outcomes **3**
CY-BOCS - mixed methods 3
KIDSCREEN-10 5
Assumptions for linear regression 5
Analysis 6
KIDSCREEN - participants - mixed methods 6
Kidscreen-10 - parents 9
Assumptions for linear regression 9
Summary data 10
Analysis 10
NEQ - parents 12
Assumptions for linear regression 12
Summary data 13
Analysis 13
Serious adverse events 14
Summary data 14
Analysis 14
COIS-R - participants 15
Assumptions for linear regression 15
Summary data 16
Analysis 16
COIS-R - parents 17
Assumptions for linear regression 17
Summary data 18

Analysis 18
CGI-S 19
Assumptions for linear regression 19
Summary data 20
Analysis 20
CGI-I 21
Assumptions for linear regression 21
Summary data 22
Analysis 22
CGAS 23
Assumptions for linear regression 23
Summary data 24
Analysis 24
Response rate 25
Summary data 25
Analysis 25
Remission rate 26
Summary data 26
Analysis 26
Suicidality 27
Summary data 27
Analysis 27
TOCS - participants 28
Assumptions for linear regression 28
Summary data 29
Analysis 29
TOCS - parents 30
Assumptions for linear regression 30
Summary data 31
Analysis 31
FAS-PR 32
Assumptions for linear regression 32
Summary data 33
Analysis 33
PSS 34
Assumptions for linear regression 34
Summary data 35
Analysis 35

Exploratory outcomes

CY-BOCS - mixed methods

```
Linear mixed-effects model fit by REML
  Data: df_mm
       AIC      BIC    logLik
  2975.061 2995.857 -1482.531

Random effects:
 Formula: ~1 | ssid
        (Intercept) Residual
StdDev:    4.387707 4.433816

Fixed effects:  measurement ~ time + intervention
                    Value Std.Error  DF   t-value p-value
(Intercept)     26.081908 0.5062725 344  51.51753  0.0000
time            -0.445627 0.0392049 344 -11.36663  0.0000
intervention1   -1.744477 0.6491919 344  -2.68715  0.0076
 Correlation:
              (Intr) time
time          -0.301
intervention1 -0.222 -0.452

Standardized Within-Group Residuals:
         Min           Q1          Med           Q3          Max
-4.527017485 -0.441361611  0.004774205  0.561427176  2.642475902

Number of Observations: 476
Number of Groups: 130


             numDF denDF   F-value p-value
(Intercept)      1   344 2656.0605  <.0001
time             1   344  198.8468  <.0001
intervention     1   344    7.2208  0.0076
```

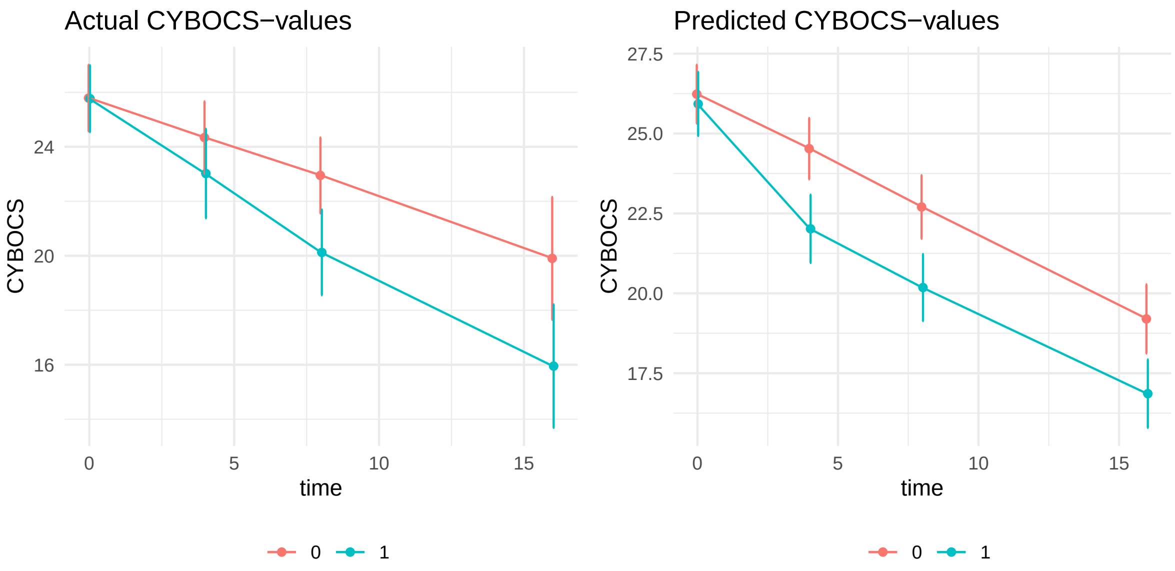
Actual CYBOCS−values
CYBOCS
24
20
16
0
5
10
15
time
0
1
Predicted CYBOCS−values
CYBOCS
27.5
25.0
22.5
20.0
17.5
0
5
10
15
time
0
1

KIDSCREEN-10

Assumptions for linear regression

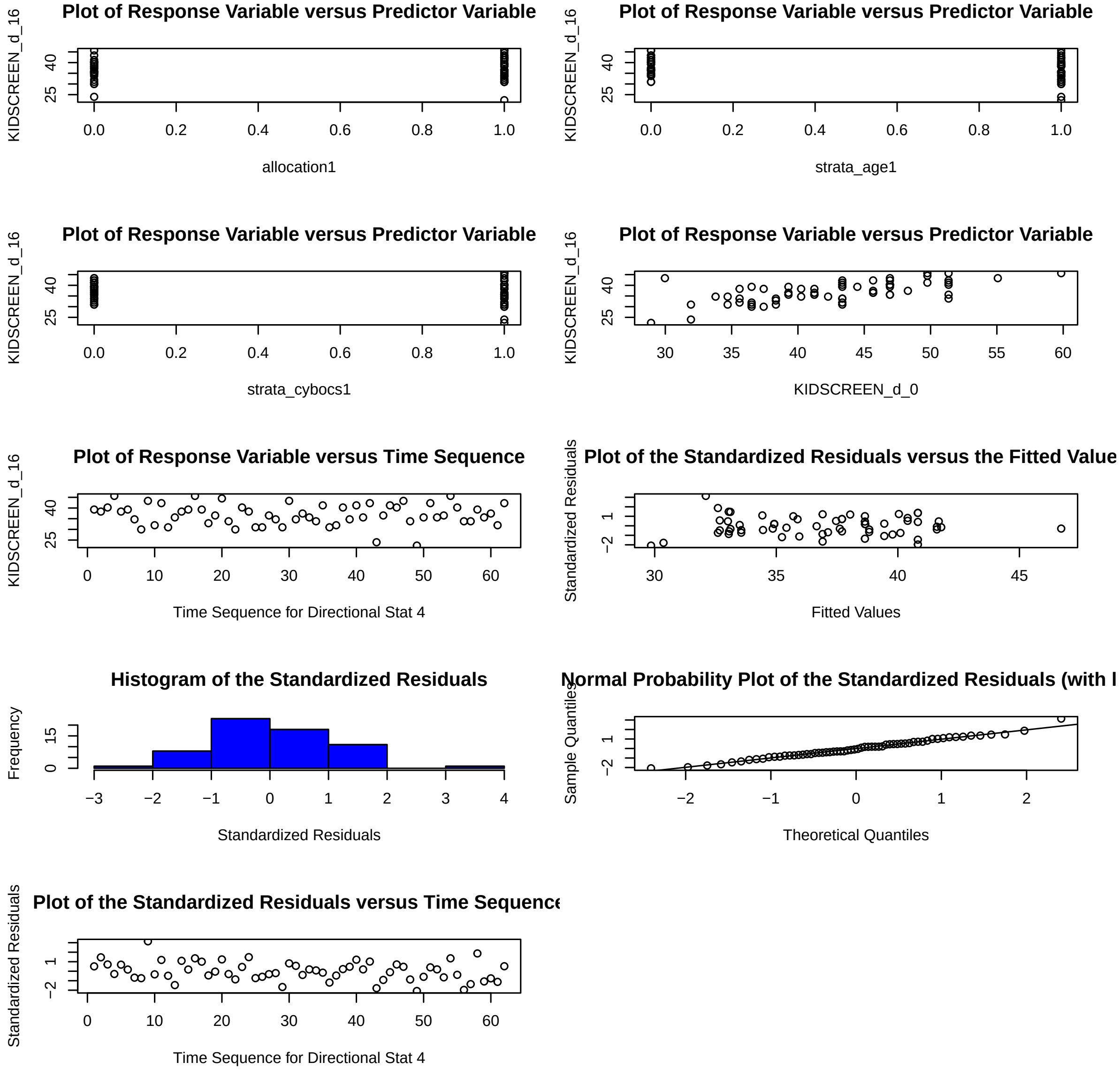

	Value	p-value	Decision
Global Stat	2.0015445	0.7354748	Assumptions acceptable.
Skewness	0.9091808	0.3403319	Assumptions acceptable.

	Value	p-value	Decision
Kurtosis	0.2669934	0.6053558	Assumptions acceptable.
Link Function	0.8102135	0.3680571	Assumptions acceptable.
Heteroscedasticity	0.0151567	0.9020180	Assumptions acceptable.

Analysis

	KIDSCREEN_d_16		
Predictors	Estimates	CI	p
(Intercept)	16.49	8.96 – 24.02	**<0.001**
allocation [1]	0.96	-0.97 – 2.88	0.323
strata age [1]	-1.74	-3.70 – 0.22	0.081
strata cybocs [1]	-0.00	-1.99 – 1.99	0.999
KIDSCREEN d 0	0.49	0.33 – 0.64	**<0.001**
Observations	62		
R^2 / R^2 adjusted	0.470 / 0.433		

KIDSCREEN - participants - mixed methods

```
Linear mixed-effects model fit by REML
  Data: df_mm
       AIC      BIC    logLik
  2033.954 2053.098 -1011.977

Random effects:
 Formula: ~1 | ssid
        (Intercept) Residual
StdDev:    5.121854 3.264693

Fixed effects:  measurement ~ time + intervention
                 Value Std.Error  DF   t-value p-value
(Intercept)   42.29094 0.5553357 225  76.15383       0
time          -0.45270 0.0374505 225 -12.08800       0
intervention1  2.85443 0.5967824 225   4.78303       0
 Correlation:
              (Intr) time
time          -0.214
```

```
intervention1 -0.173 -0.493

Standardized Within-Group Residuals:
        Min          Q1         Med          Q3         Max 
-3.42390400 -0.49989752 -0.02466003  0.43053942  3.09500410 

Number of Observations: 343
Number of Groups: 116 

             numDF denDF  F-value p-value
(Intercept)      1   225 6281.363  <.0001
time             1   225  125.079  <.0001
intervention     1   225   22.877  <.0001
```

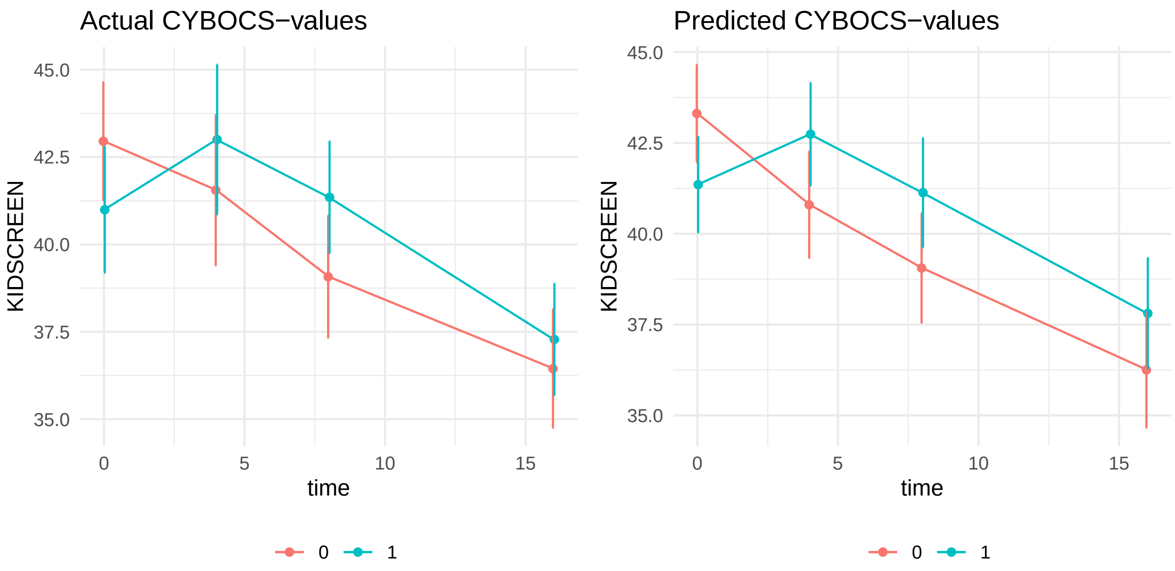
Actual CYBOCS−values
Predicted CYBOCS−values
KIDSCREEN
time
45.0
42.5
40.0
37.5
35.0
0
5
10
15
0
1

Kidscreen-10 - parents

Assumptions for linear regression

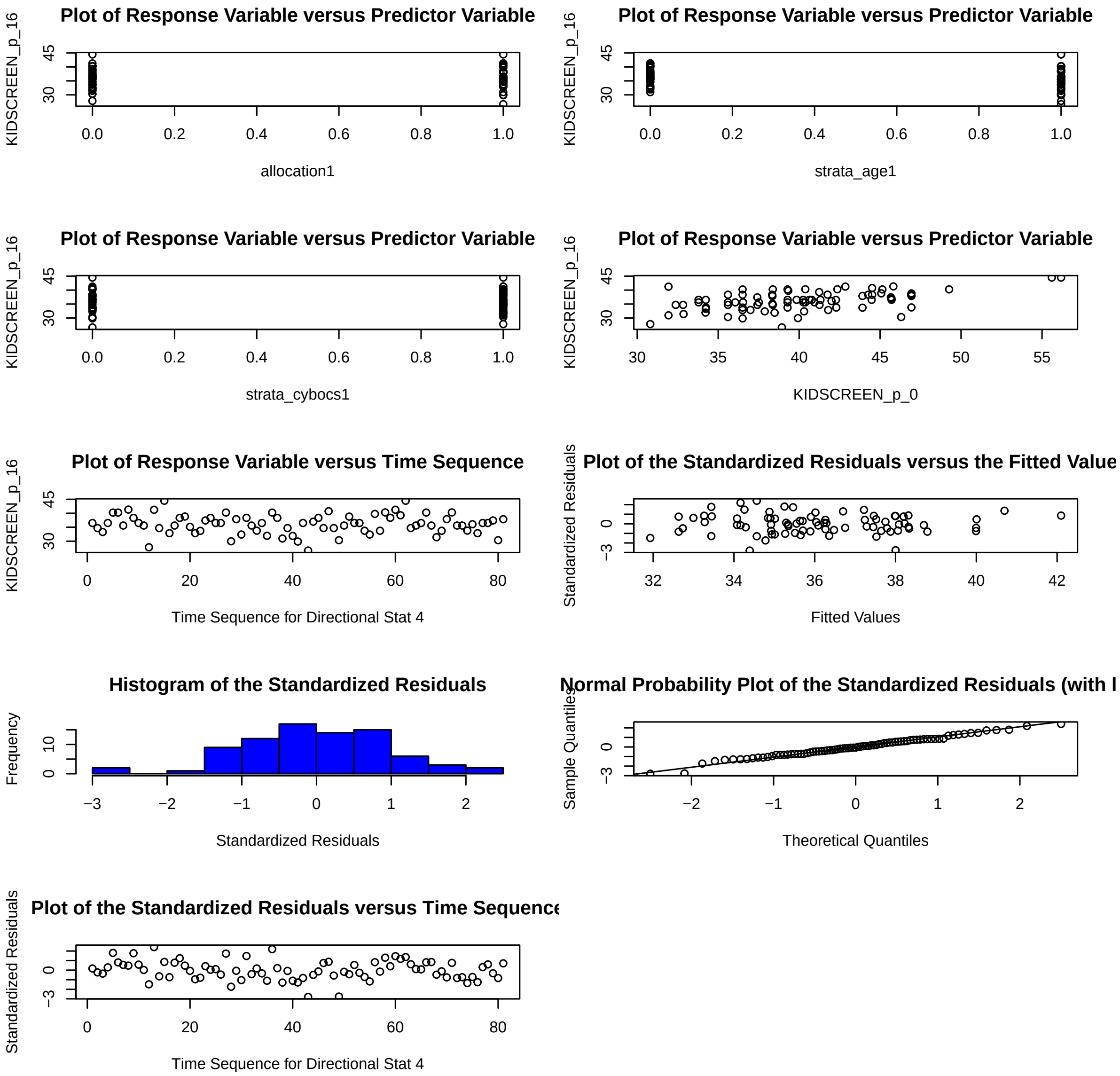

	Value	p-value	Decision
Global Stat	1.3713631	0.8491567	Assumptions acceptable.
Skewness	0.1857745	0.6664570	Assumptions acceptable.
Kurtosis	0.2845329	0.5937455	Assumptions acceptable.
Link Function	0.4338546	0.5101036	Assumptions acceptable.
Heteroscedasticity	0.4672011	0.4942776	Assumptions acceptable.

Summary data

	0	1	Overall
	(N=66)	(N=64)	(N=130)
**KIDSCREEN__p__0**			
Mean (SD) [Min; Max]	39.1 (5.02) [30.8; 56.2]	39.2 (4.88) [27.0; 55.6]	39.1 (4.93) [27.0; 56.2]
Missing	6 (9.1%)	8 (12.5%)	14 (10.8%)
**KIDSCREEN__p__4**			
Mean (SD) [Min; Max]	39.5 (4.48) [30.9; 49.8]	39.5 (4.89) [22.4; 50.4]	39.5 (4.67) [22.4; 50.4]
Missing	15 (22.7%)	13 (20.3%)	28 (21.5%)
**KIDSCREEN__p__8**			
Mean (SD) [Min; Max]	37.2 (4.45) [28.1; 49.1]	38.9 (4.31) [25.9; 50.7]	38.0 (4.44) [25.9; 50.7]
Missing	17 (25.8%)	12 (18.8%)	29 (22.3%)
**KIDSCREEN__p__16**			
Mean (SD) [Min; Max]	35.7 (3.76) [26.6; 44.5]	36.0 (3.44) [26.6; 44.5]	35.8 (3.58) [26.6; 44.5]
Missing	24 (36.4%)	20 (31.3%)	44 (33.8%)

Analysis

	KIDSCREEN__p__16		
Predictors	Estimates	CI	p
(Intercept)	20.42	14.87 – 25.97	**<0.001**
allocation [1]	0.45	-0.82 – 1.73	0.483
strata age [1]	-1.74	-3.02 – -0.47	**0.008**
strata cybocs [1]	1.17	-0.15 – 2.49	0.080
KIDSCREEN p 0	0.39	0.26 – 0.52	**<0.001**
Observations	81		

	KIDSCREEN_p_16
R^2 / R^2 adjusted	0.347<br>/<br>0.313

NEQ - parents

Assumptions for linear regression

Plot of Response Variable versus Predictor Variable

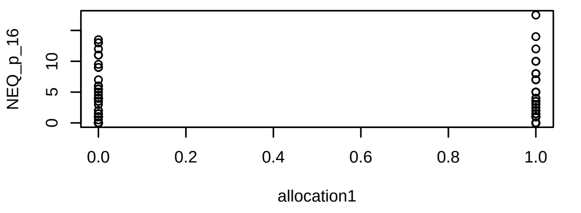

Plot of Response Variable versus Predictor Variable

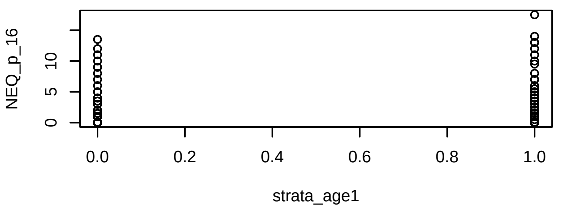

Plot of Response Variable versus Predictor Variable

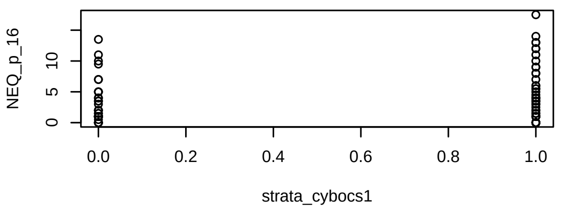

Plot of Response Variable versus Time Sequence

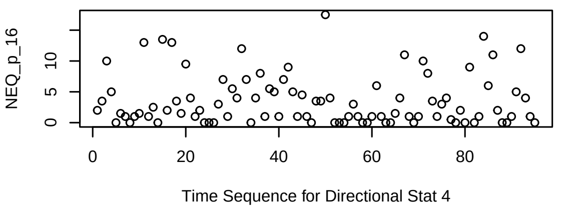

Plot of the Standardized Residuals versus the Fitted Value

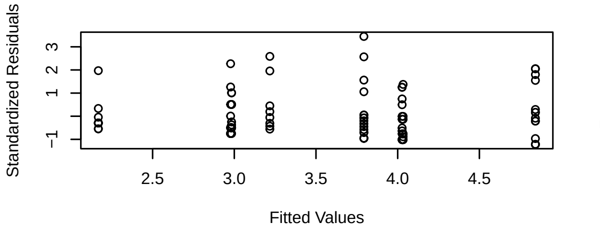

Histogram of the Standardized Residuals

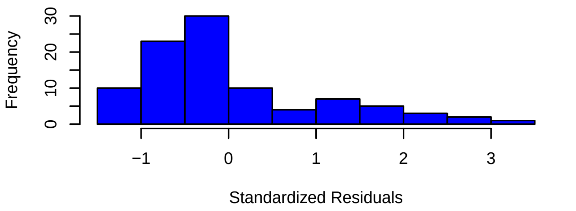

Normal Probability Plot of the Standardized Residuals (with l

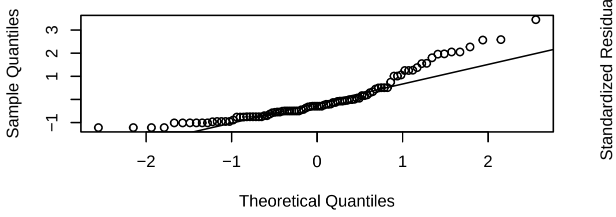

Plot of the Standardized Residuals versus Time Sequence

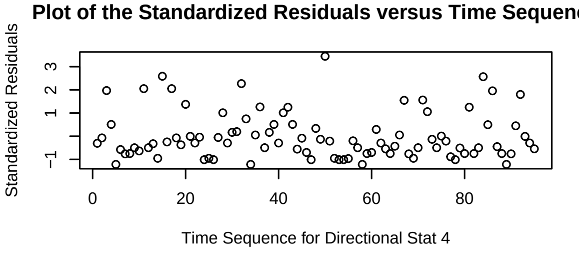

	Value	p-value	Decision
Global Stat	29.5942580	0.0000059	Assumptions NOT satisfied!
Skewness	25.2881584	0.0000005	Assumptions NOT satisfied!
Kurtosis	4.2131355	0.0401121	Assumptions NOT satisfied!
Link Function	0.0251910	0.8738920	Assumptions acceptable.
Heteroscedasticity	0.0677731	0.7946071	Assumptions acceptable.

Summary data

	0	1	Overall
	(N=66)	(N=64)	(N=130)
**NEQ__p__4**			
Median [Q1, Q3]	3.00 [1.00, 7.63]	3.00 [1.00, 6.00]	3.00 [1.00, 7.00]
Missing	14 (21.2%)	17 (26.6%)	31 (23.8%)
**NEQ__p__8**			
Median [Q1, Q3]	4.00 [2.00, 7.75]	2.00 [0, 5.00]	3.00 [1.00, 6.00]
Missing	12 (18.2%)	15 (23.4%)	27 (20.8%)
**NEQ__p__16**			
Median [Q1, Q3]	3.50 [0.875, 6.00]	1.00 [1.00, 4.00]	2.00 [1.00, 5.00]
Missing	22 (33.3%)	13 (20.3%)	35 (26.9%)

Analysis

```
    Wilcoxon rank sum test with continuity correction

data:  NEQ_p_16 by allocation
W = 1294, p-value = 0.1957
alternative hypothesis: true location shift is not equal to 0
95 percent confidence interval:
 -4.460508e-05  2.000004e+00
sample estimates:
difference in location
             0.5000319
```

Serious adverse events

Summary data

	0	1
	(N=66)	(N=64)
**as.factor(sae)**		
0	63 (95.5%)	62 (96.9%)
1	3 (4.5%)	2 (3.1%)

Analysis

Relative risk derived by G-computation RR: 0.7005 (sRR: 0.6942) Confidence interval: 0 - 202135745.0599 p-value: 0.6832

COIS-R - participants

Assumptions for linear regression

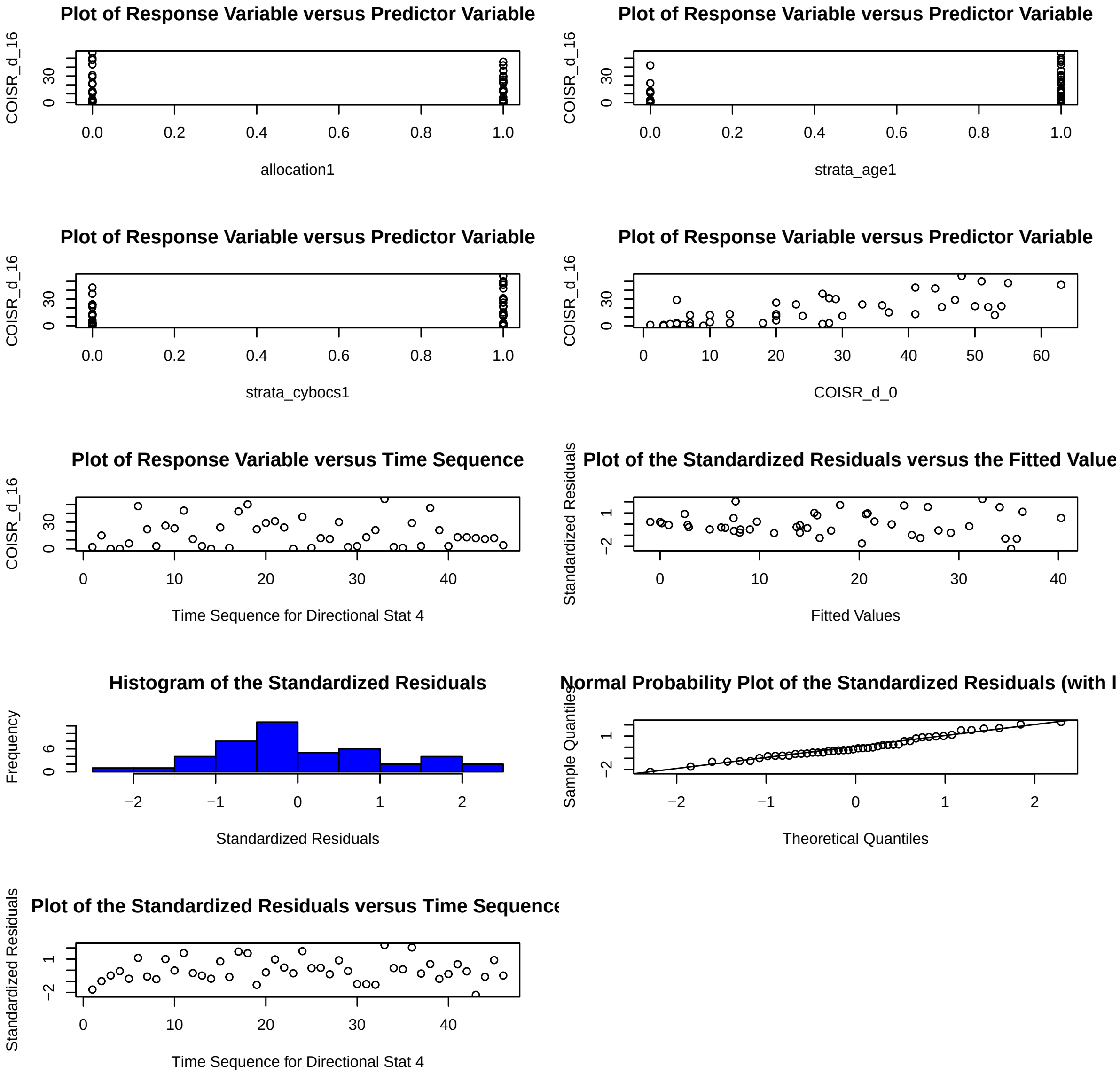

	Value	p-value	Decision
Global Stat	1.0392165	0.9037923	Assumptions acceptable.
Skewness	0.6920984	0.4054516	Assumptions acceptable.
Kurtosis	0.2041199	0.6514158	Assumptions acceptable.
Link Function	0.0052375	0.9423068	Assumptions acceptable.
Heteroscedasticity	0.1377607	0.7105174	Assumptions acceptable.

Summary data

	0	1	Overall
	(N=50)	(N=47)	(N=97)
**COISR_d_0**			
Mean (SD) [Min; Max]	29.3 (19.3) [1.00; 70.0]	26.5 (16.4) [3.00; 63.0]	28.0 (18.0) [1.00; 70.0]
Missing	10 (20.0%)	14 (29.8%)	24 (24.7%)
**COISR_d_4**			
Mean (SD) [Min; Max]	31.7 (20.6) [1.00; 82.0]	23.0 (14.0) [0; 58.0]	27.1 (17.8) [0; 82.0]
Missing	19 (38.0%)	11 (23.4%)	30 (30.9%)
**COISR_d_8**			
Mean (SD) [Min; Max]	23.1 (17.6) [2.00; 66.0]	20.6 (14.6) [0; 52.0]	21.8 (16.0) [0; 66.0]
Missing	22 (44.0%)	15 (31.9%)	37 (38.1%)
**COISR_d_16**			
Mean (SD) [Min; Max]	18.2 (17.1) [0; 56.0]	16.9 (15.1) [0; 47.0]	17.5 (15.9) [0; 56.0]
Missing	26 (52.0%)	18 (38.3%)	44 (45.4%)

Analysis

	COISR_d_16		
Predictors	Estimates	CI	p
(Intercept)	-1.56	-9.67 – 6.55	0.700
allocation [1]	-0.74	-7.42 – 5.93	0.823
strata age [1]	4.82	-2.85 – 12.49	0.212
strata cybocs [1]	1.45	-5.71 – 8.60	0.685
COISR d 0	0.58	0.37 – 0.78	**<0.001**
Observations	46		
R^2 / R^2 adjusted	0.546 / 0.502		

COIS-R - parents

Assumptions for linear regression

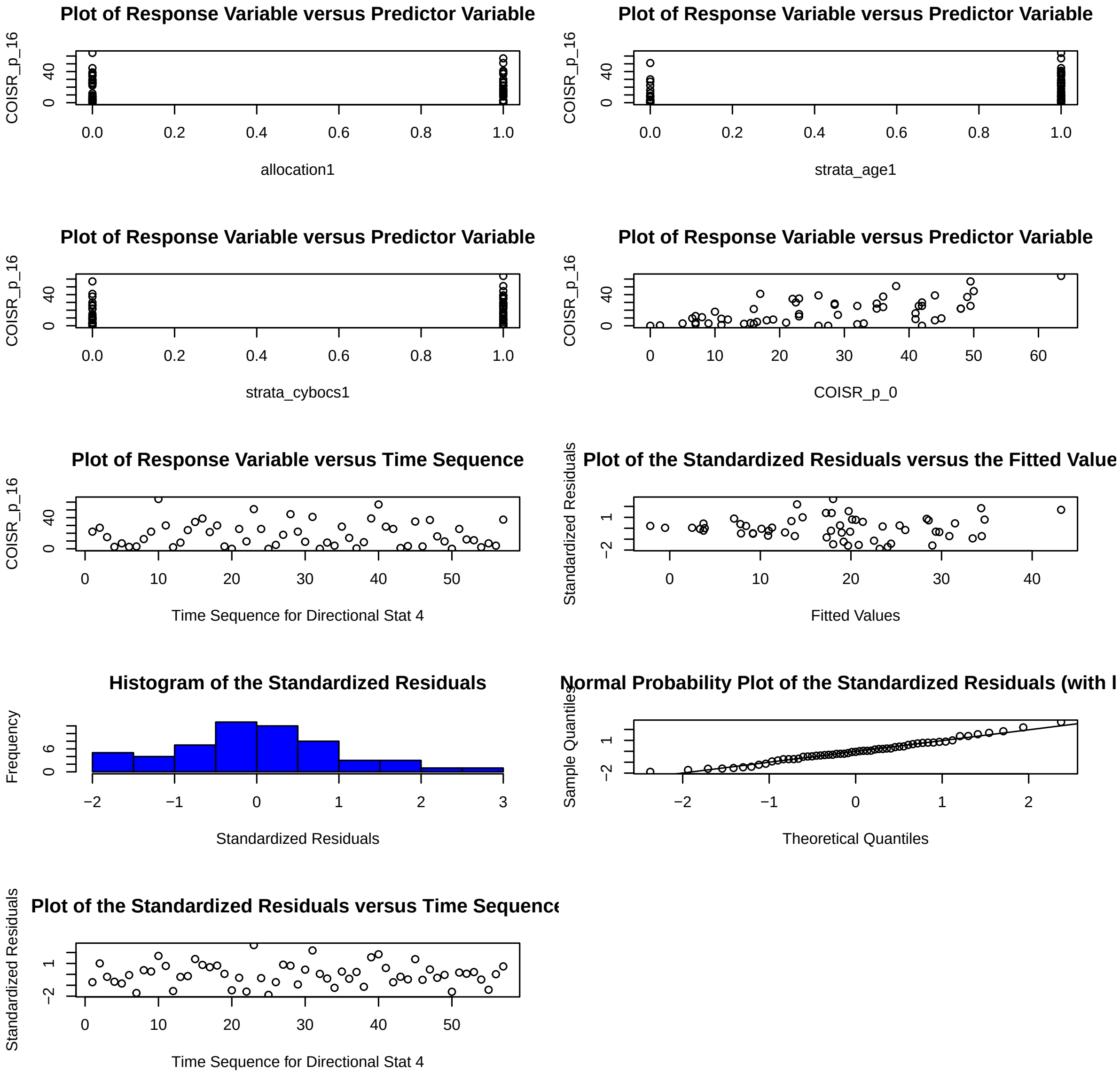

	Value	p-value	Decision
Global Stat	3.3463634	0.5016201	Assumptions acceptable.
Skewness	1.0424848	0.3072443	Assumptions acceptable.
Kurtosis	0.0159875	0.8993825	Assumptions acceptable.
Link Function	1.8344781	0.1755995	Assumptions acceptable.
Heteroscedasticity	0.4534129	0.5007186	Assumptions acceptable.

Summary data

	0	1	Overall
	(N=50)	(N=47)	(N=97)
**COISR__p__0**			
Mean (SD) [Min; Max]	32.2 (20.9) [0; 88.0]	29.4 (17.3) [7.00; 88.5]	30.9 (19.2) [0; 88.5]
Missing	9 (18.0%)	11 (23.4%)	20 (20.6%)
**COISR__p__4**			
Mean (SD) [Min; Max]	27.9 (18.7) [0.500; 77.0]	26.6 (14.3) [4.50; 55.0]	27.3 (16.6) [0.500; 77.0]
Missing	13 (26.0%)	12 (25.5%)	25 (25.8%)
**COISR__p__8**			
Mean (SD) [Min; Max]	25.4 (18.3) [0.500; 85.0]	23.3 (15.2) [3.50; 77.0]	24.3 (16.7) [0.500; 85.0]
Missing	11 (22.0%)	9 (19.1%)	20 (20.6%)
**COISR__p__16**			
Mean (SD) [Min; Max]	19.3 (16.2) [0; 64.0]	18.0 (15.1) [0; 57.0]	18.6 (15.5) [0; 64.0]
Missing	18 (36.0%)	13 (27.7%)	31 (32.0%)

Analysis

	COISR__p__16		
Predictors	Estimates	CI	p
(Intercept)	-0.51	-9.79 – 8.77	0.912
allocation [1]	-2.64	-9.68 – 4.40	0.455
strata age [1]	6.58	-1.01 – 14.17	0.088
strata cybocs [1]	-2.57	-10.44 – 5.29	0.514
COISR p 0	0.63	0.37 – 0.88	**<0.001**
Observations	57		
R^2 / R^2 adjusted	0.395 / 0.349		

CGI-S

Assumptions for linear regression

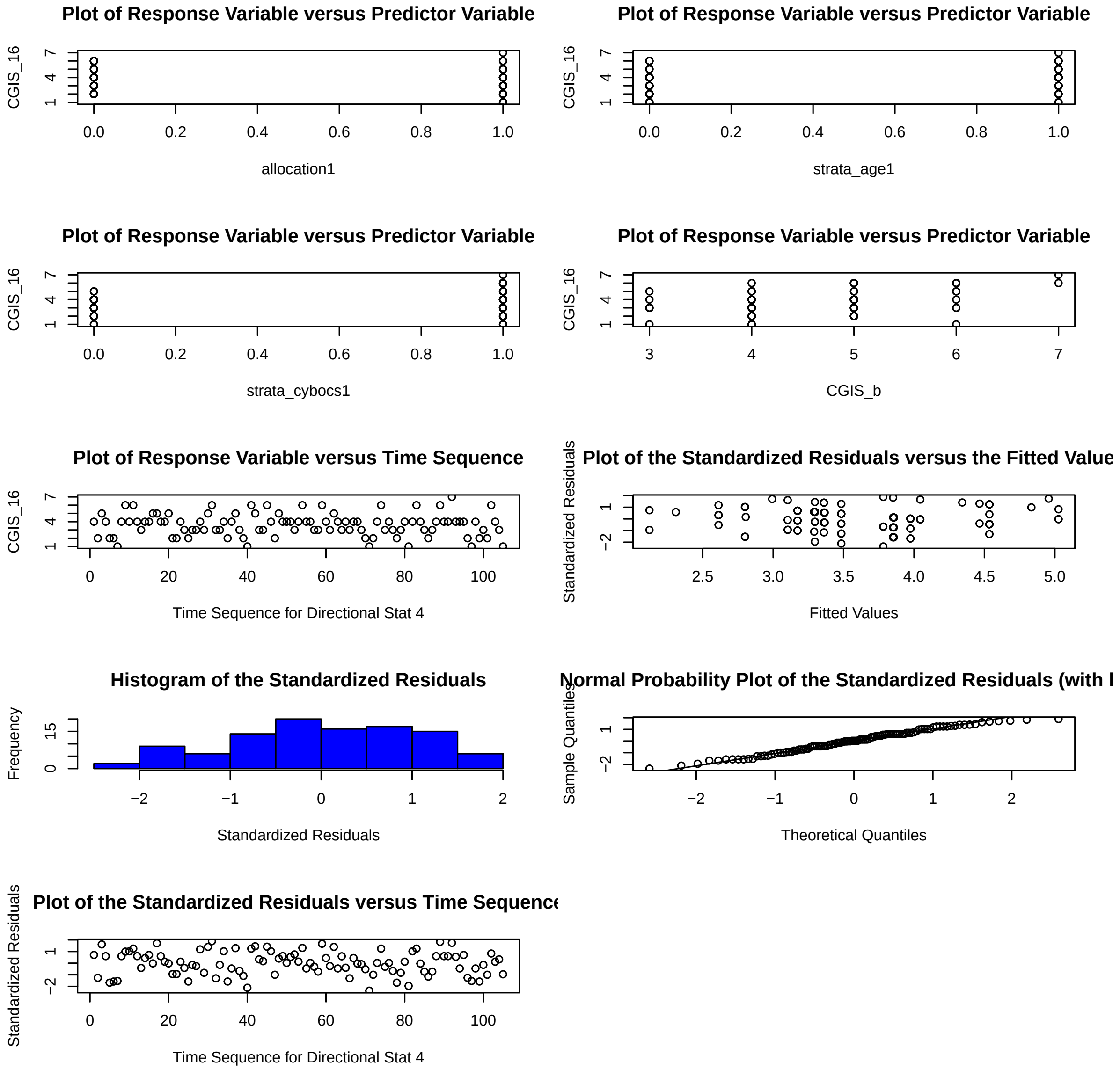

	Value	p-value	Decision
Global Stat	6.8833408	0.1421831	Assumptions acceptable.
Skewness	0.4217116	0.5160842	Assumptions acceptable.
Kurtosis	2.3925092	0.1219178	Assumptions acceptable.
Link Function	3.8599158	0.0494528	Assumptions NOT satisfied!
Heteroscedasticity	0.2092042	0.6473918	Assumptions acceptable.

Summary data

allocation	N	CGIS_b	sd
0	64	4.593750	0.8303518
1	63	4.507936	0.8775519

	0	1	Overall
	(N=66)	(N=64)	(N=130)
**CGIS_4**			
Mean (SD) [Min; Max]	4.47 (0.900) [3.00; 7.00]	4.16 (0.960) [2.00; 7.00]	4.32 (0.938) [2.00; 7.00]
Missing	4 (6.1%)	7 (10.9%)	11 (8.5%)
**CGIS_8**			
Mean (SD) [Min; Max]	4.25 (0.856) [2.00; 6.00]	3.88 (0.965) [1.00; 6.00]	4.07 (0.926) [1.00; 6.00]
Missing	6 (9.1%)	7 (10.9%)	13 (10.0%)
**CGIS_16**			
Mean (SD) [Min; Max]	3.90 (1.33) [2.00; 6.00]	3.33 (1.31) [1.00; 7.00]	3.60 (1.34) [1.00; 7.00]
Missing	16 (24.2%)	7 (10.9%)	23 (17.7%)

Analysis

	CGIS_16		
Predictors	Estimates	CI	p
(Intercept)	1.21	-0.20 – 2.62	0.091
allocation [1]	-0.56	-1.03 – -0.09	**0.019**
strata age [1]	0.68	0.21 – 1.15	**0.005**
strata cybocs [1]	0.19	-0.37 – 0.75	0.507
CGIS b	0.49	0.17 – 0.81	**0.003**
Observations	105		
R^2 / R^2 adjusted	0.232 / 0.201		

CGI-I

Assumptions for linear regression

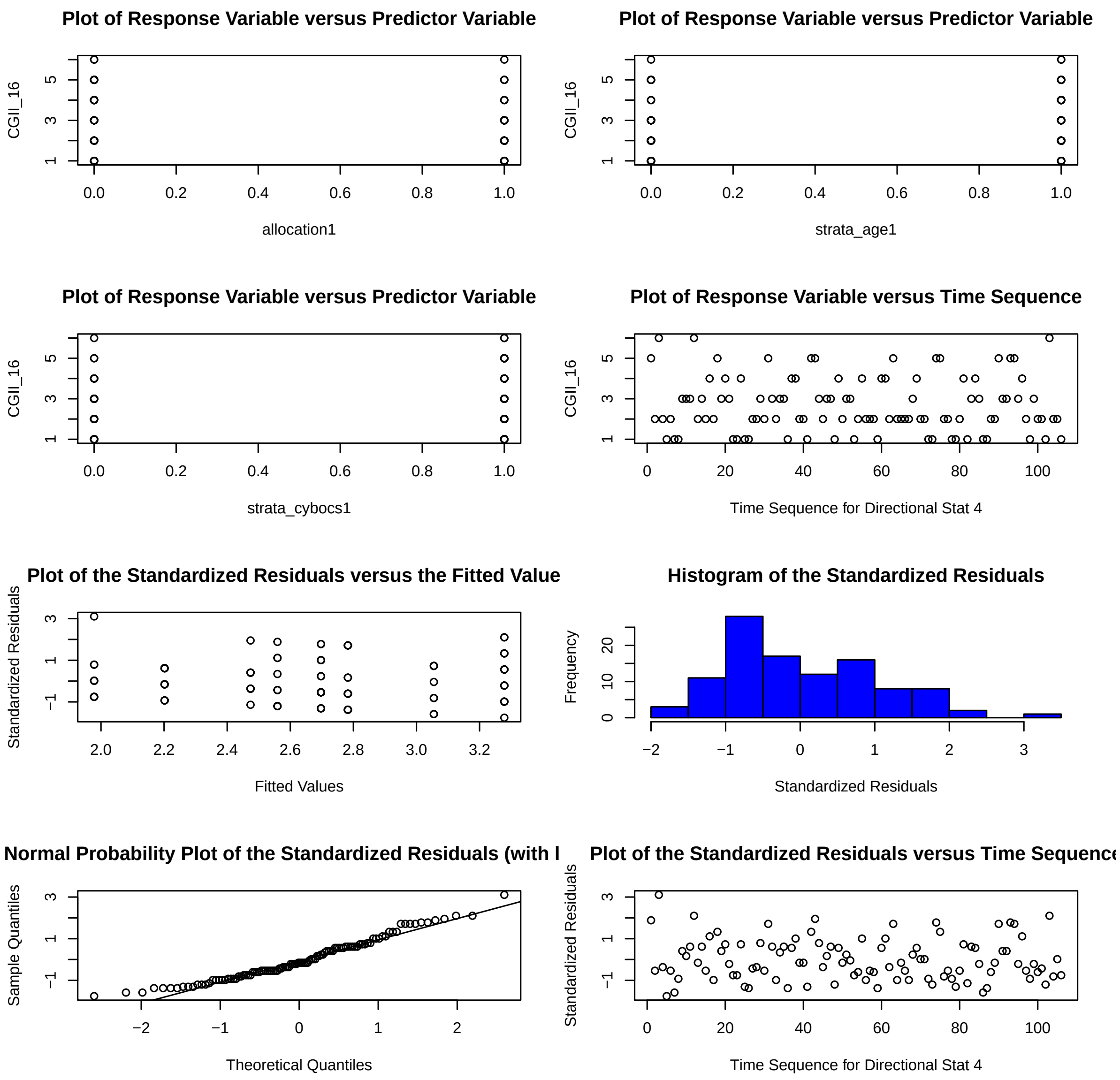

	Value	p-value	Decision
Global Stat	7.7312003	0.1019360	Assumptions acceptable.
Skewness	5.7842554	0.0161703	Assumptions NOT satisfied!
Kurtosis	0.1808687	0.6706278	Assumptions acceptable.
Link Function	0.7443818	0.3882608	Assumptions acceptable.
Heteroscedasticity	1.0216944	0.3121174	Assumptions acceptable.

Summary data

	0	1	Overall
	(N=66)	(N=64)	(N=130)
**CGII_4**			
Median [Q1,Q3]	4.00 [3.00,4.00]	4.00 [3.00,4.00]	4.00 [3.00,4.00]
Missing	4 (6.1%)	7 (10.9%)	11 (8.5%)
**CGII_8**			
Median [Q1,Q3]	3.00 [3.00,4.00]	3.00 [2.00,4.00]	3.00 [2.00,4.00]
Missing	6 (9.1%)	7 (10.9%)	13 (10.0%)
**CGII_16**			
Median [Q1,Q3]	3.00 [2.00,4.00]	2.00 [2.00,3.00]	2.00 [2.00,3.75]
Missing	15 (22.7%)	9 (14.1%)	24 (18.5%)

Analysis

```
    Wilcoxon rank sum test with continuity correction

data:  CGII_16 by allocation
W = 1711, p-value = 0.04493
alternative hypothesis: true location shift is not equal to 0
95 percent confidence interval:
 0.0000464026 1.0000634590
sample estimates:
difference in location
             0.9999821
```

CGAS

Assumptions for linear regression

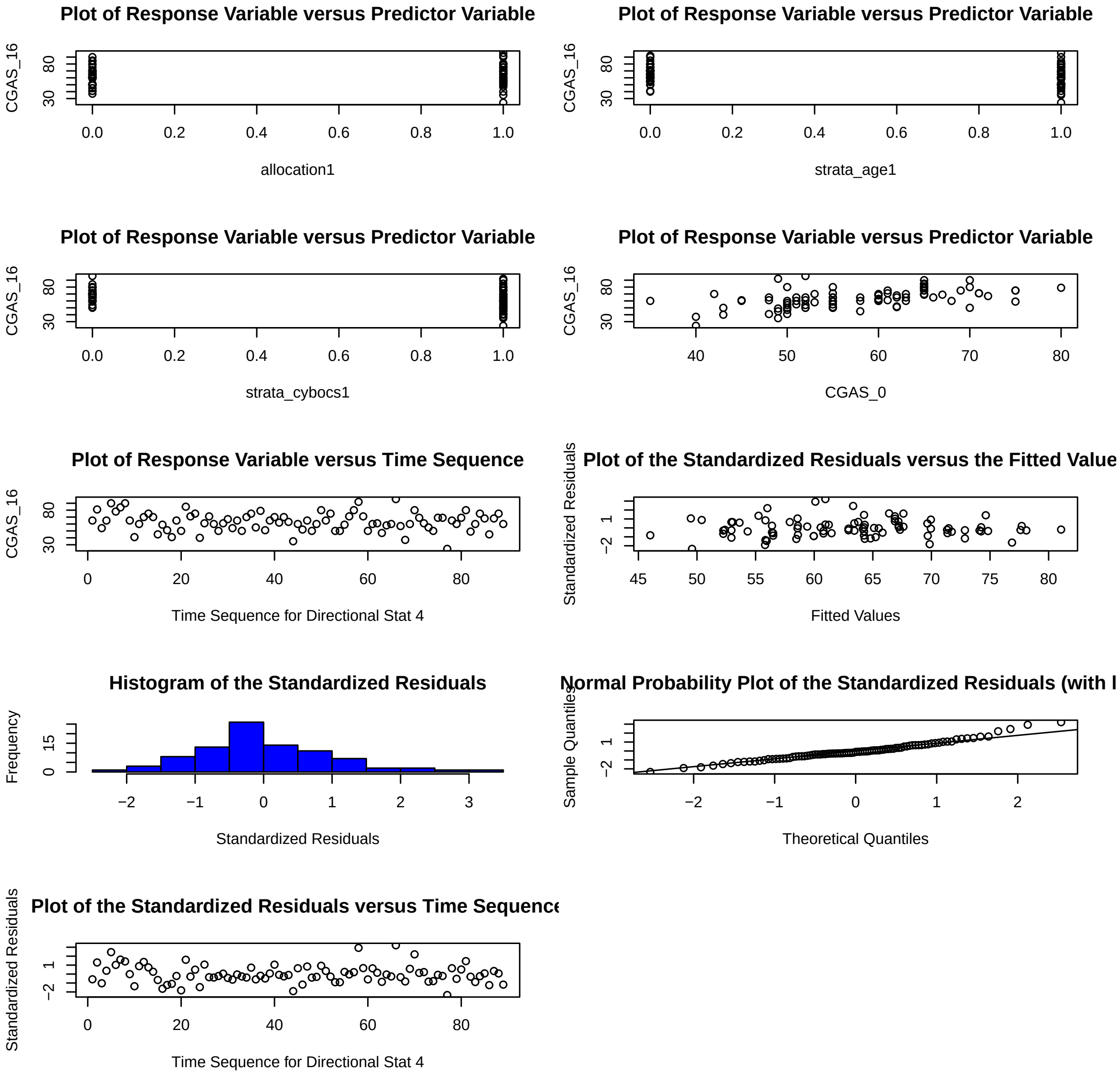

	Value	p-value	Decision
Global Stat	13.6854305	0.0083698	Assumptions NOT satisfied!
Skewness	6.4165715	0.0113060	Assumptions NOT satisfied!
Kurtosis	4.1390448	0.0419048	Assumptions NOT satisfied!
Link Function	2.9155323	0.0877305	Assumptions acceptable.
Heteroscedasticity	0.2142819	0.6434318	Assumptions acceptable.

Summary data

	**0**<br>*n = 66*	**1**<br>*n = 64*
CGAS_0		
mean (95%CI)	55.55 (53.20;57.89)	56.08 (53.66;58.49)
median (min, max)	55.00 (39.00, 80.00)	55.00 (35.00, 75.00)
CGAS_16		
mean (95%CI)	61.17 (57.15;65.20)	64.78 (60.79;68.76)
median (min, max)	61.00 (37.00, 90.00)	65 (24.00, 96.00)
missing	26 (39.4%)	15 (23.4%)

Analysis

```
    Wilcoxon rank sum test with continuity correction

data:  CGAS_16 by allocation
W = 806, p-value = 0.1516
alternative hypothesis: true location shift is not equal to 0
95 percent confidence interval:
  -9.9999997   0.9999526
sample estimates:
difference in location
             -4.999952
```

Response rate

Summary data

	0	1
	(N=66)	(N=64)
**as.factor(responders)**		
0	35 (53.0%)	29 (45.3%)
1	17 (25.8%)	30 (46.9%)
Missing	14 (21.2%)	5 (7.8%)

Analysis

Relative risk derived by G-computation RR: 1.58 (sRR: 1.6) Confidence interval: 1.01 - 2.67 p-value: 0.0428 It took 76 seconds.

Remission rate

Summary data

	0	1
	(N=66)	(N=64)
**as.factor(remission)**		
0	58 (87.9%)	50 (78.1%)
1	8 (12.1%)	14 (21.9%)

Analysis

Relative risk derived by G-computation RR: 1.81 (sRR: 1.83) Confidence interval: 0.83 - 5 p-value: 0.1385 It took 68 seconds.

Suicidality

Summary data

	0	1
	(N=66)	(N=64)
**as.factor(selvmordstanker_0)**		
0	57 (86.4%)	54 (84.4%)
1	5 (7.6%)	6 (9.4%)
Missing	4 (6.1%)	4 (6.3%)
**as.factor(selvmordstanker_16)**		
0	32 (48.5%)	41 (64.1%)
1	2 (3.0%)	5 (7.8%)
Missing	32 (48.5%)	18 (28.1%)

Analysis

Relative risk derived by G-computation RR: 1.98 (sRR: 2.03) Confidence interval: 0.42 - 333794883.75 p-value: 0.3807 It took 72 seconds.

TOCS - participants

Assumptions for linear regression

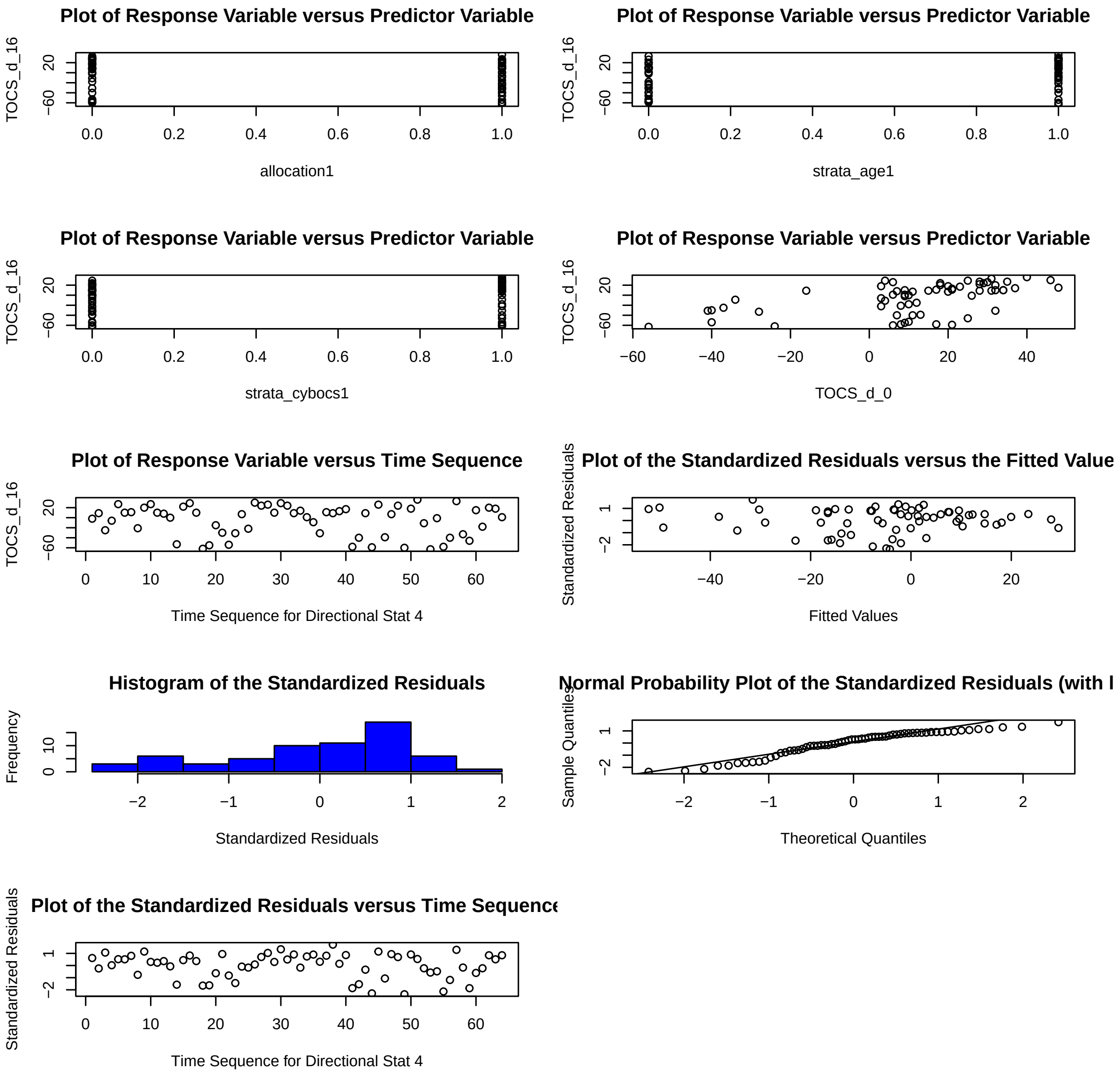

	Value	p-value	Decision
Global Stat	10.3914587	0.0343254	Assumptions NOT satisfied!
Skewness	5.9324020	0.0148650	Assumptions NOT satisfied!
Kurtosis	0.3687832	0.5436683	Assumptions acceptable.
Link Function	1.6102186	0.2044611	Assumptions acceptable.
Heteroscedasticity	2.4800549	0.1152982	Assumptions acceptable.

Summary data

	0	1	Overall
	(N=66)	(N=64)	(N=130)
**TOCS_d_0**			
Median [Q1, Q3] [Min; Max]	11.0 [5.00, 23.5] [-54.0; 48.0]	17.0 [3.00, 28.0] [-56.0; 53.0]	14.0 [3.00, 25[illegible]
Missing	15 (22.7%)	15 (23.4%)	30 (23.1%)
**TOCS_d_16**			
Median [Q1, Q3] [Min; Max]	7.00 [-27.5, 19.0] [-60.0; 33.0]	-3.00 [-32.5, 12.5] [-63.0; 45.0]	1.00 [-31.0, 1[illegible]
Missing	35 (53.0%)	22 (34.4%)	57 (43.8%)

Analysis

```
	Wilcoxon rank sum test with continuity correction

data:  TOCS_d_16 by allocation
W = 713.5, p-value = 0.4889
alternative hypothesis: true location shift is not equal to 0
95 percent confidence interval:
 -8.999994 18.999976
sample estimates:
difference in location
              5.000015
```

TOCS - parents

Assumptions for linear regression

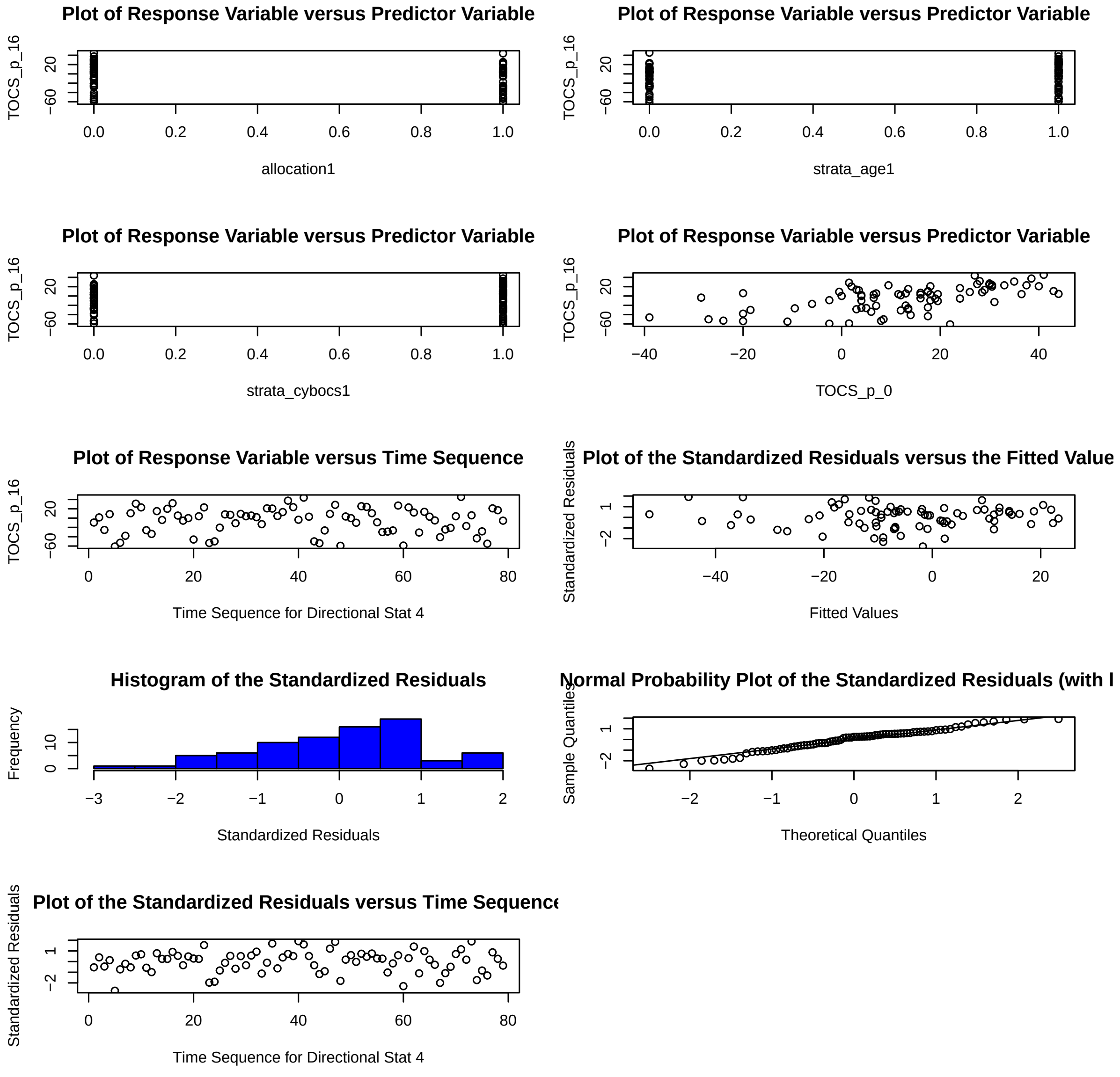

	Value	p-value	Decision
Global Stat	5.5563049	0.2348244	Assumptions acceptable.
Skewness	2.3848669	0.1225153	Assumptions acceptable.
Kurtosis	0.0053838	0.9415084	Assumptions acceptable.
Link Function	2.6089903	0.1062594	Assumptions acceptable.
Heteroscedasticity	0.5570639	0.4554457	Assumptions acceptable.

Summary data

	0	1	Overall
	(N=66)	(N=64)	(N=130)
**TOCS__p__0**			
Mean (SD) [Min; Max]	13.4 (17.2) [-39.0; 48.0]	12.7 (18.2) [-28.5; 55.0]	13.0 (17.7) [-39.0; 55.0]
Missing	9 (13.6%)	7 (10.9%)	16 (12.3%)
**TOCS__p__16**			
Mean (SD) [Min; Max]	-1.99 (26.9) [-59.0; 45.5]	-7.99 (27.4) [-61.0; 44.0]	-5.02 (27.2) [-61.0; 45.5]
Missing	24 (36.4%)	21 (32.8%)	45 (34.6%)

Analysis

	TOCS__p__16		
Predictors	Estimates	CI	p
(Intercept)	-14.16	-25.40 – -2.93	**0.014**
allocation [1]	-4.94	-15.07 – 5.20	0.335
strata age [1]	3.75	-6.32 – 13.81	0.461
strata cybocs [1]	-2.62	-13.10 – 7.87	0.621
TOCS p 0	0.91	0.62 – 1.20	**<0.001**
Observations	79		
R^2 / R^2 adjusted	0.357 / 0.323		

FAS-PR

Assumptions for linear regression

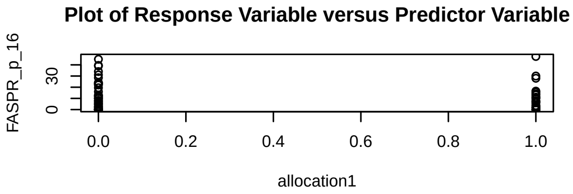

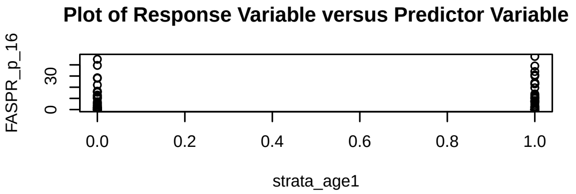

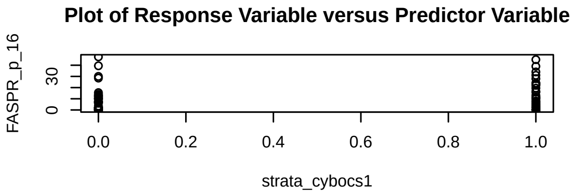

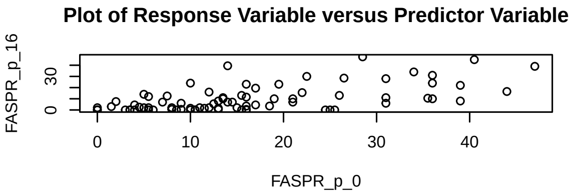

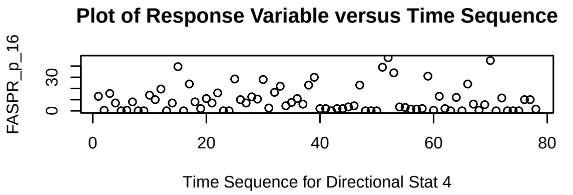

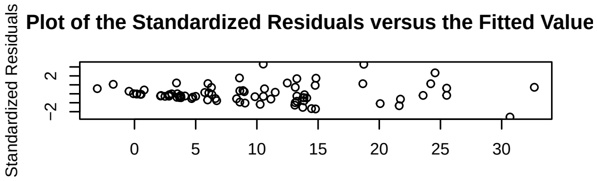

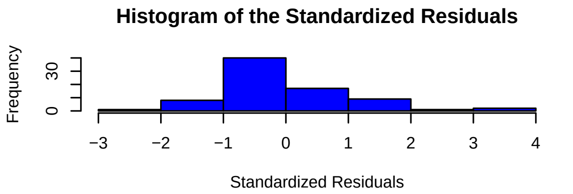

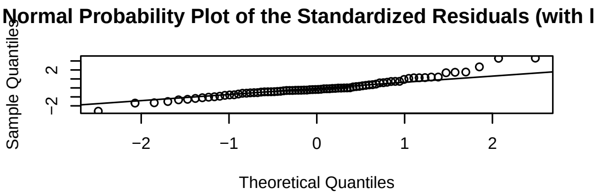

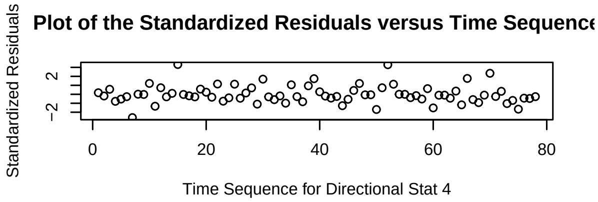

	Value	p-value	Decision
Global Stat	23.0305711	0.0001249	Assumptions NOT satisfied!
Skewness	10.0163681	0.0015516	Assumptions NOT satisfied!
Kurtosis	12.9708396	0.0003164	Assumptions NOT satisfied!
Link Function	0.0426315	0.8364207	Assumptions acceptable.
Heteroscedasticity	0.0007318	0.9784177	Assumptions acceptable.

Summary data

	0	1	Overall
	(N=66)	(N=64)	(N=130)
**FASPR__p__0**			
Median [Q1, Q3]	16.0 [9.25, 28.4]	14.0 [8.00, 26.0]	15.0 [8.25, 26.5]
Missing	8 (12.1%)	11 (17.2%)	19 (14.6%)
**FASPR__p__4**			
Median [Q1, Q3]	14.0 [6.00, 29.0]	11.0 [5.63, 19.4]	13.0 [5.75, 22.8]
Missing	17 (25.8%)	14 (21.9%)	31 (23.8%)
**FASPR__p__8**			
Median [Q1, Q3]	10.0 [5.13, 19.5]	8.25 [2.50, 21.0]	9.25 [4.00, 20.4]
Missing	20 (30.3%)	12 (18.8%)	32 (24.6%)
**FASPR__p__16**			
Median [Q1, Q3]	10.0 [2.38, 22.6]	2.00 [0, 10.3]	6.00 [0.500, 13.5]
Missing	26 (39.4%)	21 (32.8%)	47 (36.2%)

Analysis

```
    Wilcoxon rank sum test with continuity correction

data:  FASPR_p_16 by allocation
W = 1126, p-value = 0.01496
alternative hypothesis: true location shift is not equal to 0
95 percent confidence interval:
 0.4999294 9.4999828
sample estimates:
difference in location
               4.49994
```

PSS

Assumptions for linear regression

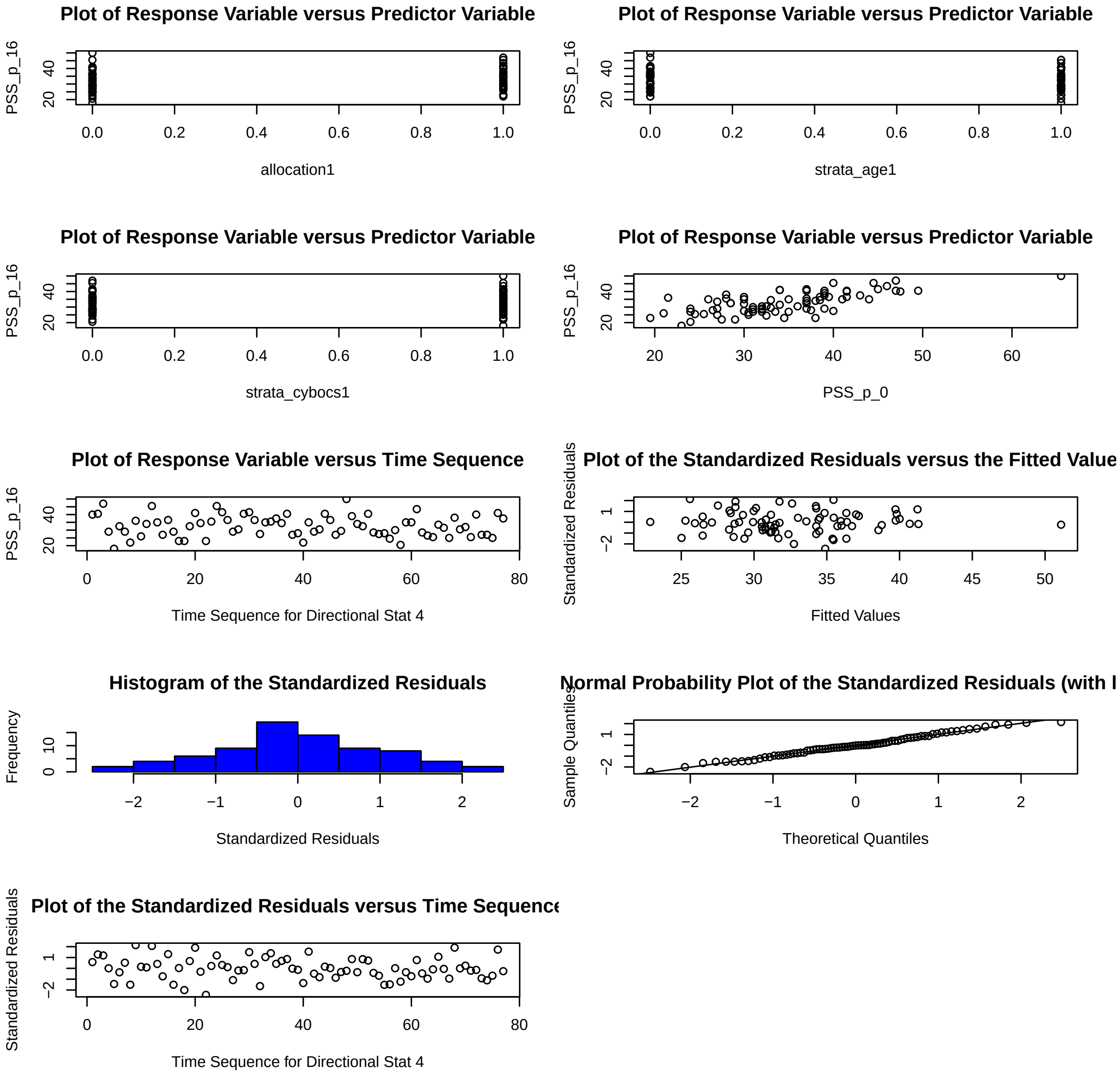

	Value	p-value	Decision
Global Stat	3.1911620	0.5263596	Assumptions acceptable.
Skewness	0.0498603	0.8233065	Assumptions acceptable.
Kurtosis	0.4469465	0.5037886	Assumptions acceptable.
Link Function	0.1746525	0.6760097	Assumptions acceptable.
Heteroscedasticity	2.5197027	0.1124318	Assumptions acceptable.

Summary data

	0	1	Overall
	(N=66)	(N=64)	(N=130)
**PSS_p_0**			
Mean (SD) [Min; Max]	34.4 (7.55) [19.5; 65.5]	36.0 (7.89) [19.0; 56.5]	35.2 (7.72) [19.0; 65.5]
Missing	8 (12.1%)	12 (18.8%)	20 (15.4%)
**PSS_p_4**			
Mean (SD) [Min; Max]	34.9 (8.16) [18.0; 56.0]	35.5 (6.33) [23.0; 48.5]	35.2 (7.30) [18.0; 56.0]
Missing	15 (22.7%)	16 (25.0%)	31 (23.8%)
**PSS_p_8**			
Mean (SD) [Min; Max]	34.2 (9.13) [18.0; 56.0]	35.1 (6.23) [23.0; 51.0]	34.7 (7.77) [18.0; 56.0]
Missing	17 (25.8%)	14 (21.9%)	31 (23.8%)
**PSS_p_16**			
Mean (SD) [Min; Max]	32.9 (8.41) [18.0; 59.0]	33.7 (6.48) [22.0; 47.0]	33.3 (7.45) [18.0; 59.0]
Missing	26 (39.4%)	22 (34.4%)	48 (36.9%)

Analysis

	PSS_p_16		
Predictors	Estimates	CI	p
(Intercept)	11.71	5.88 – 17.54	**<0.001**
allocation [1]	0.66	-1.65 – 2.96	0.572
strata age [1]	0.03	-2.33 – 2.38	0.983
strata cybocs [1]	-0.84	-3.20 – 1.52	0.481
PSS p 0	0.61	0.46 – 0.77	**<0.001**
Observations	77		
R^2 / R^2 adjusted	0.487 / 0.459		

Statistical report for the TECTO-trial Revision #1

Markus Harboe Olsen

May 5 2024

Table of contents

Therapist **2**
CYBOCS 16 - Adding therapist 2
Adding interaction term for therapist 3
Adding therapist as random effects 3

Therapist

	0<br>*n = 66*	1<br>*n = 64*
Therapist		
AMK	8 (12.1%)	2 (3.1%)
BBM	7 (10.6%)	9 (14.1%)
GHS	7 (10.6%)	12 (18.8%)
HKI	6 (9.1%)	5 (7.8%)
JH	3 (4.5%)	8 (12.5%)
KH	19 (28.8%)	19 (29.7%)
MSM	4 (6.1%)	1 (1.6%)
PZ	10 (15.2%)	5 (7.8%)
missing	2 (3.0%)	3 (4.7%)

CYBOCS 16 - Adding therapist

```
Analysis of Variance Table

Model 1: CYBOCS_16 ~ factor(allocation) + strata_age + CYBOCS_b
Model 2: CYBOCS_16 ~ factor(allocation) + strata_age + CYBOCS_b + Therapist
  Res.Df    RSS Df Sum of Sq      F Pr(>F)
1    104 6061.0
2     97 5371.9  7    689.17 1.7778 0.1004
```

Adding interaction term for therapist

```
Analysis of Variance Table

Model 1: CYBOCS_16 ~ factor(allocation) + strata_age + CYBOCS_b
Model 2: CYBOCS_16 ~ factor(allocation) + strata_age + CYBOCS_b + allocation *
    Therapist
  Res.Df    RSS Df Sum of Sq      F Pr(>F)
1    104 6061.0
2     90 5290.9 14    770.14 0.9357 0.5245
```

Adding therapist as random effects

	**0**<br>*n = 66*	**1**<br>*n = 64*	**Mixed effects linear regression**<br>*estimate (95%CI)*	*p*
CYBOCS_16			-3.11 (-6.00;-0.22)	0.038
median [IQR]	20.50 [15.00;25.00]	16 [11.00;20.50]		
mean (95%CI)	19.90 (17.65;22.16)	15.95 (13.69;18.21)		
missing	14 (21.2%)	5 (7.8%)		
