# Supplementary material for: Family-based cognitive behavioral therapy versus family-based psychoeducation and relaxation training for obsessive-compulsive disorder in children and adolescents: a randomized clinical trial (TECTO)
Source: Eur Child Adolesc Psychiatry. 2025 Jul 31;34(12):3955–71. doi: 10.1007/s00787-025-02797-4 (PMC12743107; doi:10.1007/s00787-025-02797-4)
Supplement: Supplementary file 3 — Supplementary Material 3 [file 787_2025_2797_MOESM3_ESM.pdf]

Caption for supplementary material:

Supplement-1

Details on compliance with regulatory requirements p.1

Details on informed consent procedures p.1

Details on masking procedures p.1

Details on common components of the interventions pp.1-2

Details on methods for fidelity and adherence ratings p.2

Details on ethnicity assessments p.2

Reference list for measurement instruments pp. 2-4

Items of the KIDSCREEN-10 index pp.4-5

Items of The Negative Effects Questionnaire Version 20 Items (NEQ-20) pp.6-7

Details on establishment of minimal clinically important difference (MCID) pp.7

Details on participant drop-outs (stopped treatment and further assessments) and treatment non-completion pp.8-10

Table-S1 Reasons for drop-out/treatment non-completion. Details on attendance in therapy sessions

p.11

Table-S2 Family Environment Scale (FES), baseline results pp.11-13

Table-S3 Negative treatment effects measured by NEQ-20, p.13

Details on change in exploratory outcomes from baseline to end-of treatment (week-16) pp.14

Table-S4 Change in psychopathological, functional, and family outcomes during trial pp.15-17

Figure-S1 Change in psychopathological, functional, and family outcomes during trial p.18

Table-S5 KIDCREEN-52, 10 dimensions, post-hoc analysis pp.19-21

Figure-S2 KIDSCREEN-52, 10 dimensions p.22

Moderating effect of therapy factors on treatment outcomes p.23-24

Table-S6 Therapy factors – confidence, motivation, alliance, and compliance pp.23-26

Results of manual fidelity ratings p.27

Table-S7 Comparison of mean fidelity ratings for 20 FCBT video-recordings and 20 FPRT video-recordings. pp.27-29

Supplement-2

Tables from the TECTO-trial, March 12, 2023

Statistical report for the TECTO-trial, October 28, 2022

Statistical report for exploratory analyses from the TECTO-trial

Statistical report for the TECTO-trial Revision #1, May 5, 2024
